# Temperature-related mortality: a systematic review and investigation of effect modifiers

**DOI:** 10.1088/1748-9326/ab1cdb

**Published:** 2019-07-09

**Authors:** Ji-Young Son, Jia Coco Liu, Michelle L Bell

**Affiliations:** 1 School of Forestry & Environmental Studies, Yale University, CT, United States of America; 2 Department of Biostatistics, Johns Hopkins Bloomberg School of Public Health, MD, United States of America

**Keywords:** effect modification, mortality, temperature, vulnerability

## Abstract

**Background:**

Understanding which populations are vulnerable and which factors affect vulnerability to temperature-mortality associations is important to reduce the health burden from current day weather extremes and climate change.

**Objectives:**

We reviewed population-based studies on the impact of temperature on mortality and assessed the vulnerability to temperature-mortality associations systematically.

**Methods:**

We identified 207 studies published between 1980 and 2017 and summarized findings on effect modification based on individual- and community-level characteristics.

**Results:**

In our assessment of vulnerability to temperature-mortality associations, we found strong evidence of effect modification for several individual-level factors such as age and sex. We also found limited or suggestive evidence for other individual-level factors such as education, place of death, occupation, race, marital status, and chronic conditions. Evidence on effect modification by community-level characteristics for temperature-mortality associations is limited. We found weak evidence of effect modification for population density, heating system, healthcare facilities, proximity to water, housing quality, and air pollution level. We found limited or suggestive evidence for community-level socio-economic status, latitude, urban/rural, air conditioning, climatic condition, green space, and previous winter mortality.

**Conclusions:**

Our findings provide scientific evidence on which populations could be targeted for establishing appropriate strategies to reduce the health burden from extreme temperatures, and for policies on climate change.

## Introduction

Temperature impacts on human mortality are a critical public health concern for the present day and with regard to climate change. Studies of temperature and mortality across many regions have demonstrated substantial epidemiologic evidence of increased risk of mortality from high or low ambient temperatures or extreme temperature events such as heat waves (Hajat and Kosatky 2010, Gasparrini et al 2015, Ryti et al 2016). Moreover, extreme weather events such as heat waves or droughts are expected to be more frequent and intense under climate change, with significant associated health impacts (McMichael et al 2006, Gershunov et al 2013, Seltenrich 2015).

Many studies of temperature-related mortality identified various subpopulations that have higher vulnerability to temperature effects or factors that affect vulnerability. The term ‘vulnerability’ is used in many different ways by various fields (Brooks 2003, Turner et al 2003, Adger 2006). An often-cited definition of vulnerability is the degree to which a system is susceptible to, or unable to cope with, adverse effects of climate change (IPCC 2001). Most epidemiological studies have used the terms ‘vulnerability’ (from environmental or external factors such as poverty and social inequality) and ‘susceptibility’ (from intrinsic biological factors such as genetics) for populations with disproportionate health burden (The Interagency Working Group on Climate Change (IWGCCH) 2010). Effect modification occurs when an exposure has a different health effect among different subgroups. For example, if a given level of air pollution had a higher impact on risk of a health outcome on persons of lower socio-economic status (SES) than richer populations, effect modification has occurred. Confounding occurs when a fact or is associated with both the exposure and the outcome but does not lie on the causative pathway. Confounding factors need to be addressed in analysis of exposure and health to prevent distortion of results, whereas effect modification provides important information. Effect modification is similar to statistical interaction, but in epidemiology, effect modification is related to the biology of disease, not just a data observation (Vander Weele 2012, Corraini et al 2017). In this study, we considered effect modifiers as individual-level or community-level factors related to susceptibility or vulnerability.

Several individual or environmental characteristics such as age (e.g. elderly, children), socioeconomic factors (e.g. income, education), and community-level factors (e.g. population density) have been reported to be associated with temperature’s effect on mortality (Benmarhnia et al 2015). Scientific evidence of vulnerability to temperature-related mortality can aid efforts to quantify the differences in risks across regions and populations and would benefit policy makers in developing the appropriate strategies to protect subpopulations. Better understanding of which populations or factors are related to vulnerability could inform knowledge on pathological mechanisms. Such evidence is also needed to estimate how climate change will impact human health, especially as population characteristics and environmental conditions change over time.

While previous literature reviews have focused on the impact of high temperature on several health outcomes (e.g. morbidity or mortality for respiratory or cardiovascular diseases) (Song et al 2017, Green et al 2019), fewer studies focused on susceptibility and vulnerability to temperature impacts on health outcomes (Hajat and Kosatky 2010, Benmarhnia et al 2015) and the results are inconclusive. Our review has several benefits in comparison to previous work. We considered all types of exposures for temperature such as high or low temperature and extreme events such as heat waves or cold spells, whereas other reviews focused on specific exposure metrics of temperature. We included all eligible studies conducted in any area of the world for a longer timeframe than earlier work, which could provide more information on the characteristics and patterns of findings generally (e.g. understudied region/countries, vulnerability patterns across time). Our review also considered a wide range of effect modifiers including individual- and community-level factors, which could address knowledge gaps for vulnerability factors (e.g. lack of evidence on some community-level effect modifiers). By systematically reviewing the existing evidence on the effect modification of temperature’s health impact on mortality, our study could help identify research needs and direction for future study. In this study, we systematically assess the vulnerability to temperature-mortality associations. We reviewed population-based studies regarding which populations are vulnerable and which factors affect vulnerability to temperature-mortality associations. We considered several individual and community characteristics.

## Methods

### Search strategy

We conducted a systematic search using a MEDLINE/PubMed database for population-based studies of exposure to heat or high temperature, cold, heat waves, and cold spells published between 1980 and 2017. We performed four searches: (1) (‘heat’ or ‘temperature’ or ‘cold’ or ‘heatwave’ or ‘heatwaves’ or ‘heat-wave’) AND (mortality or death); (2) modif* AND (effect or effects or impact or impacts) AND (‘heat’ or ‘temperature’ or ‘cold’ or ‘heatwave’ or ‘heatwaves’ or ‘heat-wave’); (3) (‘climate’ or ‘weather’ or ‘temperature’) AND (mortality or death); and (4) (‘climate’ or ‘weather’ or ‘temperature’) AND (effect or effects or impact or impacts) AND modif*; where * indicates any combination of subsequent letters. The systematic search was conducted with consideration of the PRISMA (Preferred Reporting Items for Systematic Reviews and Meta-analyses) guidelines (Moher et al 2015).

### Selection criteria

We selected studies meeting the following inclusion criteria. Studies had to: (1) be population-based; (2) consider exposure to heat or high temperature, cold, heat waves, or cold spells; (3) explore mortality; (4) examine effect modification; (5) be peer-reviewed; (6) be written in English; and (7) be published from 1980 to 2017, inclusive. Both single-city and multicity studies were included.

**Figure 1.**
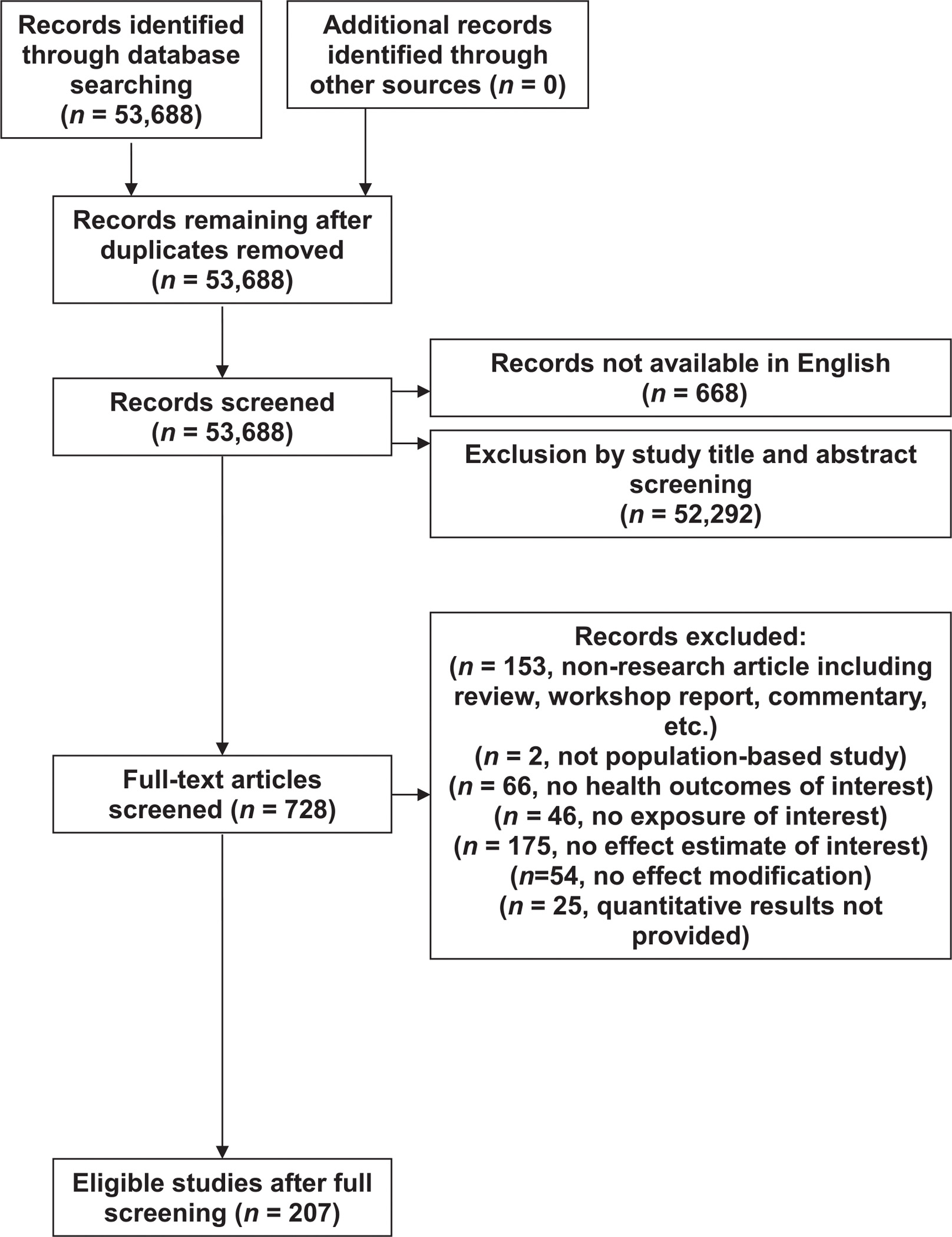
Flow diagram of literature selection process.

Next, we excluded studies by screening of titles and abstracts based on the inclusion criteria. We then reviewed the full texts of remaining articles. Figure 1 provides a flow diagram for the identification and selection of studies. We identified 728 studies for full-text screening. We extracted information of each article’s study location, time frame, health outcome (i.e. total, cardiovascular, and respiratory mortality), study population, temperature exposure metric (e.g. mean temperature, maximum temperature), increment of exposure used in effect estimates (e.g. unit increase, comparison of percentiles), lag structure (i.e. delayed effects of temperature on mortality) (e.g. previous day or average of previous days), effect modification studied, and results for main findings and effect modification factors. For results without quantitative results, such as graphical presentation of findings, we contacted the original study authors at least twice. We classified results into two categories: (1) studies investigating associations of single days of high or low ambient temperature with mortality and (2) studies investigating associations of consecutive days of extreme temperatures (i.e. heat waves, cold spells) with mortality. We used results from the key findings presented by study authors, as originally reported.

One of our objectives is to investigate effect modification of the association between ambient temperature and mortality. We included all effect modifiers from all eligible studies included in this review. We summarized findings on effect modification based on individual- and community-level characteristics. We identified the following potential individual- and community-level effect modifiers to temperature-mortality associations: (1) individual-level: sex, age, education, place of death (in or out of hospital), occupation, race, body mass index (BMI), marital status, and chronic conditions; and (2) community-level: SES based on income, gross domestic product (GDP), fraction living in poverty, or higher proportion of people living in low SES, population density, latitude, urban/rural, prevalence of heating systems, prevalence of air conditioning (AC), climatic condition, healthcare facilities, housing quality, proportion of green space or vegetation, proximity to water, previous winter mortality, air pollution, and reduced electricity consumption.

Most analyses of heat waves or cold spells compared risk during heat waves or cold spells to risk on non-heat wave or cold spell days, although conditions during heat waves or cold spells can vary. Therefore, some studies have investigated how the health impacts of heat waves or cold spells differ by heat wave or cold spell characteristics. We evaluated findings on effect modification of the heat wave/cold spell-mortality association, based on heat wave/cold spell characteristics as a modification of the exposure for heat waves/cold spells studies (e.g. length of heat waves). For effect modifiers, we summarized overall evidence using the following categories: no evidence, weak evidence, limited/suggestive evidence, and strong evidence. We assigned a category based on the quantity of studies providing consistent evidence compared with conflicting findings for the overall summary of evidence. A similar approach was used in previous studies (Bell et al 2014). We also provided the classification criteria for overall summary of evidence for effect modification (table S1 is available online at stacks.iop.org/ERL/14/073004/mmedia).

## Results

A total of 53 688 unique published articles were identified from the systematic search (figure 1). The first screening with study title and abstract review excluded 52 292 papers. Non-English papers were also excluded (668 papers). After the initial screening, 728 papers remained for full-text review. The major reasons for excluding studies were non-research article (e.g. review, commentary, etc), no effect estimate of interest (e.g. use of projected and simulated temperature, use of ambient temperature as a modification factor, examination of seasonal variation, etc) and no health outcomes of interest. The full-text screening resulted in 207 eligible studies for inclusion in our review.

Tables S2 and S3 in the supplemental material describe the characteristics of the studies included in this review. Table S2 includes the studies examining single days of heat and/or cold, whereas table S3 includes studies examining consecutive days of extreme heat (heat waves) or cold (cold spells). Some studies are listed in both tables S2 and S3. Each study’s time period and location, mortality outcome and exposure considered, exposure metric, lag structure, exposure increment, potential effect modifiers considered, and main findings were provided. We found 159 studies for heat and/or cold and 54 studies for heat waves and/or cold spells. Six studies investigated both heat and/or cold and heat waves and/or cold spells. Among the 159 studies for heat and/or cold, 139 studies assessed associations with total mortality. Ninety-four and 65 studies assessed the associations of heat and/or cold with cardiovascular and respiratory mortality, respectively. Forty-seven studies assessed the associations of heat waves and/or cold spells with total mortality, 31 studies with cardiovascular mortality, and 28 studies with respiratory mortality.

**Figure 2.**
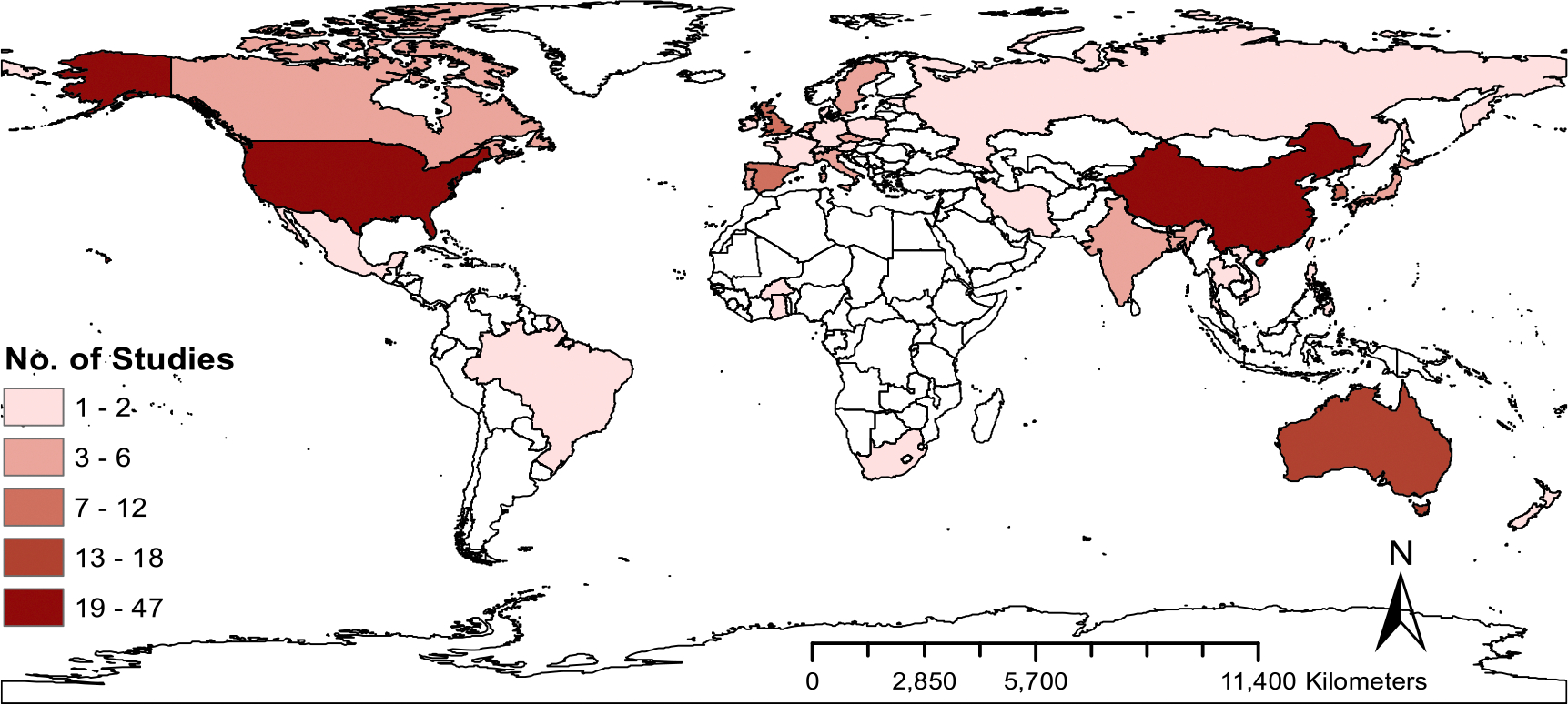
Number of studies on the relationship between ambient temperature and mortality, by country. Note: In addition, there were 16 multi-country studies(US 3; China 3; Greece 5; Spain 8; Hungary 6; Finland 5; United Kingdom 10; France 5; Italy 11; Sweden 5; Germany 2; Ireland 4; Slovenia 3; Czech Republic 3; Switzerland 2; India 1; Mexico 1; Canada 2; Thailand 2; Australia 3; Brazil 4; Taiwan 5; South Korea 5; Japan 5; Netherlands 1; Chile 1; Vietnam 1; Colombia 1; Iran 1; Philippines 1; Moldova 1).

Figure 2 shows the number of studies on the association between ambient temperature and mortality included in this review, by country. Most of the studies focused on Asia, North America, and Europe. Seventy-seven studies were conducted in Asia, 37 in North America, 52 in Europe, 3 in South America, 19 in Oceania, 3 in Africa, and 16 in multiple countries. The most represented country was China with 47 studies. Many nations, including most in South America and Africa, had no studies.

**Figure 3.**
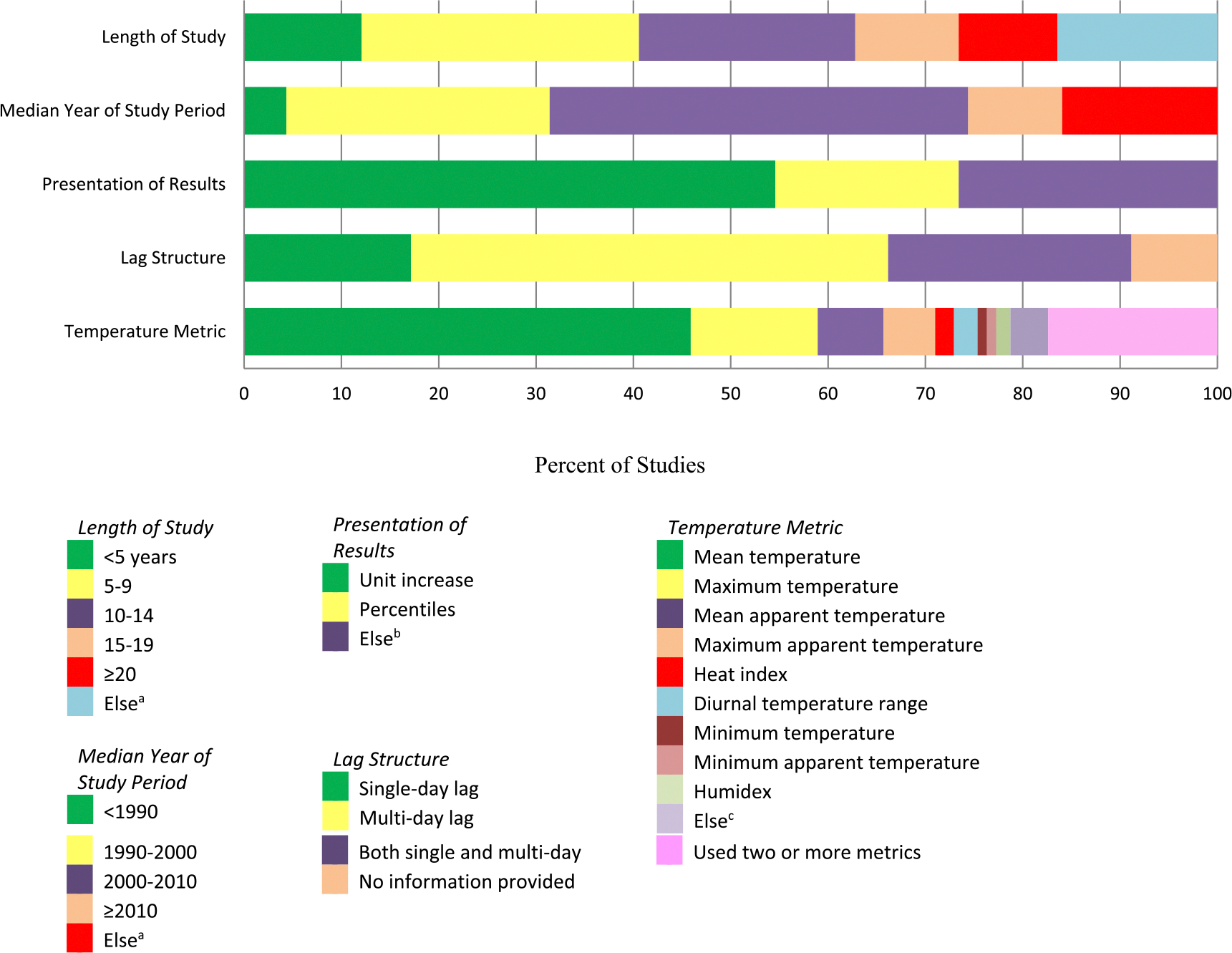
Summary of study characteristics across 207 studies of ambient temperature and mortality. Note: ^a^ e.g. different study period by city/country, two separate periods; ^b^ e.g. risk of mortality during heat waves/cold spells days compared to normal days; ^c^ e.g. temperature deviation index, spatial synoptic classification, universal thermal climate index.

Figure 3 summarizes study characteristics across 207 studies of ambient temperature and mortality. We summarized study characteristics based on length of study, median year of study period, presentation of results, lag structure, and temperature metric. Most study time periods were longer than 5 years and less than 10 years (28.5%). The most represented median year of the studies was within 2000–2010 (43.0% of the studies). Results were presented in several ways: 54.6% of studies reported percentage changes in risk associated with specified incremental increases or decreases in temperature (e.g. 1 °C, 10 °C, 10 °F) relevant to a specific threshold, and 18.8% of the studies reported percentage changes comparing two percentiles of temperature distributions (e.g. 50th versus 99th percentiles). For 26.6% of the studies, results were reported as the risk of mortality during heat waves/cold spells days compared with non-heat waves/cold spells days. For 49.0% of the studies, multiple lag structures were used; 25.0% used both single day lag and multiple day lag structures; and 8.8% of the studies did not provide information on the lag structure used. Various temperature metrics were used; mean temperature was the most commonly used exposure metric (45.9%), with 13.0% using maximum temperature. For 17.4% of the studies, two or more temperature metrics were used.

Table S4 provides disease categories and the diagnosis codes used to define mortality in the studies. Categorization of cause of death was not consistent across studies. Many studies used different codes to define the same disease. Total, cardiovascular, and respiratory mortality were identified through five or more different sets of International Classification of Diseases (ICD) codes. For total mortality, many studies (39.6%) provided no ICD codes.

Table 1 shows a summary of scientific evidence for effect modification on the temperature-mortality association. We examined findings of effect modification by individual and community characteristics. Risk estimates for heat were generally higher for women, with 37 studies finding higher effect for women and 12 studies finding higher effect for men. Twenty-five studies for heat found no difference in heat-mortality risk between men and women. For cold, we did not find strong evidence of higher associations in women or men. Effect modification by age was investigated in 142 studies for heat and 64 studies for cold. Categorization for age differed by study. Among the studies, 107 found higher risks from heat exposure for the elderly. Of the 64 studies on cold, 47 studies reported higher risks from cold exposure for the elderly. Twenty-six studies examined effect modification by education level for heat. Sixteen found higher risks for those with no or lower education compared to those with more education, with three studies finding higher risks for those with higher education, and seven studies finding no difference by education. For studies on the association between temperature and mortality we found limited or suggestive evidence of effect modification for the following individual-level characteristics: higher risk with out of hospital death for heat, higher risk with lower-level employment for heat, higher risk in Black/African-American populations for heat, higher risk for unmarried persons for heat, and higher risk with pre-existing conditions for heat and cold. There was weak evidence of higher risk with high BMI for cold.

Investigation of community-level effect modifications was limited. We found limited or suggestive evidence of higher temperature-mortality risks for communities with low SES for heat, in middle- or higher latitude regions for heat, in lower latitude regions for cold, in urban areas for heat, in rural areas for cold, with lower AC prevalence for heat, in communities with warmer climates for heat, in areas with lower proportion of green areas or vegetation for heat, and with low previous winter mortality for heat. There was weak evidence of higher associations for heat for communities with higher population density, poor healthcare facilities, far from the water, old and poor housing quality, and high air pollution. Weak evidence was observed for higher risks for cold for communities with lower prevalence of heating systems. No evidence of effect modification was observed for reduced electricity consumption. For studies on heat waves/cold spells, we found limited/suggestive evidence of higher risk with heat wave characteristics as a modification of the exposure (table S5).

## Discussion

In this systematic review, we evaluated published studies on the association between ambient temperature and mortality, with an emphasis on effect modification to identify factors affecting vulnerability. Findings of the studies we reviewed indicated that exposure to heat, cold, or extreme temperature events generally increased the risk of mortality. Findings also suggest that risks on the associations between ambient temperature and mortality may differ by cause of death and varied across a wide range of communities (e.g. different city within same country). In our assessment of vulnerability to temperature-mortality associations, we found evidence of effect modification for several individual-level factors such as age and sex.

## Individual-level effect modifiers

### Age

We identified age as the most consistent effect modifier of the association between ambient temperature and mortality, with higher risks from heat or cold exposure for the elderly. Vulnerability for the elderly may relate to physiological changes, different activity patterns, housing quality, and social factors. Older persons can have limited thermoregulatory response, different prevalence of comorbidities, live alone, different access to AC or heating systems, and limited access to healthcare facilities and social services (Hajat and Kosatky 2010, Bunker et al 2016). Exposure from heat or cold in the elderly may trigger adverse airway responses, increased inflammatory factors, and increased susceptibility to infection (Eccles 2002, Anderson et al 2013).

### Sex, education

We found strong evidence of higher temperature-mortality risks associated with heat exposure for women than for men. This may result from differences in physiology, exposure patterns and occupational exposure between men and women. However, some studies reported no difference in risk between men and women, or a higher estimated effect for men (Goggins et al 2013, Bai et al 2014, Zeng et al 2017). Education level is often used as an indicator of SES, and many studies reported higher mortality risks for those with no or low education (Borrell et al 2006, Kaiser et al 2007, Son et al 2012, Yang et al 2013, Bai et al 2014, Chen et al 2015, Huang et al 2015, Zeng et al 2017). However, some studies also identified no difference or higher risk for those with higher education level (Ma et al 2012, 2015, Wang et al 2014, Ding et al 2016, Isaksen et al 2016). Socioeconomic factors can be correlated with each other and one specific indicator does not fully represent actual SES, which can relate to many different factors such as multiple sources of income, family income, and historical SES (O’Neill et al 2003, Mechanic 2007). Both individual and community SES can play a role in health. Further, other demographic factors can be related to SES. For example, sex may be related with other SES factors. In our previous work in Korea, women study participants were older and less educated (Son et al 2012). Future research considering multiple SES indicators and related factors, and the relationships among these factors, is needed to better understand how they modify the temperature-mortality association.

### Other individual-level factors

We also found limited or suggestive evidence of effect modification for other individual-level factors such as place of death, occupation, race, marital status, and chronic conditions. Findings suggest higher risks with out of hospital death (Medina-Ramón et al 2006, Son et al 2011, 2016a Huang et al 2015, Ma et al 2015, Zhang et al 2016), with lower level employment (Anderson and Bell 2009, Yang et al 2012, 2013, Xu et al 2013, Heo et al 2016), in black populations (O’Neill et al 2005, Medina-Ramón et al 2006, Kaiser et al 2007, Madrigano et al 2015a, Lee et al 2016), and for unmarried persons (Stafoggia et al 2006, 2008, Schifano et al 2009, Gronlund et al 2015, Qiu et al 2016) from heat and/or cold exposure.

## Community-level effect modifiers

We found that evidence on effect modification by community-level characteristics is limited, identifying gaps in the literature. We found weak evidence for effect modification based on population density, heating system, healthcare facilities, proximity to water, housing quality, and air pollution level. We found limited or suggestive evidence for effect modification based on community-level demographics and SES, latitude, urban/rural, AC, climatic condition, proportion of green areas or vegetation, and previous winter mortality. For heat waves/cold spells studies, we also found limited or suggestive evidence of higher risk with heat wave characteristics such as more intense, longer duration, or earlier in summer (Anderson and Bell 2011, Son et al 2012).

### Heat/heat waves

Higher heat-related mortality risk was associated with higher population density (Medina-Ramón and Schwartz 2007, Ma et al 2015, Madrigano et al 2015b), lower prevalence of heating systems (Curriero et al 2002), poor healthcare facilities (Huang et al 2015), far from the water (Burkart et al 2016), old and poor housing quality (Xu et al 2013), and high air pollution levels (Ren et al 2008, Breitner et al 2014, Li et al 2015). Studies used a range of variables to define community-level SES including indirect variables anticipated to be correlated with SES: multiple indicators, income, GDP, poverty, and proportion of elderly, Black/African-American, or low educated people. Findings suggest higher heat-related mortality risk with low community-level SES (Wichmann et al 2011, Ma et al 2014, Rosenthal et al 2014, Lim et al 2015, Madrigano et al 2015b), in middle- or higher latitude (Curriero et al 2002, Ma et al 2014, Xiao et al 2015, Lim et al 2016), in urban areas (Anderson and Bell 2009, Burkart et al 2014, Urban et al 2014, Ma et al 2015), with lower AC prevalence (O’Neill et al 2005, Medina-Ramón and Schwartz 2007, Anderson and Bell 2009, Basu and Malig 2011, Nordio et al 2015, Chen et al 2016), in communities with warmer climates (Rosenthal et al 2014, Huang et al 2015, Madrigano et al 2015a), in areas with less proportion of green areas or vegetation (Xu et al 2013, Ma et al 2014, Gronlund et al 2015, Madrigano et al 2015a, Burkart et al 2016, Son et al 2016b), and with low previous winter mortality (Rocklöv et al 2009, Stafoggia et al 2009, Ha et al 2011, Qiao et al 2015).

### Cold/cold spells

Relatively less evidence was reported for risk from cold or cold spells than risk of exposure from high ambient temperature or heat waves. Some studies reported that effects from cold temperature are higher in regions with warmer climate (Curriero et al 2002, Ma et al 2014). Findings suggest higher cold-related mortality risk in lower latitude (Curriero et al 2002, Ma et al 2014, Xiao et al 2015), and in rural areas (Zhou et al 2014, Urban et al 2014). Although we found weak or limited/suggestive evidence on effect modification by some community-level factors, these results reflect the current scientific evidence, which is related to the lack of a sufficient number of adequate studies. More work investigating the community-level effect modification on the temperature-mortality association is required to fully understand this issue, and our findings give directions for future work.

Our review identified several challenges to comparing and summarizing results across the studies. Many studies used different definitions to define a disease or exposure. Each mortality cause (i.e. total, cardiovascular, and respiratory mortality) were identified through five or more different sets of ICD codes, an issue that has been previously identified (Ji et al 2011). To define heat waves or cold spells, many studies applied various definitions, and there exists no standard definition of a ‘hot day’ or ‘heat wave’, etc in scientific research or in policy.

Our findings showed that most studies on the association between ambient temperature and mortality were based on areas in Asia, Europe and North America. Current epidemiologic evidence is very limited in many other regions. There is substantial epidemiologic evidence that ambient temperature is associated with mortality and that the relationship is nonlinear across many regions with different climate and populations (Guo et al 2016, Ryti et al 2016). However, the magnitude of the associations differs by several characteristics such as population structure and region. Spatial variability in effect estimates across studies may relate with several factors such as regional climate, population characteristics (e.g. population’s sex or age structure), presence of heat warning systems, and acclimatization to local climate. Thus, more studies are needed worldwide, especially in understudied regions, and should be conducted in more varied climate zones to better understand the association between ambient temperature and mortality outcomes and to allow further investigation of effect modification.

We were unable to quantify the overall effect modification for the mortality risk from exposure to heat, cold, or extreme temperature events across studies. There were insufficient studies with similar approaches to conduct meta-analysis by each category. However, we provided detailed information such as mortality category, location, exposure metric and increment, and effect modifiers studied for each study. This information from our review provides guidance on which effect modifiers and populations show consistent patterns in general although there may exist differences across regions and these findings could help identify critical areas for future study. One of our most important contributions is that findings for effect modifiers varied by region and population, which highlights the importance of local studies. To provide location-specific information for governments to identify target areas and most effectively use resources, future study such as vulnerability mapping is needed regarding which populations and areas are vulnerable to temperature-mortality associations in a given location. Publication bias may exist; studies that did not find statistical significance may be selectively submitted or published. There may be other relevant studies exploring important issues of risk of mortality in relation to metrics of temperature and effect modification that did not include in this review due to our selection criteria.

In our review, we found that some characteristics such as AC, housing quality, pre-existing conditions may associate with increased vulnerability. Disparities in temperature-mortality association could be mediated by various factors (Gronlund 2014). Providing evidence on the behavioral modifiers such as individual’s actions or ability to reduce the exposure, physiologic changes or adaptation, which may vary by climate zone could play a critical role for decision makers to develop appropriate interventions and policies. Future research using multiple relevant disciplines and resources may contribute to better understanding of the characteristics of vulnerability, mechanisms, and potential mediators of these relevant characteristics and risk factors.

The effect modifiers identified in this review such as SES include multiple components and may influence the risk of mortality through multiple pathways such as environmental exposure, behavior/lifestyle, and healthcare resources. Previous study evaluated whether areas with higher heat vulnerability experienced higher rates of morbidity and mortality on abnormally hot days. They found that heat vulnerability index may be an indicator of overall health vulnerability, regardless of exposure to heat, although greater vulnerability to heat was observed in some areas (Reid et al 2012). Variability in health risk across studies may result from differences in location characteristics, methodologies used, and population structure over time. Understanding local differences about which variables contribute most in some regions but not in other regions is critical thus future studies could consider local information for assessing vulnerability at local scales.

To our knowledge, this is the largest systematic review of effect modification of the temperature effect on mortality considering heat, cold, and extreme temperature events with a focus on vulnerability. Consistent with our findings, previous reviews reported several factors with higher vulnerability. A study by Hajat and Kosatky (2010) suggested that aging population, higher population density, and lower GDP were associated with higher risk of heat exposure on mortality. Another review found strong evidence of heat-related vulnerability for the elderly and low SES groups (Benmarhnia et al 2015). Our findings provide evidence on which populations are most vulnerable and which factors affect vulnerability for the temperature-mortality association, and identify critical areas for future study. Better understanding about the vulnerable populations for the association between ambient temperature and mortality benefits policy makers in establishing appropriate strategies to reduce the health burden from current day weather extremes. Findings also inform our understanding of the consequences of climate change. Future studies could consider the impacts of temperature on health under a changing climate in relation to vulnerable populations and other relevant effect modifiers, and temporal changes in these factors.

## Supplementary Material

supplement

## Figures and Tables

**Table 1.** Summary of scientific evidence for effect modification on the temperature-mortality association.

Effect modifier	Heat/Heat waves	Cold/Cold spells	Summary of evidence

Individual-level			
Sex			Strong evidence of higher risk for women for heat
Women	37 studies ↑; 25 studies –	9 studies ↑; 15 studies –	
Men	12 studies ↑	8 studies ↑	
Age			Strong evidence of higher risk for older persons for heat and cold
Elderly	107 studies ↑; 15 studies –	47 studies ↑; 8 studies –	
Children	11 studies ↑	6 studies ↑	
Adults	9 studies ↑	3 studies ↑	
Education			Limited or suggestive evidence of higher risk with low education for heat and cold
None or low	16 studies ↑;7 studies –	6 studies ↑;3 studies –	
High	3 studies ↑		
Place of death			Limited or suggestive evidence of higher risk with out of a hospital death for heat
Out of a hospital	6 studies ↑; 2 studies –	3 studies ↑	
Home	4 studies ↑		
Occupation			Limited or suggestive evidence of higher risk with lower-level employment for heat
Manual or blue-collar workers	6 studies ↑; 1 study –	1 study ↑	
Unemployed	1 study ↑	1 study ↑	
Race			Limited or suggestive evidence of higher risk in Black, African/American populations for heat
Black, African / American	6 studies ↑; 1 study –	1 study ↑	
Hispanic	1 study ↑		
BMI			Weak evidence of higher risk with high BMI for cold
High		1 study ↑	
Marital status			Limited or suggestive evidence of higher risk with unmarried status for heat
Unmarried	2 studies ↑; 3 studies –		
Unmarried, divorced, or widowed	2 studies ↑	1 study ↑	
Widow	1 study ↑	1 study ↑	
Chronic conditions			Limited or suggestive evidence of higher risk with pre-existing conditions for heat and cold
People with pre-existing conditions	7 studies ↑	6 study ↑	
Community-level			
SES			Limited or suggestive evidence of higher risk with low SES for heat
High^a^	2 study ↑; 4 studies –	1 study ↓; 1 study –	
Low^a^	9 studies ↑; 1 study –	3 study ↑; 1 study –	
Population density	3 studies ↑		Weak evidence of higher risk with higher population density for heat
Latitude			Limited or suggestive evidence of higher risk in middle or higher latitude for heat, and in lower latitude for cold
Lower	1 study ↑	4 studies ↑	
Middle or higher	6 studies ↑		
Urban/rural			Limited or suggestive evidence of higher risk in urban areas for heat, in rural areas for cold
Urban	4 studies ↑; 2 studies –	1 study ↑; 1 study ↓	
Less urban/Rural	2 studies ↑	4 studies ↑	
Air conditioning			Limited or suggestive evidence of higher risk with lower AC prevalence for heat
Lower prevalence	4 studies ↑	1 study ↑	
Higher prevalence	4 studies ↓	1 study ↑	
Heating system		1 study ↓	Weak evidence of lower risk with higher prevalence of heating system for cold
Climatic condition			Limited or suggestive evidence of higher risk in communities with warmer climates for heat
Communities with warmer climates, higher surface temperature	12 studies ↑	2 study ↑	
High relative humidity, weak wind	2 studies ↑		
Strong wind		1 study ↑	
Healthcare facilities			Weak evidence of lower risk with better healthcare facilities
Better healthcare facilities	1 study ↓; 1 study –	1 study ↓	
Green space			Limited or suggestive evidence of higher risk in areas with less proportion of green areas or vegetation for heat
Less proportion of green areas	6 studies ↑; 1 study –		
Blue space			Weak evidence of lower risk with closer proximity to water for heat
Closer proximity to water	1 study ↓		
Housing quality	3 studies ↑		Weak evidence of higher risk with old and poor housing quality for heat
Air pollution			Weak evidence of higher risk with high air pollution for heat
High PM_10_	2 study ↑; 2 studies –	1 study ↑	
High ozone	2 studies ↑; 1 study –		
Previous winter mortality	4 studies ↑		Limited or suggestive evidence of higher risk with low previous winter mortality for heat
Reduced electricity consumption	1 study –		No evidence of effect modification

a SES group based on several variables such as child mortality rate, the child/woman ratio, the literacy rate, the fertility rate, the sources of drinking and non-drinking water, infant mortality, the insolvency rate, and use of solid fuels; income; GDP; living in poverty; or higher proportion of people living in low socio-economic status (SES).

Note: The meaning of the arrows and symbol are as follows: ↑ indicates higher risk; ↓ indicates lower risk; and – indicates similar effect, no difference in effects, and/or no effect modification. Some studies did not provide information on statistical significance, in which case we used results from the key findings as presented by study authors.
