# Supplementary material for: Temperature-related mortality: a systematic review and investigation of effect modifiers
Source: Environ Res Lett. Author manuscript; Available in PMC 2025 Aug 20. (PMC12362558; doi:10.1088/1748-9326/ab1cdb)
Supplement: supplement [file NIHMS2098800-supplement-supplement.pdf]

Temperature-related mortality: A systematic review and investigation of effect modifiers

Ji-Young Son, Jia Coco Liu, Michelle L. Bell

Table of Contents

Table S1. Classification criteria for overall summary of evidence for effect modification

Table S2. Description of studies of heat and cold exposure and mortality included in the review

Table ~~S2~~S3. Description of studies of heat waves and cold spells and mortality included in the review

Table ~~S3~~S4. Disease categories and diagnosis codes in studies

Table ~~S4~~S5. Summary of scientific evidence for effect modification on the temperature-mortality association

Table S1. Classification criteria for overall summary of evidence for effect modification

Classification	Criteria
Strong evidence	- Findings generally consistent in direction and size of effect or degree of association<br>- Large number of studies<br>- Consistent evidence from at least 30 independent studies compared with conflicting findings
Limited/Suggestive evidence	- Low number of studies<br>- Consistent evidence from at least 4 independent studies compared with conflicting findings
Weak evidence	- Limited number of studies<br>- Consistent evidence from at least 1 independent study compared with conflicting findings
No evidence	- Insufficient number of studies<br>- No consistent evidence

Table S1S2. Description of studies of heat and cold exposure and mortality included in the review

Studies	Period	Location	Mortality (ICD codes)	Exposure Type	Exposure Metric	Lag	Exposure Increment	Effect Modification Studied	Main Findings
**Asia**									
Bai et al. 2014	2008-2012	3 Tibetan counties, China	non-accidental (A00-R99); cardiovascular (CVD) (I00-99)	heat, cold	mean temperature	lags 0, 0–2, 0–7, 0–14 days	heat effect: 99th percentile relative to 75th percentile; cold effect: 1st vs. 25th percentile	age, sex, education	cumulative cold effects in Jiangzi (relative risk (RR) 2.251, 95% CI 1.054–4.849); more vulnerable to temperature extreme in men, elderly (> 65 yrs), illiterate persons
Ban et al. 2017	2013-2015	43 counties, China	non-accidental (A00-R99); circulatory (I00-I99); respiratory (J00-J99)	heat	mean temperature	lag 0-2, 0-3, 0-7	For relative temperature changes: 99th percentile relative to 90th percentile, 90th vs. 75th percentile; For absolute temperature changes, at 27.3 °C (90th percentile temperature of all counties) compared to 23.8 °C (75th percentile temperature of all counties)	age, sex	All observed cause-specific mortalities are significantly associated with higher temperature; Estimated effects of high temperature on mortality varied by spatial distribution and temperature patterns; Overall RR comparing 99th to 90th temperature percentiles for non-accidental mortality is 1.105 (95% CI: 1.089, 1.122), for circulatory disease is 1.107 (95%CI: 1.081, 1.133), and for respiratory disease is 1.095 (95%CI: 1.050, 1.142).
Burkart et al. 2014	not specified	Bangladesh, Nepal	all-cause; CVD	heat	universal thermal climate index (UTCI)	lag 0-1, lag 0-4	per 1°C increase in UTCI above threshold	age, sex, location (rural/urban), SES	all-cause mortality above threshold of 31.3% (95% CI 24.5–44.3)
Chen et al. 2016	May to Sep., 2009-2013	102 counties in Jiangsu Province, China	total (A00-R99); cardiorespiratory (I00-I99, J00-J99)	heat	daily mean temperature	lag 0 to lag 6	risk at 99th percentile relative to 75th percentile	urban/nonurban areas, county-level characteristics (population, % of urban population, % of people ≥ 65 years (yrs) old, % of unemployed people, average years of education, number of air conditioning (AC) units per household, number of beds in health institutions per 1,000 people, gross domestic product (GDP), and revenue of local government)	Overall mortality risk comparing 99th vs. 75th percentiles of temperature was 1.43 (95% posterior interval (PI): 1.36, 1.50) in less urban counties and 1.26 (95% PI: 1.23, 1.30) in more urban counties; Heat effects on cardiorespiratory mortality followed a similar pattern. Higher education level and prevalence of AC significantly associated with counties having lower risks, whereas % of elderly people significantly associated with increased risks.

Cheng et al. 2014	2008-2012	Maanshan, China	non-accidental (A00-R99); CVD (I00-I99); respiratory (J00-J99)	heat, cold	difference between current and previous days' mean/max/min temperature	lags 0, 1, 2, 3, 7, 14, 21	temperature increase/decrease (1, 5, 25, 75, 95, and 99th percentiles of temperature changes) along with 0°C as the reference	sex, age	3% (95% CI 0–5 %) and 8% (2–15 %) increase per 1.9°C increase in max temperature change in non-accidental and CVD mortality, respectively
Cui et al. 2016	2011-2014	Chengdu City, China	non-accidental; CVD; respiratory	heat, cold	mean temperature	lag 0-21	temperatures above and below an optimum temperature corresponding to the MMT	sex, age (0-64, 65+ yrs)	Overall, total fraction of deaths caused by both heat and cold was 10.93% (95% CI: 7.99–13.65%); cold was responsible for more of the burden than heat (9.96%, 95% CI: 6.90–12.81%, vs. 0.97%, 0.46–2.35%); The attributable risk (AR) was higher for respiratory (19.69%, 14.45–24.24%) than CVD mortality (11.40%, 6.29–16.01%).
Dang et al. 2016	2009-2013	Hue, Viet Nam	all-cause; non-external (A00-R99); CVD (I00-I99); respiratory (J00-J99)	heat, cold	Tmin, average, Tmax	lag 0 to lag 28; lag 0-2 for heat; lag 0-28 for cold	heat: RR comparing the 99th percentile (32.4°C) to the 50th (26.3°C); cold: RR comparing the 1st (15.8°C) to the 50th (26.3°C) percentile	sex, age (0-64, 65+ yrs)	Elevated risk in vulnerable groups: elderly people (heat effect, RR 1.42, 95% CI 1.11-1.83; cold effect, RR 2.0, 95% CI 1.13-3.52), females (cold effect, RR 2.19, 95% CI 1.14-4.21), people with respiratory disease (heat effect, RR 2.45, 95% CI 0.91-6.63), and those with CVD (heat effect, RR 1.6, 95% CI 1.15-2.22; cold effect, RR 1.99, 95% CI 0.92-4.28).
Ding et al. 2016a	2009-2014	Yuxi, China	non-accidental (A00-R99); CVD (I00-I99); respiratory (J00-J99)	cold	mean temperature	lag 0-21	1st percentile relative to 10th temperature percentile; a 1°C decrease below a cold threshold	gender, age, marital status, ethnicity, occupation, or previous history of hypertension	Cold temperature associated with increased mortality; RR of cold effect (1st vs. 10th percentile) of non-accidental, CVD, and respiratory mortality was 1.40 (95% CI: 1.19-1.66), 1.61 (1.28-2.02), and 1.13 (0.78-1.64), respectively. A 1°C decrease < cold threshold of 9.1°C (8th percentile) was associated with a 7.35% (3.75-11.09%) increase in non-accidental mortality. Cold-mortality association not significantly modified by cause-specific mortality, gender, age, marital status, ethnicity, occupation, or history of hypertension.

Ding et al. 2016b	2007-2014	Yuxi, China	non-accidental (A00-R99); CVD (I00-I99); respiratory (J00-J99)	heat	diurnal temperature range (DTR)	lag 0, lag 0-2	High DTR days defined as ≥ 90th percentile of DTR; risk of mortality on high DTR days compared with the reference group	age (≤64, 65-74, 75-84, 85+ yrs), gender, ethnicity, marital status, occupation, level of education, and previous history of hypertension	Risk of mortality on high DTR days was associated with age 75-84 yrs (odds ratio (OR) 1.07; 95% CI 1.01-1.14) and ≥ 85 yrs (1.16; 1.08-1.24) compared with age ≤ 64 yrs. The risk was less for the Dai ethnic minority than Chinese Han. Farmers (1.08; 1.03-1.14) and people with hypertension (1.09; 1.02-1.16) showed greater risk of dying on high DTR days than non-farmers and people without hypertension, respectively.
El-Zein et al. 2004	1997-1999	Beirut on eastern coast of the Mediterranean	total	heat, cold	mean daily temperature	up to 2 weeks	1°C increase in temperature above and below MMT (27.5°C)	age	12.3% increase (95% CI 5.7–19.4%) and 2.9% decrease (2–3.7%) in mortality, above and below MMT, respectively
Gao et al. 2017	June to August, 2008–2011	Harbin, China	cardio-cerebrovascular (I01-I69.8); CVD (I01-I51.9); cerebrovascular (I60-I69.8); acute CVD (I01, I21-I22, I24, I30.9, I33.0, I40.9); acute cerebrovascular (I60-I64); acute cardio-cerebrovascular (I01, I21-I22, I24, I30.9, I33.0, I40.9, I60-I64)	heat	mean temperature	lag 0, 0-1, 0-2, 0-3, 0-4, 0-5, 0-6, 0-7	each 1 °C increase above the threshold temperature	gender, age	The acute CVD mortality seemed to be more sensitive to temperature than CVD mortality with higher mortality risk and shorter time lag effects.
Goggins et al. 2013	May-Oct., 1999-2008	Kaohsiung, Taiwan	natural	heat	daily mean temperature	lag 0-4	1°C above/below 29°C	age, district level	each 1°C increase in mean temperature above 29.0°C significantly associated with 3.4% (95% CI 0.6, 6.3%) increase in mortality
Goggins et al. 2012	June-Sep., 2001-2009	Hong Kong	natural	heat	mean temperature	lag 0-4	1°C increase above 29°C	SES, high (above the median) or low urban heat island index (UHII) of residence	4.1% (95% CI 0.7, 7.6%) increase in mortality in areas with high UHII; 0.7% (22.4%, 3.9%) increase in low UHII areas

Guo et al. 2012	1999-2008	Chiang Mai city, Thailand	non-external (A00–R99); cardiopulmonary (I00–I99, J00–J99); CVD (I00–I99); respiratory (J00–J99)	heat, cold	mean temperature	lag 0, 0-1, 0-2, 0-3, 0-7, 0-13, 0-21	cold: 1st percentile relative to 25th percentiles (19.35°C vs. 24.7°C); heat: 99th vs. 75th percentiles (31.7°C vs. 28°C)	age	RR of non-external mortality with cold temperature: 1.29 (95% CI 1.16-1.44); with high temperature: 1.11 (1.00-1.24)
Ha et al. 2011a	June-Aug., 1992-2007, except 1994	Seoul, Daegu, and Incheon, South Korea	all-cause (A00-R99) in all cities; CVD (I00-I99) for Seoul only	heat	daily mean temperature	lag 0-1	1°C increase in avg summer temperature above thresholds	previous winter mortality, age	associations stronger in summers preceded by winters with low vs. high mortality levels for all non-accidental deaths.
Ha et al. 2011b	June–Aug., 1991-2008	Seoul, Daegu, and Incheon, South Korea	all-cause (A00–U99) for all cities; CVD (I00–I99) for Seoul only	heat	daily mean temperature	30 days	1°C increment above threshold	age	high temperature associations with mortality continued for about 5 days; 30 days after high temperature exposure, cumulative effects were still high in Seoul and Incheon.
Ha et al. 2009	Dec.-Feb., 1994-2006	Seoul, South Korea	all-cause (A00-U99); cardiorespiratory (I00–I99, J00–J99); CVD (I00–I99)	cold	cold wave index (CWI)	lag 0, 1, 2	1°C decrease < threshold	age	effects of daytime CWI lagged by 0-2 days were the strongest. The most significant mortality outcomes were CVD-related. Those ≥65 yrs were more vulnerable.
Ha and Kim 2013	1993–2009	Seoul, South Korea	all-cause (A00–U99); CVD (I00-I99)	heat	daily mean temperature	lag 0-1	1°C increase in summer temperatures above threshold	age, summer group (all, early, late summer)	temperature-related mortality during summer over past 17 yrs has declined, but significant association remained; declines in temperature-related mortality particularly noteworthy for late summer.
Hashizume et al. 2009	1994-2002	Matlab, Bangladesh	all-cause excluding external causes; CVD (390-459); respiratory (460-519)	heat, cold	daily avg temperature	heat: lag 0-1; cold: lag 0-13	per 1°C increase/decrease in mean temperature above/below threshold	age	cold: 3.2% (95% CI 0.9–5.5%) increase in all-cause mortality; however, no clear heat effect on all-cause mortality for any lag.
Heo et al. 2016	1996-2000, 2008-2012	South Korea	all-cause except accidents (V00-Y99); CVD (I00-I99); respiratory (J00-J99)	heat	Tmax	lag 0, 1, 2, 3, 4-7	1 °C increase above the threshold	heat cluster, sex, education, job status	Temporal increases in mortality risk were larger for some subgroups: those <75 yrs, those with a lower education and blue-collar workers, in hottest cluster as well as all combined regions

Huang et al. 2015	2006–2011	66 Chinese communities	non-accidental (A00-R99); CVD (I00-I99); respiratory (J00-J99)	heat, cold	daily mean temperature	heat: lag 0-2; cold: lag 0-21	cold: 1°C decrease in temperature below MMT; heat: 1°C temperature increase above MMT	individual factors (age, sex, education, place of death, cause of death); community-level factors (annual temperature, population density, sex ratio, % older population, health access, household income, latitude)	Pooled excess mortality risk for cold: 1.04% (95% CI 0.90-1.18%); 3.44% (3.00-3.88%) for heat
Huang et al. 2014	2008-2011	Changsha, China	CVD (I00-I79)	heat, cold	daily mean, max, min temperature	heat: lag 0-3; cold: lag 10-25	1°C decrease below cold temperature threshold (10°C); 1°C increase in temperature above hot temperature threshold (29°C)	age, sex	cold: cumulative effects for CVD mortality 6.6% (95% CI 5.2–8.2%); heat-related CVD mortality: 4.9% (2.0–7.9%)
Ingole et al. 2015	2003-2012	Western India	total	heat, cold	daily Tmax; heat days: defined as days with Tmax >98th percentile (>39°C), cold days: days with Tmax < 2nd percentile (<25°C)	lag 0, lag 0-4	RR of mortality on heat days or cold days compared to other days	age, sex	For those 12–59 yrs on lag 0: RR 1.43 (95% CI 1.02–1.99); among men 1.38 (1.05–1.83); did not observe short-term association between total mortality and cold days
Ingole et al. 2012	Jan. 2003 - May 2010	Vadu, India	total	heat, cold	daily mean temperature	lag 0-1, 2-6, 7-13	1°C increase in temperature	age, sex	strong associations with both high and low temperature for all age groups
Ingole et al. 2017	2004-2013	Vadu HDSS, western India	total	heat, cold	mean temperature	heat: lag 0-1; cold: lag 0-13	1°C increase in daily mean temperature above a threshold of 31 °C	age, sex, occupational classes, ownership of agriculture land, type of house, education	In summer, ORs higher among females (OR 1.93; 95%CI 1.07–3.47), those with low education (1.65; 1.00–2.75), those owing larger agricultural land (2.18; 0.99–4.79), and farmers (1.70; 1.02– 2.81). In winter, high risk of cold-related mortality was observed among people in housework (OR 1.09; CI 1.00–1.19).
Kan et al. 2007	2001-2004	Shanghai, China	non-accidental (<800, A00-R99); CVD (390-459, I00-I99); respiratory (460-519, J00-J98)	heat	DTR	lag 0 to lag 5; lag 0-5	1°C increment	sex, age	1.37% (95% CI 1.08–1.65%), 1.86% (1.40–2.32%) and 1.29% (0.49–2.09%) in total non-accidental, CVD, and respiratory, respectively

Kim et al. 2015	1995-2011	Seoul, South Korea	all-cause (A00-R99, 1-799); CVD (I20–52, 410–429); cerebrovascular (I60–I69, 430–438); respiratory (J00–J99, 460–519)	heat, cold	daily mean temperature	heat: lag 0; cold: lag 4; heat, cold: lag 0-21	heat: 99th percentile relative to 90th percentile (29°C vs. 25°C); cold: 10th vs. 25th percentile (−1°C vs. 4°C)	age	Decreasing trend of heat effect on concurrent days whereas the risk of CVD deaths increased over time; Cumulative risks of deaths increased recently except for respiratory disease
Kim et al. 2011	June-Sep., 2001-2008	Seoul and Daegu, South Korea	all-cause (A00-U99); CVD (I00-I99); respiratory (J00-J99)	heat	mean, min, and max temperature; mean, min, and max T_{app}; and mean, min, and max perceived temperature (PT)	lag 0 to lag 3	1°C increase in temperature	age	For all-cause mortality: Seoul: 2.99% (95% CI 2.43-3.54%) for Tmax; Daegu: 3.52% (2.23-4.80%) for Tmin
Kim et al. 2006	summers for 1994-2003	6 major cities, Korea	non-accidental	heat	daily mean temperature and heat index	lag 0, lag 1	1°C increase above threshold	age	For daily mean temperature increase of 1°C above the thresholds in Seoul, Daegu, Incheon, and Gwangju, 16.3% (95% CI 14.2–18.4%), 9.10% (5.12–13.2%), 7.01% (4.42–9.66%), and 6.7%3 (2.47–11.2%) mortality increases, respectively.
Kim et al. 2017	May to Sep., 2008-2012	Tokyo, Japan	all-cause; CVD; respiratory	heat	daily mean temperature	lag 0 to lag 10	95th and 99th percentile of daily mean temperature compared with 50th percentile of temperature	age (all ages, 65+ yrs), reduced electricity consumption	A 5–9% reduction in all-cause heat-related mortality after the earthquake in the 15 prefectures with the greatest reduction in electricity consumption, and little change in other prefectures. The % reduction in observed vs. expected daily electricity consumption after the earthquake did not significantly modify daily heat-related mortality in Tokyo.
Kim and Joh 2006	2000-2002	Seoul, South Korea	total (exclude V01–Y89)	heat	daily max, mean and min temperature	lag 0 to lag 3	1°C increase in temperature	age, income	High temperature associated with daily mortality in Seoul; association could be higher in low-income group
Li et al. 2015	2003-2011	Guangzhou, China	non-accidental (A00-R99); CVD (I00-I99); respiratory (J00-J99)	heat, cold	mean temperature	lag 0-20	cold: comparing 1st percentile relative to 10th percentile; heat: comparing 99th to 90th percentiles	age, sex, PM_{10} quartiles	cold and heat effects increased with quartiles of PM_{10}

Li et al. 2014	Harbin (2008–10); Nanjing (2004–10); Shenzhen (2004–10); Chongqing (2011–12)	4 cities in China	all-cause (A00-Z99); CVD (I00-I99); respiratory (J00-J99)	heat	daily Tmax	31-day moving avg, including 15 days before and after index day	1°C increase in daily Tmax over threshold	sex, age	strong associations for all-cause and CVD mortality observed in different geographical cities, with increases of 3.2-5.5% and 4.6-7.5%, respectively
Li et al. 2017	May to Sep., 2007-2013	Jinan, China	non-accidental (A00-R99); total CVD (I00-I99); respiratory (J00-J99)	heat	max, min, mean temperature	not specified	1 °C increase above the threshold of Tmax/Tmean/Tmin	sex, age, education level	For non-accidental deaths, effects were significant in individuals aged ≥65 yrs (RR 1.038, 95% CI: 1.026–1.050), but not for those ≤64 yrs; For most outcomes, women and people ≥65 yrs were more vulnerable.
Lim et al. 2015	1991-2010	Japan	all-cause	heat, cold	apparent temperature (T_{app}); temperature deviation index (TDI)	lag 0	per 1-unit (around 1 standard deviation [SD]) and 1°C increase in the TDI and T_{app}; at T_{app} distribution (0–24th, 25–49th, 50–74th, 75–94th, and ≥95th percentile of AT)	latitude	national avg of TDI effects: 0.5% (95% CI 0.1, 1.0%) for the elderly; on summer days with moderate temperature (25th-49th percentile, mean temperature 22.9°C): 1.9 % (1.1-2.6) for the elderly
Lindeboom et al. 2012	1983-2009	Matlab, Bangladesh	all cause	heat, cold	daily min, max, and mean temperature	lag 0-21	every 1°C decrease/increase in mean temp below/above thresholds	sex, age	strongest association for elderly (5.4% increase in mortality with every 1°C decrease at temperatures below 23°C), and opposite trend in ages 1-4 and 5-19 yrs.
Liu et al. 2011	Jan. 2003-Aug. 2005	Beijing, China	CVD (I00-I99); respiratory (J00-J99); cardiorespiratory (I00-J99)	heat	daily mean temperature, T_{app}	lag 0-1, lag 0-14	5°C increase of lag 0-1 avg temperature, 5°C increase of lag 0-14 avg temperature, 5°C increase in temperature above threshold	age	warm period: 5°C increase of lag 0-1 avg temp 1.098 (95% CI 1.057-1.140) for CVD, 1.134 (1.050-1.224) for respiratory; 5°C decrease of lag 0-14 avg temperature 1.040 (95% CI 0.990-1.093) for CVD; cold period: 5°C increase of lag 0-1 avg temperature 1.149 (1.078-1.224) for respiratory ; 5°C decrease of lag 0-14 avg temperature 1.057 (1.022-1.094) for CVD

Luo et al. 2017	2008-2011	3 Chinese cities (Beijing, Nanjing, Chengdu)	CVD (I00-I99)	heat	Temperature variability (TV; SD of daily max and min temperatures over exposure days	lag 0-1 to lag 0-8	every 1◦C increase in TV	3-level air pollutants stratum (low, middle, high), age (<65, 65+ yrs), gender	TV associated with increased risk of CVD mortality, especially for longer TV exposure days (0-8 days). Stronger estimates were observed in females, but no significant difference between males and females was detected. Lack of evidence of effect modification by age and season. Significant effect modification identified for PM_{10}, but not NO_2 or SO_2.
Ma et al. 2014	1996-2008	17 large Chinese cities	non-accidental (A00-R99); CVD (I00-I99); respiratory (J00-J98)	heat, cold	mean temperature	lags 0–14	cold effect: 1°C decrease from 25th to 1st percentiles of temperature; heat effects: 1°C increase from 75th to 99th percentiles of temperature	AC , proportion of green space, proportion of the elderly (≥65 yrs), and disposable income per capita	cold: 1°C decrease from 25th to 1st percentiles, 1.69% (95% PI 1.01-2.36%), 2.49% (1.53-3.46%), and 1.60% (0.32-2.87%) in total, CVD, and respiratory, respectively; heat: 1°C increase from 75th to 99th percentiles, 2.83% (1.42-4.24%), 3.02% (1.33-4.71%), and 4.64% (1.96-7.31%).
Ma et al. 2012	2001-2004	Shanghai, China	total non-accidental	heat	daily avg temperature	lag 0-1	1°C increase in temperature above 18°C	sex, age, education	Elderly subjects more vulnerable; no significant modifying effect of gender or education level
Onozuka and Hagihara 2015	1973-2012	Fukuoka, Japan	all cause (A00-U99, <800)	heat and cold extremes	heat extremes: days with 2-day moving avg of mean temperature >98th percentile (30.0°C) for the entire period; cold extremes: days with 26-day moving avg of mean temperature < 2nd percentile (5.0°C)	heat extremes: lag 0-1; cold extremes: lag 0-25	risk during extreme temperatures compared with non-extreme temperatures	decade, sex, age	RR increased during heat extremes in all decades, with declining trend over time. Mortality risk higher during cold extremes for the entire study period, with dispersed pattern across decades. Meta-analysis showed that heat and cold extremes increased mortality risk.
Qiu et al. 2016	Nov. to Apr., 2002-2011	Hong Kong	circulatory (I00-I99); respiratory (J00-J99)	cold	Extreme cold: days with preceding week with a daily Tmax ≤ 1st percentile of its distribution	lag 0-6	ratio of RR associated with extreme cold in persons who have the condition (e.g., being female) relative to that ratio in other persons	gender, marital status, employment, age (<65, 65-74, 75-84, 85+ yrs)	Subjects age ≥85 yrs more vulnerable to extreme cold, with OR 1.33 (95% CI, 1.22–1.45).

Seposo et al. 2015	2006-2010	Manila City, Philippines	all-cause; CVD (I00–I99); respiratory (J00-J99)	heat	avg temperature	lag 0 for most models	per 1°C increase; mortality in 1st, 5th, 95th, and 99th temperature percentiles and respective MMTs	sex, age, season	for all-cause mortality, increased risk among the elderly (RR 1.53, 95% CI 1.31–1.80), women (1.47, 1.27–1.69)
Sharafkhani et al. 2017	hot season (May to Oct.) and cold season (from Nov. to April of the next year)	Urmia, Iran	Non-accidental (A00-R99); respiratory (J00-J99); CVD (I00-I99)	heat, cold	DTR	lag 0, 0-2, 0-6, 0-13, 0-20, 0-27	mortality risk at low levels (≤ 10th percentile) and high levels of DTR (≥90th percentile) relative to the mortality risk at the 50th percentile of the DTR index	age (below 65, 65-74, 75+), gender	In high DTR values (all percentiles), cumulative RR (CRR) of non-accidental death, respiratory death and CVD death increased in the full year and hot season, and especially in lag 0-6 of the hot season; In the cold season and high DTR values, the CRR of non-accidental death and CVD death decreased, but the CRR of respiratory death increased. Although there was no clear significant effect in low DTR values, high values of DTR increased mortality risk, especially in the heat season.
Son et al. 2011	2000-2007	Seoul, South Korea	all-cause (A00–R99); CVD (I00–I99); respiratory (J00–J99)	heat, cold	daily mean temperature	heat: lag 0; cold: lag 0–25	heat: 90th percentile relative to 50th percentile (25°C vs. 15 °C) and 99th vs. 90th percentile (29°C vs. 25°C); cold: 10th vs. 50th percentile (−1°C vs. 15°C) and 1st vs. 10th percentiles (−4°C vs. −1°C)	sex, age, education, place of death	90th relative to 50th percentiles (25°C vs. 15 °C): 10.2% (95% CI 7.43-13.0%); 10th vs. 50th percentiles (-1°C vs. 15°C): 12.2% (3.6-21.3%)
Son et al. 2016b	2000-2009	Seoul, South Korea	total (A00-R99)	heat	24-h avg	lag 0-1	% change for a 1°C increase in temperature above the threshold (90th percentile or 25.1°C)	urban vegetation, sex, age	The association between total mortality and a 1°C increase in temperature > 90th percentile (25.1°C) (heat effect) was the highest for gus with low NDVI; The heat effect was a 4.1% (95% CI 2.3, 5.9%), 3.0% (95% CI 0.2, 5.9%), and 2.2% (95% CI -0.5, 5.0%) increase in mortality risk for low, medium, and high NDVI group, respectively. Estimated risks were similar by sex and age.

Sun et al. 2016	enroll 1998-2001, 10-13 yrs follow up	Hong Kong	natural (A00-R99, 1-799)	heat, cold	daily mean temperature	lag 0-1, 0-3, 0-13, 0-21	extreme cold: RRs associated with a decrease of temperature from 25th percentile (19.5°C) to 1st percentile (11.3°C); moderate cold: RRs associated with a decrease of temperature from 25th (19.5°C) to 10th percentile (16.2°C); extreme heat (EH): RRs associated with an increase of temperature from 75th (27.8°C) to 99th percentile (30.4°C); moderate heat: from 75th (27.8°C) to 90th percentile (29.4°C)	pre-existing medical conditions	The RR of all natural mortality for extreme cold over 0-21 lag days for those with active disease at the baseline was 2.21 (95% CI: 1.19, 4.10) for diabetes mellitus (DM), 1.59 (1.12, 2.26) for circulatory system diseases (CSD), and 1.23 (0.53, 2.84) for COPD, whereas 1.04 (0.59, 1.85) for non-disease group (NDG). Compared with NDG, elders with COPD had excess risk of mortality associated with thermal stress attributable to hot temperature, while elders with DM and CSD were vulnerable to both hot and cold temperatures.
Sung et al. 2013	1994-2008	6 major cities, Taiwan	natural cause (A00-R99, 001-799)	heat	daily mean heat index	lag 0 to 4	RRs by daily mean heat index categories (percentiles) compared with the reference (0–4th percentiles)	age	Overall, daily mean heat indices associated with increased risk ratios in mortality. Significantly increased risk ratios of daily mortality evident when daily mean heat indices ≥95th percentile, compared to lowest percentile, in all cities.
Wang et al. 2015	2007-2009	Beijing and Shanghai, China	CVD (I00-I69)	heat, cold	mean temperature	cold: lag 0-27; heat: lag 0-14	cold: 1st percentile relative to 10th percentile (-6.1°C vs. -1.5°C for Beijing, 0.5°C vs. 5.3°C for Shanghai); heat: 99th to 90th percentiles (29.8°C vs. 26.9°C for Beijing, 32.4°C vs. 28.9°C for Shanghai)	age	For CVD, Beijing had stronger cold and heat effects than Shanghai; cold effects on CVD mortality strongest at lag0–27, while heat effects strongest at lag 0–14.
Wang et al. 2014	2005-2008	Suzhou, China	total non-accidental (A00-R99); CVD (I00–I99); respiratory (J00–J99)	heat, cold	mean temperature	lag 0–3, 0–7, 0–21, 0–28	extreme cold (1st percentile) and EH (99th percentile) temperatures with the MMT	sex, age, educational attainment	extreme cold lag 0–14: 1.75 (95% CI 1.43-2.14); EH lag 0–3: 1.43 (1.31-1.56)

Wang et al. 2017	2007-2012	122 communities, China	non-accidental (A00-R99)	heat, cold	mean temperature	lag 0-3, 0-7, 0-15, 0-21	RR below/above the MMT	temperature zones (sub temperate, warm temperate, north subtropical, middle subtropical, south subtropical)	At the community level, the mean value of relative extreme cold risk (1.63) of all 122 communities was higher than that of extreme high temperature (1.15). At regional level, temperature-mortality relationship varied in different temperature zones. An M-shaped curve was estimated for the relationship between cold risk and temperature, with an inverted U-shaped with a right tail for the heat effect.
Wu et al. 2013	Changsha and Kunming 2006–2009; Guangzhou and Zhuhai 2006–2010	4 subtropical cities, China	all-cause (A00-R99)	heat, cold	daily mean temperature	lag 0, 0-6, 0-13, 0-20, 0-27	1°C decrease/increase of temperature under/above the cold/heat threshold	age	Total mortality: For cold: RR 1.061 (95% CI 1.023–1.099), 1.044 (1.033–1.056), 1.096 (1.075–1.117) and 1.111 (1.078–1.145) for Changsha, Kunming, Guangzhou. and Zhuhai, respectively; for heat: 1.020 (1.003–1.037), 1.017 (1.004–1.030), 1.029 (1.020–1.039) and 1.023 (1.004–1.042), respectively.
Xu et al. 2013	1998-2009	Hong Kong	all-cause; CVD (390-459, I00-I99); respiratory (460-519, J00-J99)	cold	daily mean T_{app}	lag 0 to lag 6, lag 0-1 to lag 0-6, lag 7-13	1°C decrease in daily mean T_{app} <20.8°C	sex, BMI	1.99% (95% CI 0.64-2.64%) for all-causes; 2.48% (0.57-4.36%) for CVD; 3.19% (0.59-5.73%) for respiratory
Yang et al. 2015	2007-2013	15 Chinese megacities	CVD (I00-I99)	heat, cold	mean daily temperature	lag 0-21	% increase per unit increase below and above MMTs	gender, age, education level (illiterate, primary education, and high school and above)	CVD: 17.1% (95% CI 14.4-19.1%) with substantial differences among cities; More attributable deaths due to cold, [15.8% (13.1-17.9%)], vs. heat [1.3% (1.0-1.6%)].
Yang et al. 2012	2003-2007	Guangzhou, China	non-accidental (A00-R99); CVD (I00-I99); respiratory (J00-J99)	heat, cold	daily mean temperature	heat: lag 0-5; cold: lag 0-12	cold: 1st percentile relative to 10th percentile; heat: 99th vs. the 90th percentile	sex, age, educational attainment and occupation class	cold: 20.39% (95% CI 11.78%, 29.01%); heat: 15.46% (10.05%, 20.87%) increase
Yi and Chan 2015	2002–2011	Hong Kong	non-accidental (A00-R99); cardiopulmonary (I00-I99, J00-J99); CVD (I00-I99); respiratory (J00-J99)	heat, cold	max, mean, and min temperature	cold: lag 0-13; heat: lag 0-3	cold: 1st percentile relative to 25th percentile (11.1°C vs. 19.4°C); heat: 99th to 75th percentile (31.5°C vs. 27.8°C)	age	non-accidental mortality, cold: 1.17 (95 % CI 1.04, 1.29) for lag 0-13; heat: 1.09 (1.03, 1.17) for lag 0-3

Yin and Wang 2017	June to Aug., 2010 to 2012	Beijing, China	CVD (I00-I99)	heat	Tmax	not specified	MMT (30.5 °C) as the reference temperature and estimated the excess mortality risks of different temperature thresholds (32 °C, 33 °C, 34 °C, and 35 °C) and durations (1–11 days)	age (<65, 65+ yrs), occupation (indoor workers, outdoor workers, unemployed, and unknown)	extreme high temperatures appeared to contribute to a higher proportion of CVD deaths among elderly persons, females and outdoor workers.
Zeng et al. 2017	2010–2013	11 cities in Zhejiang Province, China	CVD (I00-I99)	heat, cold	mean temperature	lag 0-3, 0-7, 0-14, 0-21	RR below/above the MMT; daily attributable death corresponding to each day's temperature; attributable fraction (AF)	sex, age group (0-74, 75+ yrs), education level (0-6, 7+ yrs), location of cities (coastal and inland), and humidity (low and high of its median value)	The AFs were significant at low temperature and high humidity for males, youth, those low education, and persons in coastal areas.
Zhang et al. 2014	Beijing 2007-2008, Shanghai 2004-2008, Guangzhou 2001-2008	3 largest cities, China	non-accidental	heat, cold	daily max, avg, min temperature	lag 0, 1-3, 4-6, 7-9, 10-12, 13-15, 16-18, 19-21, 22-24, 25-27, 28-30	cold: 1°C decrease below cold threshold; heat: 1°C increase above heat threshold	age	J-shaped association between temperature and mortality, with increased mortality risk of heat and cold in all cities
Zhang et al. 2017a	2009-2012	Hubei, China	non-accidental (A00-R99); cardiorespiratory (I00-I99 and J00-J99); CVD (I00-I99); respiratory (J00-J99)	heat	TV; SD of max and min temperatures during the exposure days	lag 0-7	per 1°C increase in TV	gender, age (0-74, 75+ yrs), education attainment (0-6, 7 yrs), place of death (in hospital, outside the hospital), urban/rural	The elderly were more susceptible to TV-related mortality effects than younger age groups. Some slight differences in effect estimates were observed by gender, education, place of death, and urban/rural areas.
Zhang et al. 2017b	2009-2012	Wuhan, central China	non-accidental (A00-R99); respiratory (J00-J99); CVD (I00-I99); cardiorespiratory (I00-I99 and J00-J99)	heat	DTR	lag 0-1	1°C increase in DTR	gender, age (0–74, 75+ yrs), and education level (0–6, 7+ yrs)	For the whole population, a 1°C increase in DTR at lag 0-1 was associated with an increase of 0.65% (95% CI: 0.08–1.23) for mortality. Relatively stronger DTR-mortality was found for CVD deaths. Females, those 75+ yrs, and those with more education (7+ yrs) suffered more significantly from increased mortality due to large DTR.
**North America**									
Anderson and Bell 2009	1987-2000	107 US communities	CVD (390-448); respiratory (480-486, 490-497, or 507)	heat, cold	daily mean, max, and min temperature, and mean T_{app}	heat: lag 0-1; cold: lag 0-25	cold: 1st percentile relative to 10th percentile, 4.4°C vs. 15.6°C; heat: 99th vs. 90th percentile, 26.7°C vs. 15.6°C	age, socioeconomic conditions, urbanicity, and central AC	Heat: 3.0% (95% PI 2.4%–3.6%) comparing community's 99th and 90th temperature percentiles; cold: 4.2% (3.2%–5.3%) comparing 1st and 10th percentiles

Basu et al. 2015	May-Oct., 1999-2011	California, US	all-cause; respiratory (J00-J98, U04); circulatory (I00-I99)	heat	mean daily T_{app}	lag 0 to 6, 0-1, 0-3, 0-6	per 5.6°C increase for avg of same day and previous 3 days T_{app}	black infants/white infants, costal and non-coastal regions	all-cause mortality: 4.4% (95% CI −0.3, 9.2) per 5.6°C increase for lag 0-3
Basu and Malig 2011	warm season for 1999-2006	13 counties in California	non-accidental (A00-U99); CVD (I00-I99); respiratory (J00-J98)	heat	daily mean T_{app}	lag 0 to 20, 0-4, 0-9	per 5.6°C increase in T_{app}	age, sex	excess risk 4.3% (95% CI 3.4, 5.2) per 5.6°C increase in T_{app}, for non-accidental mortality
Braga et al. 2002	1986-1993	12 U.S. cities	CVD (390–429)	heat, cold	daily mean temperature	lag 4–6, 3, 4	% increase at 30°C and at –10°C for difference between the 90th and 10th percentiles in AC, variance of summer temperature, and variance of winter temperature	hot cities/cold cities	In cold cities, high and low temperatures associated with CVD. For CVD, hot day effect 5 times smaller than cold day effect. In hot cities, neither hot nor cold temperatures associated with CVD deaths.
Cagle and Hubbard 2005	1980-2001	King County, Washington, US	cardiac-related (390–398.9, 402.*, 404.*, 410–419, 420–429, I00–I11.9, I13.0–I13.9, I20.0–I59.9)	heat	daily avg temperature	lag 0 to 5	5˚C increase	sex, season	significant negative association between daily avg temperature and cardiac mortality for those >55 yrs; 5˚C increase in temperature associated with decreased mortality rate by factor of 0.971 (95% CI 0.961-0.982).
Chen et al. 2017	1990-2011	12 Texas Metropolitan Areas, US	all-cause; CVD (390-429, I01-I52); respiratory (460-519, J00-J99)	cold	mean temperature	lag 0-25	1 °C decrease below the threshold	age (0-64, 65-74, 75+ yrs)	Higher mortality generally observed in MSAs with higher average daily mean temperatures and lower latitudes. Pooled effect estimate was 1.58% (95% CI [0.81, 2.37]) increase in all-cause mortality risk with a 1 °C decrease in temperature. Effects of cold on all-cause mortality were highest among people ≥75 yrs (1.86%, 95% CI 1.09, 2.63).
Curriero et al. 2002	1973–1994	11 large eastern US cities	CVD (390–459); respiratory (460–519)	heat, cold	avg temperature	lag 0, 1-3, 4-10	per 10°F per 10-unit change in effect modifier; avg slope of estimated RR curves at temperatures lower/higher than MMT	latitude, city-specific characteristics such as % of elderly persons and % of homes with heating and/or air conditioning	weather components most strongly predictive of mortality: current and recent days' temperatures. Mortality risk generally decreased as temperature increased from coldest days to a threshold temperature, which varied by latitude, above which mortality risk increased as temperature increased.

Goldberg et al. 2011	1984-2007	Montreal, Canada	non-accidental; cardio-respiratory	heat, cold	Tmax	30 days	1st percentile relative to 10th percentile, 99th vs. 75th percentile, 99th vs. 90th percentile	sex, age	across all lags daily non-accidental mortality increased 28.4% (95% CI 13.8–44.9%) when temperatures 22.5 to 31.8˚C (75th to 99th percentiles)
Gronlund et al. 2015	May-Sep., 1990-2007	8 Michigan cities, US	natural (<800, 992, E900.0, A-R, T67, X30); CVD (390-429, I0-I52); respiratory (460-466, 480-487, 490-492, 494-496, J9-J18, J40-J44, J47)	heat	EH (indicator for 4-day mean, min, max or T_{app} above 97th or 99th percentiles)	lag 0-1, 2-3, 4-5, 6-7	mortality during EH vs. non-EH	personal marital status, age, race, sex, education, and ZIP-code % non-green space, income, living alone, and housing age	EH vs. non-EH, for CVD: higher among non-married individuals (OR 1.21, 95% CI 1.14-1.28 vs. 0.98, 95% CI 0.90-1.07 among married individuals) and individuals in ZIP codes with high (91%) non-green space (1.17, 95% CI 1.06-1.29 vs. 0.98, 95% CI 0.89-1.07 for ZIP codes with low (39%) non-green space.
Harlan et al. 2014	May–Oct., 2000-2008	central Arizona desert cities, US	all-cause (excluding S00-99, T00-66, T68-98, U00-99, V01-99, W00-99, X00-29, 31, 33-53, 55-84, Y00-98, Z00-99 and including T67.x, X30, X32 and X54)	heat	daily max T_{app}	lag 0, 1, 2, 3	RR above threshold per 1°F	age, sex	The most robust relationship was between max T_{app} on day of death and mortality from direct exposure to high environmental heat; the heat thresholds in all gender and age groups (max T_{app} 90–97 °F; 32.2–36.1 °C) were below local median seasonal temperatures in the study period (max T_{app} 99.5 °F; 37.5 °C).
Ho et al. 2017	1998-2014	Vancouver, Canada	all deaths excluding accidents (V01-V99)	heat	daily mean humidex	not specified	1°C increase in daily mean humidex at Vancouver International Airport (YVR)	The Vancouver Area Neighborhood Deprivation Index (VANDIX) based on % of population that did not finish high school, unemployment rate, % of population with university education, % of single-parent families, average income, % of homes owned, and labor participation rate (% of adult population that is either employed or actively looking for work)	The heat exposure and social vulnerability variables with the strongest spatially stratified results were the apparent temperature and the labor nonparticipation rate. Areas at higher risk had values ≥ 34.4°C for the max T_{app} and ≥ 60% of the population neither employed nor looking for work. These variables were combined in a composite index to quantify their interaction and to enhance visualization of high-risk areas.

Isaksen et al. 2016	May-Sep., 1980-2010	King County, Washington, US	non-traumatic (001-799, A01-R99); all-cause (000+, A00+); circulatory (390-459, I00-I99, G45, G46); CVD (393-429, I05-I52); respiratory (460-519, J00-J99)	heat	humidex (daily Tmax and avg RH values)	lag 0	99th percentile (36.1°C) heat day compared with non-heat day; per degree increase in county-wide avg daily max humidex (°C) above 36.0°C	age (0–4, 5–14, 15–44, 45–64, 65–84, 85+), gender, race, high school graduation, marital status, Hispanic origin, and tobacco use; type of synoptic weather (ambient weather conditions) on a given heat day	For all ages, all-causes, a 10% (1.10, 95% CI 1.06-1.14) increase on a heat day vs. non-heat day. When considering the intensity effect of heat on all-cause mortality, a 1.69 % (0.69-2.70) increase per unit of humidex >36.0°C. All-cause mortality was modified by synoptic weather type. Age was the only individual-level characteristic found to modify mortality risks.
Rosenthal et al. 2014	1997-2006	New York City, US	natural	heat	heat index	lag 0	comparing death rate (per days) on extremely hot days (max heat index >100 °F) to death rate on all days in the warm season (May-Sep.)	place-based characteristics (socioeconomic/demographic and health factors, as well as the built and biophysical environment)	Significant positive associations between mortality rate ratio among those ≥65 yrs and neighborhood-level characteristics: poverty, poor housing, lower access to AC, impervious land cover, surface temperatures, and seniors' hypertension. % Black/African American and household poverty were strong negative predictors of seniors' AC access.
Lee et al. 2016	2007-2011	Georgia, North Carolina, and South Carolina, US	non-accidental	heat, cold	modeled daily mean temperature	not specified	1 °C decrease/increase in temperature below −1 °C/above 28 °C	age group, sex, race, residence, and education	Children <15 yrs had the largest % increase per 1 °C increase in temperature (8.19%, 95% CI -0.38 to 17.49%) followed by Blacks (4.35%, 95% CI 2.22 to 6.53%). Higher education was protective for the effect of extreme temperature. Results suggest that people in less urban areas were more susceptible to extreme temperature. The association between temperature and mortality was stronger when using exposure data with more spatial variability than using exposure data based on existing monitors alone.

Madrigano et al. 2015b	1988-1999	counties in northeastern US	non-accidental	heat	daily Tmax	lag 0-1	increase in mortality comparing 90°F to 70°F	population density, urban/non-urban, County characteristics, such as population density, % of families living in poverty, and % of elderly residents	8.88% increase in mortality (95% PI 7.38, 10.41) in urban counties, 8.08% increase (95% PI 6.16, 10.05) in nonurban counties
Medina-Ramon et al. 2006	1989–2000	50 US cities	CVD (390–429, I01–I51)	heat	daily Tmin and Tmax in each city to define extremely hot days (with a daily Tmin ≥ 99th percentile) and extremely cold days (with daily Tmax ≤ 1st percentile)	lag 0, 0-1, 1, 2, 0-2	relative odds comparing extreme temperature day for persons with the condition (e.g., being female) compared with persons without the condition	age, sex, race, education, place of death, chronic condition	Older subjects (OR 1.020; 95% CI 1.005–1.034), blacks (1.037, 1.016–1.059), and those dying outside a hospital (1.066, 1.036–1.098) more susceptible to EH, with some differences between those dying from CVD and other causes. CVD (1.053, 1.036–1.070) had higher relative increase on extremely cold days, whereas increase in heat-related mortality was marginally higher for those with coexisting atrial fibrillation (1.059, 0.996–1.125).
Medina-Ramon and Schwartz 2007	1989-2000	50 US cities	all-cause	heat	1) extremely cold days: daily Tmax ≤1st percentile, extremely hot days: daily Tmin ≥99th percentile; 2) cold: value 0 when daily Tmax ≥17°C and then increased with increasing cold, heat: value 0 when the daily Tmin ≤17°C and increased with increasing hot temperature	2-day cumulative risk estimate by summing the estimates for lag 0 and lag 1	% change in mortality on extreme temperature days relative to other days; % change in mortality per each degree of max daily temperature < 17°C for heat exposure, % change in mortality per each degree of min daily temperature > 17°C	several city characteristics as effect modifiers	Mortality increases associated with extreme cold (2-day cumulative increase 1.59%, 95% CI 0.56-2.63%) and EH (5.74%, 3.38-8.15%) were found.
Metzger et al. 2010	May-Sep., 1997-2006	New York City, US	natural-cause (A00–R99, 001–799)	heat	max heat index; min, max, and avg (mean of min and max) temperature; spatial synoptic classification (SSC) of weather type	lag 0 to lag 3	unit increase	effect modification of temperature by time of year (interaction terms with day of year and month)	A model with cubic functions of max heat index on lag 0 to lag 3 provided the best fit, compared to models using max, min or avg temperature, or SSC of weather type.

Nordio et al. 2015	1962-2006	211 US cities	all-cause (A00-U99, 1-799)	heat, cold	daily mean temperature	heat: lag 0; cold: moving avg lag 1-5	heat: risk at 26.7°C (80 °F) relative to 15.6°C (60 °F); cold: risk at 4.4°C (40 °F) vs. 15.6°C (60 °F); heat: compare 99th to 50th percentiles; cold: compare 1st to 50th percentiles	AC	Effect of hot days diminished with increasing summer mean temperature within city; effect of cold days increased with increasing winter mean temperature
O'Neill et al. 2005b	1986-1993	4 US cities	non-injury mortality	heat	mean daily T_{app}	lag 0, lag 1-3	% change in mortality at 29°C, relative to 15°C	AC prevalence, race	Heat-related mortality reduced with increasing central AC prevalence; substantially higher heat effects among Blacks compared with Whites
Petkova et al. 2014	1900-1948 and 1973-2006	New York, NY, US	total	heat	mean daily temperature	lag of 5 days for the main model	RR at 29°C vs. 22°C	age, decade-specific estimates	decade-specific RR ranged from 1.30 (95% CI 1.25-1.36) in 1910s to 1.43 (95% CI 1.37-1.49) in 1900s. Since 1970s, gradual and substantial decline in RR, from 1.26 (95% CI 1.22-1.29) in 1970s to 1.09 (95% CI 1.05-1.12) in 2000s.
Rainham and Smoyer-Tomic 2003	1980–1996	Toronto, Canada	non-accidental (<800); combined cardiac and respiratory (390–459, 480–519)	heat	daily humidex using daily max dry-bulb temperature and dewpoint temperature that occurred at same hour as max air temperature	lag 0, lag 1, lag 0-1	both 1°C and 50–95th percentile increases in humidex	age group, sex	RR narrowly exceeded 1.0 for all groups, with adjustment for air pollution. Humidex effects most apparent for females (RR 1.006, 95% CI 1.004–1.008 per 1°C humidex and RR 1.089 (1.058–1.121) for 50th to 95th percentiles humidex). Without air pollution adjustment, RR in the 50–95th percentile increased less than 1.71% for all groups except females, for which RR decreased 1.42%. Differences in RR per 1°C humidex were <0.12%.
Ren et al. 2008	June-Sep., 1987-2000	95 large US communities	CVD (390-448, I000-I799)	heat	daily Tmax	lag 0, lag 1	per 10°C increase in Tmax	region, ozone level	Increase in CVD mortality by 1.17% and 8.31% for areas with lowest and highest ozone levels in all communities, respectively
Smargiassi et al. 2009	summers, 1990–2003	Montreal, Canada	non-accidental (excluding 800–999 and S00–T98); CVD (360–459, I00–I99); respiratory (460–519, J00–J99)	heat	daily mean temperature	lag 0, 1, 0-1	increments of 2°C in daily mean temperature	residential dwelling values (proxy for the socioeconomic status) and categories of surface temperatures at place of death	Risk of death on warm summer days in areas with higher surface temperatures higher than in areas with lower surface temperatures

Xiao et al. 2015	1987–2000	13 eastern US cities	non-external (<800, A00-R99)	heat, cold	daily mean, max, and min temperature	lag 0, 0–6, 0–13, 0–20, 0–27, 0–34; cold: lag 0–27; heat: lag 0–6	per 1°C decrease or increase; per interval increase in a city-specific characteristic	city-level characteristics (such as the % of population >65 yrs, poverty of individuals, education level <9th grade, and latitude of city)	latitude modified cold and heat effects (statistically significant). Cold effect decreased (−0.11 % change of mortality effect f)or 1° latitude increment, while heat effect increased 0.18 % 1° latitude increment.
Zanobetti and Schwartz 2008	May-Sep., 1999 to 2002	9 US cities	all-cause excluding accidental causes (V01-Y98, 1-799)	heat	T_{app}	lag 0, 0-3, 1-3	5.5°C increase in T_{app}	air pollution, various temperature definitions	1.8% (95% CI 1.09-2.5%) using case-crossover analysis, 2.7% (2.0-3.5%) using time-series
Zhan et al. 2017	1987-2000	106 communities, US	non-accidental; CVD; respiratory	heat, cold	T_{app}; temperature change between neighboring days: subtracting the previous day's mean temperature from the current day's mean temperature	lag 0-21	A temperature change between neighboring days (TCN) of 0°C was used as a reference for calculating RRs; 1st and 99th percentiles of daily TCN distribution as extremely negative and positive TCNs	age (<75, 75+ yrs), geographic regions	At national level, a monotonic increasing curve of TCN-mortality association was observed, which indicated that negative TCN was associated with reduced mortality and positive TCN elevated mortality risk. RR for lag 0-21 was 0.63 (95% CI 0.59-0.68) for extremely negative TCN (1st percentile) and 1.46 (1.39-1.54) for extremely positive TCN (99th percentile) for non-accidental mortality. Prominent effects of extreme TCNs for CVD and respiratory mortality. People ≥75yrs and those with respiratory disease were identified as particularly vulnerable to TCN. The TCN-mortality association was modified by season and region.
**Europe**									
Almeida et al. 2013	April-Sep., 2000-2004	Lisbon and Oporto, Portugal	all natural (<800, A00-R99); CVD (390–459, I00–I99); respiratory (460–519, J00–J98)	heat	max T_{app}, Tmax	lag 0-3	for every 1°C elevation in max T_{app} above city-specific threshold	age	all-cause mortality rate: 7.13%% (95% CI 5.9-8.4%) in Lisbon and 4.31% (3.2-5.4%) in Oporto
Almeida et al. 2010	Lisbon (April-Sep., 2000-2004), Oporto (2000-2004)	Greater Lisbon and Greater Oporto, Portugal	all-cause (<800, A00-R99); CVD (390-459, I00-I99); respiratory (460-519, J00-J98)	heat	T_{app}	lag 0 to 3, lag 0-3	per 1°C increase in mean daily T_{app}	age	In Lisbon: 2.1% (95% CI 1.6, 2.5), 2.4% (1.7-3.1), and 1.7% (0.1-3.4) for all-causes, CVD, and respiratory, respectively. In Oporto: 1.5% (1.0-1.9), 2.1% (1.3-2.9) and 2.7% (1.2-4.3), respectively.

Antunes et al. 2017	Nov. to March, 1992-2012	Lisbon and Oporto, Portugal	All-causes (A00-Y98, 000-999); circulatory (I00-I99, 390-459) plus respiratory (J00-J99, 460-519)	cold	Tmin	max lag: lag 31	1st vs. 99th percentile of Tmin; 1°C decrease from reference temperature	age (0-64, 65+)	The overall effect was generally higher and more persistent in Lisbon than Oporto, particularly for circulatory and respiratory mortality and for the elderly.
Ballester et al. 1997	1991-1993	Valencia, Spain	total (001–799); circulatory (390–459); respiratory (460–519)	heat, cold	mean daily temperature	lag 0, 1-2, 3-6, 7-14	1°C decrease/increase in daily temperature below 15°C/above 24°C	warm/cold months, age	The effect of temperature greater in persons > 70 yrs, and in cases of circulatory and respiratory diseases.
Breitner et al. 2014a	1990-2006	3 cities of Bavaria, Germany	non-accidental (1-799, A00-R99); CVD (390-459, I00-I99); respiratory (460-519, J00-J99)	heat, cold	mean daily temperature	lag 0-1; lag 0-14	heat: 90th percentile relative to the 99th percentile (20.0°C vs. 24.8°C); cold: 10th vs. 1st percentile (−1.0°C vs. -7.5°C)	sex, age, ambient air pollution	in non-accidental mortality, heat: 11.4% (95% CI 7.6-15.3%) increase; cold: 6.2% (95% CI 1.8–10.8%) increase
Breitner et al. 2014b	1990-2006	Bavaria, Germany	CVD (390–459, I00-I99)	heat, cold	24-h mean values	lag 0-1, lag 0-14	heat: 90th percentile relative to 99th percentile (20.0°C vs. 24.8°C) in 2-day avg temperature; cold: 10th vs. 1st percentile (−1.0°C) vs. −7.5°C) in 15-day avg temperature	Breitner et al. 2014	1990-2006
Burkart et al. 2016	1998-2008	Lisbon, Portugal	total	heat	UTCI	lag 0-2	1°C increase in UTCI above 95th or 99th percentiles	vegetation (urban green), proximity to water (urban blue)	For areas in lowest NDVI quartile (14.7%; 95% CI 1.9-17.5%) for 1°C increase in UTCI above 99th percentile (24.8°C); for areas in highest quartile (3.0%, 2.0-4.0%); In areas > 4 km from water, 1°C UTCI increase >99th percentile associated with 7.1% (6.2-8.1%); for areas ≤ 4 km from water, 2.1% (1.2-3.0%).
Carson et al. 2006	4 periods (1900–1910, 1927–1937, 1954–1964, and 1986–1996)	London, UK	all-cause; CVD respiratory	heat, cold	daily mean temperatures as mean of daily max and min temperatures	mean of lag 0 and 1 week	% increase in deaths per °C above/below 15°C	age	increase in mortality per 1°C decrease below 15°C was 2.52% (95% CI 2.00-3.03), 2.34% (1.72-2.96), 1.64% (1.10-2.19), and 1.17% (0.88-1.45) in the 4 time periods. Heat deaths also diminished over time.

Diaz et al. 2006	1986-1997	Madrid, Spain	non-accidental (1–799); circulatory (390–459); respiratory (460–519)	heat, cold	max, min; heat waves: Tmax > 36.5°C, cold waves: Tmax < 6.0°C	lag 0, 1, 2, 3, 7, 8	for each degree of Tmax above 36.5°C/below 6°C	sex, summer/winter	Mortality association limited to temperatures from 5th to 95th percentiles, and increased sharply thereafter. During summer, heat wave effect detected solely among males 45-64 yrs, with AR of 13.3% for circulatory causes. During winter, the impact of cold exclusively observed among females with AR of 7.7%.
Donaldson and Keatinge 2003	1998-2000	England and Wales	all cause	cold	daily mean temperature	lagged 3 days	% change in mortality/°C	sex, social class in working and retired age groups	Cold related mortality was generally low in unskilled class men of working age (50–59 yrs) only, compared with men in other classes, and unskilled class women or housewives.
Gasparrini et al. 2012	June-Sep., 1993-2006	10 regions in England and Wales	all-cause	heat	Tmax	lag 0-1	1°C increase in temperature above region-specific thresholds	age	all-cause mortality 2.1% (95% CI 1.6-2.6%); steepest increase in risk for respiratory (4.1%, 3.5-4.8%). smaller for CVD (1.8%, 1.2-2.5%).
Goodman et al. 2004	April-Dec., 1980-1996	Dublin, Ireland	total non-trauma deaths (<800); CVD (390–448); respiratory (460–496, 507)	heat, cold	Tmin	heat: lag 0; cold: lag 0-39	each increase/decrease of 1°C	age	1°C increase in lag 0 temperature associated with 0.4% increase in total mortality; each 1°C decrease associated with 2.6% increase in the following 40 days
Gómez-Acebo et al. 2012	2003-2006	Cantabria, Spain	mortality	heat, cold	Tmin, Tmax	lag 0	1°C increase or decrease	sex, age	1°C increase in max or min temperatures associated with 2% excess mortality risk in whole population throughout the warm period
Gómez-Acebo et al. 2010	2004-2005	Cantabria, Spain	total	cold	avg, max, min temperature	lag 0 to lag 6	temperatures below 5th percentile compared with temperatures above 5th percentile	lag, age group, temperature quintile group	Temperatures <5th percentile strongly associated with mortality compared with temperatures >5th percentile (OR 3.40, 95% CI 2.95-3.93 for lag 6).

Hajat et al. 2007	1993-2003	England and Wales	CVD (390.0–459.9, I); respiratory (460.0–519.9, J)	heat, cold	Daily mean temperature as mean of daily max and min temperature; heat threshold derived from 95th percentile of mean temperature, cold threshold from the 5th percentile for each region separately	heat: lag 0-1; cold: lag 0-13	increase 1°C above heat threshold and below cold threshold	region, sex, age, urban or rural, long-term care status (home, nursing home, or none), region-specific quintiles of census and area-level measure of deprivation	strongest heat effects in London, strongest cold effects in the Eastern region. For all regions, heat: mean RR of 1.03 (95% CI 1.02-1.03); cold: 1.06 (1.05-1.06)
Hajat et al. 2016	Oct.-Mar., 1993-2006	England	all-cause; CVD (I00-I99); respiratory (J00-J99); COPD (J40-J44); external (V01-Y99)	cold	daily mean temperature	lag 0-28	every 1°C decrease in temperature below thresholds	age (0-15, 16-64, 65-74, 75-84, 85+ yrs)	Nationally, 3.44% (95% CI: 3.01, 3.87) increase in all-cause deaths for every 1 °C decrease in temperature below identified thresholds; The very elderly and people with COPD were most at risk from low temperatures.
Huynen et al. 2001	1979–1997	Netherlands	respiratory (AM 33–35); CVD (AM 25–32)	heat, cold	avg daily temperature as avg of min and max temperatures	lag 0, 1-2, 3-6, 7-14, 15-30	1ºC increase/decrease above/below the optimum in preceding month	age	For temperatures above optimum, mortality increased 1.86%, 12.82%, and 2.72% for CVD, respiratory, and total mortality, respectively. For temperatures below optimum, mortality increased 1.69, 5.15, and 1.37%, respectively.
Iñiguez et al. 2010	1990-1996	13 Spanish cities	natural (0–799); cardiorespiratory (390–519)	heat, cold	daily mean temperature (avg of lag 0 min and max values)	lag 0, lag 1-3, lag 4-10	1°C increase/decrease in temperature from MMT	cold/mild/warm cities grouping, age	Cold and heat effects depended on climate: effects higher in hotter cities and lower in cities with higher variability. In general, effect of cold and MMT higher for cardiorespiratory than total mortality, while the effect of heat generally higher among the elderly.

Kunst et al. 1993	1979-1987	Netherlands	all-cause, CVD (380-459); respiratory (460-519)	heat, cold	avg temperature; cold: avg temperatures < 16.5°C, warm: > 16.5°C	lag 0, 1-2, 3-6, 7-14, 15-30, aggregate (5 lag periods)	1°C increase below/above 16.5°C	wind speed, humidity	Direct effects of cold and heat on mortality suggested: 1) control for influenza incidence reduced cold-related mortality by 34% and reduced heat-related mortality by 23% (role of air pollution and "season" was negligible); 2) 62% of "unexplained" cold-related mortality, and all heat-related mortality, occurred within 1 week; and 3) effect modification by wind speed was in expected direction.
Mackenbach et al. 1993	1979-1987	Netherlands	all-cause; CVD (AM 25-32); respiratory (AM 33-35)	heat, cold	avg temperature as difference between max and min temperatures; cold: < 16.5°C, warm: >16.5°C	lag 0, 1-5, 6-10, 11-15	1°C increase below/above 16.5°C	sex, age	Low temperatures had strongest lagged effects on mortality. Results similar for other causes of death.
Milojevic et al. 2016	1993-2006	London, UK	all-cause	heat, cold	daily mean temperature	heat: lag 0-1; cold: lag 0-13	RRs for hot and cold days with daily mean temperatures > 22.3°C or < 6.4°C, respectively, compared with days with daily mean temperatures ≥ 6.4 and ≤ 22.3°C	urban heat island decile groups	RR on hot vs. normal days differed little across UHI decile groups. A 1°C UHI anomaly multiplied risk of heat death by 1.004 (95% CI: 0.950, 1.061) compared with expected value of 1.070 (1.057, 1.082) if there was no acclimatization. The corresponding UHI interaction for cold was 1.020 (0.979, 1.063) vs.1.030 (1.026, 1.034) (actual vs. expected under no acclimatization, respectively). Fitted splines for heat shifted little across UHI decile groups, suggesting acclimatization. For cold, splines shifted somewhat in the direction of no acclimatization, but did not exclude acclimatization.
Morabito et al. 2012	1999–2008	Tuscany, Central Italy	non-accidental (<800)	heat, cold	daily avg temperature	lag 0 to lag 30	1°C decrease/increase in temperature below/above threshold	age	cold: 2.27% (95% CI 0.17–4.93%); heat: 15.97% (7.43–24.51%) in coastal plain cities

Oudin et al. 2016	June to Sep, 1997–2013	Estonia	total	heat	Tmax	lag 0-2, lag 0-10	risk above MMT (75th percentile of Tmax)	age (0-74, 75+), gender, region	An immediate increase in mortality associated with temperatures > 75th percentile of summer Tmax, corresponding to ~23°C. The total effect of elevated temperatures was not lessened by significant mortality displacement.
Pattenden et al. 2010	May-Sep., 1993-2003	15 conurbations in England and Wales	all-cause except external causes; CVD (3900-4599, I); respiratory (4600-5199, J)	heat	daily mean temperature	lag 0-1	comparing adjusted mortality rates at 97.5th and 75th percentiles	age	mean mortality rate ratio for heat effect across conurbations: 1.071 (1.050-1.093)
Rabczenko et al. 2016	May - Sep, 2008-2013	Warsaw, Poland	all-cause	heat	Tmax	lag 0-7	RRs associated with temperature increase by 1°C below/above change point temperature (20-24°C)	sex, age	RR associated with increase of temperature above the calculated optimum is equal to 1.6%, being higher in females (2.7%) than in males (1.5%).
Ragettli et al. 2017	1995-2013	8 Swiss cities, Switzerland	non-external deaths (A00-R99, V01-V99, W00-X59)	heat	mean, max, min, max T_{app}	lag 0-6	increases in temperature from the median to the 98th percentile of the warm season temperature distribution	gender, age (0-74, 75+ yrs)	Over the whole time period, significant temperature-mortality relationships were found for all temperature indicators (RR (95% CI): max T_{app}: 1.12 (1.05; 1.18); Tmax: 1.15 (1.08–1.22); Tmean: 1.16 (1.09–1.23); Tmin 1.23 (1.15–1.32)). Mortality risks higher at beginning of summer, especially for Tmin. In the more recent time period, a non-significant reduction in the effect of high temperatures on mortality, with those >74 yrs remaining the population at highest risk.
Revich and Shaposhnikov 2008b	Jan. 2000 - Feb. 2006	Moscow, Russia	all non-accidental (exclude V00-Y98)	heat, cold	max, avg daily temperature	lag 0 to lag 12	1°C increase/decrease in temperature above/below 18°C	age	non-accidental causes, heat: 2.8%; cold: 0.49%

Rocklöv et al. 2014	1990-2002	Stockholm County, Sweden	total	heat	Tmax	lag 0-1, lag 0-6	OR associated with degree increase of temperature	sex, age, pre-existing disease, country of origin, municipality level wealth	Gradual increases in summer temperatures associated with mortality in those >80 yrs, and with mortality in those with previous myocardial infarction and with COPD in those <65 yrs; During winter, mortality associated with decreased temperature particularly in men and with duration of cold spells for those >80 yrs. History of hospitalization for myocardial infarction increased OR associated with cold among those >65 yrs.
Rocklöv et al. 2011	1990-2002	Stockholm County, Sweden	total (except 800-999, E); CVD (390-459, I); respiratory (460-519, J)	heat, cold	max and min T_{app}	lag 0-1, 0-6, 0-13	1°C increase (or decrease) in min T_{app}	age, summer/winter	Extreme heat associated with higher death rates in adults and for CVD death, compared with increased temperature. Warmer temperatures increase daily total mortality, while decreasing colder temperatures increase risk of CVD deaths.
Rocklöv et al. 2009	1990-2002	Stockholm, Sweden	all-cause excluding violent deaths, influenza (487, J10-11); respiratory (460-519, J); CVD (390-459, I); CVD plus respiratory (390-519, I-J)	heat, cold	daily mean temperature	lag 0-1	per 1°C increase above/below the threshold	previous winter mortality	Cumulative effect 0.95% below and 0.89% above threshold (21.3°C) after winter with low CVD and respiratory mortality, and -0.23% below and 0.21% above threshold after winter with high CVD and respiratory mortality.
Rocklöv and Forsberg 2010	1998-2005	Stockholm, Göteborg and Skåne, Sweden	natural causes excluding external causes	heat	daily mean temperature	lag 0–1, 2–6 7–13	1°C above 90th percentile of summer temperature	age, RH	Effect of temperature on mortality was found distributed over lag 0 or lag 1, with cumulative combined RR of about 5.1% (95% CI 0.3-10.1)
Rocklöv and Forsberg 2008	1998-2003	Stockholm, Sweden	non-external; CVD respiratory	heat, cold	daily mean temperature	lag 0 to lag 10, lag 0-10	1°C increase/decrease in temperature above/below optimal temperature (11–12°C)	age	heat: cumulative general RR 1.4% increase (95% CI 0.8–2.0%); cold: 0.7% (0.5–0.9%) decrease
Schaeffer et al. 2016	2000-2006 excluding heat wave of 2003 (Aug. 1-31)	Paris, France	non-accidental (A00-R99)	heat, cold	min, max and mean daily temperature	lag 0, lag 1-7	heat: 99th percentile relative to 90th percentile; cold: 1st vs. 10th percentile	study zone, age	heat: for those >75 yrs (RR 1.10, 95 % CI 1.07-1.14); cold: for those <75 yrs (1.04, 1.01-1.06)

Stafoggia et al. 2009	1987-2005	Rome, Italy	natural (1–799); CVD (390–459); respiratory (460–519)	heat	T_{app}	lag 0-1	30°C relative to 20°C	previous winter mortality	Effect stronger in years characterized by low mortality in previous winter (RR for days at 30°C vs. 20°C: 1.73 (95% CI 1.50 –2.01), contrasted with years with medium (1.32, 1.25–1.41) or high previous winter mortality (1.34, 1.17–1.55).
Stafoggia et al. 2008	1997-2004	4 Italian cities	non-injury (1–799)	heat	T_{app}	lag 0-1	30°C relative to 20°C	age, sex, socioeconomic characteristics, hospital ward, and type of hospital	OR for total 1.32 (95% CI 1.25-1.39); age, marital status and hospital ward were important risk indicators.
Stafoggia et al. 2006	1997–2003	4 Italian cities (Bologna, Milan, Rome, and Turin)	non-injury (1–799)	heat	mean T_{app}	lag 0-1	pooled ORs at 30°C relative to 20°C	age, sex, marital status, income, hospital admission in the 2 previous yrs, place of death	OR 1.34 (95% CI 1.27–1.42) at 30°C relative to 20°C; OR increased with age; OR higher among women (1.45, 1.37–1.52) and among widows and widowers (1.50, 1.33–1.69). Low area-based income modestly increased effect.
Tobias et al. 2014	June–Sep., 1990-2004	50 Spanish cities	all-cause (1–799)	heat	daily mean, min and max temperatures	lag 0-2	º1C increments at 99th percentile compared to 90th percentiles of whole-year temperature distributions	geographic (altitude, latitude, longitude and surface), socio-demographic (total population, proportion of population >65 yrs and per capita income), climatic characteristics (yearly and summer temperatures, RH)	Risk increased 3.3% per 1°C between 90th and 99th percentiles. Although risk increments varied by city, the range of temperature (from 90th to 99th percentiles) was the only characteristic independently significantly associated with risks. Heat increment did not depend on other city climatic, socio-demographic and geographic determinants.
Urban et al. 2014	1994-2009	Czech Republic	CVD (I00–I99)	heat, cold	avg daily temperature; days with avg temperature above/below the 90%/10% quantile of empirical distribution in summer (June-Aug.) and winter (Dec.-Feb.) defined as warm/cold days.	up to 14 days	risk on warm days (10% warmest days in summer)/cold days (10 % coldest days in winter)	sex, urban/rural region	Generally higher relative excess CVD mortality on warm days than cold days in both regions
Vigotti et al. 2006	1980-1989	Milan, Italy	all natural (1–799)	heat	daily mean temperature	lag 0-1	1°C increase above estimated thresholds	birthplaces	mortality risks differ by birthplace, regardless of place of residence.

Wichmann et al. 2011	1999–2006	Copenhagen, Denmark	respiratory; CVD	heat	max T_{app}	lag 0 to 5, 0-1, 0-5	IQR (7°C) increase	age, sex, socio-economic status and place of death	Inverse association with CVD (−7%, 95% CI −13%, −1%) and no association with respiratory and cerebrovascular. In cold period all associations were inverse, although insignificant.
Zeka et al. 2014	1984-2007	Republic of Ireland (ROI) and Northern Ireland (NI)	non-accidental (001-799, A00-R99); CVD (390–429; I01-I52); respiratory (460–519, J0-J99)	cold	daily max temperature	lag 0, means of lag 1-2, 3–5, 6–9, 10–14, 15–21, 22–28 and 29–35	1°C decrease	sex, age	In ROI, cumulative mortality increase for all-cause 6.4% (95% CI 4.8-7.9%); similar increases for CVD, and twice as much for respiratory; In NI, associations less pronounced for CVD causes.
South America									
Gouveia et al. 2003	1991-1994	Sao Paulo, Brazil	all-cause excluding violent deaths (chapter 17); CVD (390–459); respiratory (460–519)	heat, cold	daily mean, max and min temperature	lag 0-1, lag 0-20	per degree increase/decrease in temperature above/below 20°C	age group, and socioeconomic position (SEP)	Among elderly 2.6% increase in all-cause mortality per degree increase in temperature >20°C; 5.5% increase per degree decrease in temperature <20°C
O'Neill et al. 2005a	1996–1998 (Mexico City), 1996–1999 (Monterrey)	Mexico City and Monterrey, Mexico	non-external (excluding >E800, V01-Y89)	heat, cold	T_{app}	heat: lag 0-2; cold: lag 0-6	% change in daily mortality at 35–36°C T_{app} relative to 25–26°C /at 10–11°C T_{app} vs. 25–26°C /at 10–11°C T_{app} vs. 15–16°C	age	Mexico City: cold effect 12.4% (95% CI 10.5-14.5%); Monterrey: cold effect 11.7% (3.7-20.3%), heat effect 18.7% (11.7-26.1%)
Son et al. 2016a	May 1996-Dec. 2010	São Paulo, Brazil	all-cause (A00-R99); CVD (I00-I99); respiratory (J00-J99)	heat, cold	daily mean temperature (24h avg)	heat: lag 0-1; cold: lag 0-20	heat: 90th percentile relative to 50th percentile (24.1°C vs. 20.4°C) and 99th vs. 90th percentile (26.2°C to 24.1°C); cold: 10th vs. 50th percentile (16.5°C vs. 20.5°C) and 1st vs. 10th percentile (14.3°C vs. 16.5°C)	sex, age, education, marital status, and place of death; heat wave characteristics	heat: all-cause 6.1% (95% CI 4.7-7.6%) higher at 99th vs. 90th percentile; cold: 8.6% (6.2-11.1%) higher at 1st vs. 10th percentile
**Oceania**									

Cheng et al. 2017	2000-2009	5 cities (Sydney, Melbourne, Brisbane, Perth and Adelaide), Australia	all causes	heat	mean temperature; TV as SD of hourly temperature within 2 days	lag 2-3 for Perth, lag 0-1 for other cities	each 1 °C and IQR increases in TV	city-specific climate, geographic, demographic, health-related (chronic disease prevalence status), behavioral pattern, socioeconomic status for regions	Significant associations between TV and mortality in all cities; Deaths associated with each 1 °C rise in TV elevated by 0.28% (95% CI: 0.05, 0.52%) in Melbourne to 1.00% (0.52, 1.48%) in Brisbane, with a pooled estimate of 0.51% (0.33, 0.69%) for Australia; Subtropical and temperate regions showed no apparent difference in TV impacts; mortality risk could be influenced by city-specific factors: latitude, mean temperature, population density and the prevalence of several chronic diseases.
Hales et al. 1999	June 1988 - Dec. 1993	Christchurch, New Zealand	all cause; CVD (402-429); respiratory (460-519)	heat	Tmax	lag 1	increase of 1°C above the 3rd quartile (20.5°C) of Tmax	age	1% (95% CI 0.4-2.1%) for all-cause; 3% (0.1-6.0%) for respiratory
Qiao et al. 2015	Jan. 1996 - Nov. 2004	Brisbane, Australia	non-accidental (1–799, A00–R99); CVD (390–459, I00–99); respiratory (460–519, J00–99)	heat	mean temperature	lag 0-1	1°C increase above 28°C in summers that followed a winter with low mortality, compared with following winter with high mortality	previous winter mortality	heat effect generally stronger with low preceding winter mortality; 22% (95% CI 14-30%) increase in non-accidental mortality followed winter with low mortality, compared with 12% (7-17%) following winter with high mortality
Vaneckova et al. 2010	Oct.-March, 1993-2004	Sydney, Australia	non-external (001-799, A00-R99)	heat	daily avg temperature	running mean of 30 previous days	10°C increase in avg temperature during study period	socioeconomic status, proportion of vegetation or developed land, region specific, air pollutants	Spatial variation in mortality on unusually hot days observed among those $\geq$65 yrs; Elderly living within 5–20 km south-west and west of Sydney Central Business District more vulnerable.
Vaneckova et al. 2008	Oct.-March, 1993-2004	Sydney, Australia	all-cause (001–799, A00–R99); circulatory (390–459, I00–I99); respiratory (460–519, J00–J99)	heat	max daily temperature	not specified	10°C increase	age	With adjustment for air pollution, change in mortality was between 4.5% and 12.1% depending on mortality data set. Without air pollution adjustment, effect on mortality percentages changed by -1.1% to 0.9%. Tmax significantly associated with mortality in Sydney, with confounding by PM_{10} and O_3.

Williams et al. 2012	July 1993 – March 2009	Adelaide, South Australia	all	heat	daily max and min temperatures	not specified	10°C increase in max temperature above threshold and extreme temperatures (≥40 °C Tmax; ≥26 °C Tmin)	age	Association between temperature over thresholds and daily mortality not significant when adjusted for O_3 and PM_{10}. At extreme temperatures mortality increased significantly with increasing heat duration.
Yu et al. 2011a	1996-2004	Brisbane, Australia	non-external (001–799, A00–R99); CVD (390–459, I00-I79); respiratory (460–519, J00-J99)	heat, cold	mean, min, and max temperature	heat: 3 days; cold: 20 days	1º increase/decreases above/below threshold	age, different temperature indicators	AIC minimized when mean temperature used for non-external deaths and deaths for those 75-84 yrs; when Tmin used for those 0-64, 65–74, ≥85 yrs, and from respiratory diseases; and when Tmax was used for CVD. Effect estimates using certain temperature indicators were similar as mean temperature both for current day and lag effects.
Yu et al. 2011b	1996-2004	Brisbane, Australia	CVD (390-499, I00-I99)	heat, cold	daily mean temperature	lag 0-1, 0-7, 0-15, 0-21, 0-30	1°C increase (or decrease) above (or below) 24°C	age	heat: 3.7% (95% CI 0.4% to 7.1%) for people ≥65 yrs and 3.5% (0.4-6.7%) for all ages; cold: 3.1% (0.7-5.7%) for people >65 yrs and 2.8% (0.5-5.1%) for all ages
Yu et al. 2011c	1996-2004	Brisbane, Australia	all-cause (001-799, A00-R99); CVD (390-459, I00-I79); respiratory (460-519, J00-J99)	heat, cold	daily mean temperature	heat effect: lag 0, lag 0-2; cold effect: lag 0, lag 0-20	1°C increase above 24°C, 1°C decrease below 24°C	age	heat: highest % increase in mortality on lag 0 among people >85 yrs (7.2% (95% CI 4.3-10.2%)); cold: % increases in mortality at lag 0-20 3.9% (1.9-6.0%) and 3.4% (0.9-6.0%) for those >85 yrs and with CVD diseases, respectively.
Yu et al. 2010	Jan. 1, 1996 – Dec. 17, 2004	Brisbane, Australia	all-cause (001–799, A00–R99)	heat	daily mean temperature	not specified	1 degree increase in avg temperature (≥24°C)	age, sex, socioeconomic status	Clear increasing trend of harmful effect of high temperature on mortality with age. Effect estimate among women >20 times that of men. Did not find effect modification by SES.
**Africa**									

Azongo et al. 2012	1995-2010	Northern Ghana	all-cause	heat	mean daily temperature	lag 0-1, 2-6, 7-13	1°C increase in mean daily temp below/above 25th/75th percentile	sex, age	For all populations, statistically significant association of mean daily temperature lag0-1 below and above 25th (27.48°C) and 75th (30.68°C) percentiles (0.19%, 95% CI 0.05-0.21%) and (1.14%, 0.12-1.54%), respectively.
Diboulo et al. 2012	1999-2009	Nouna, Burkina Faso	all-cause	heat	mean temperature	lag 0-1, 2-6, 7-13	1°C increase	sex, age	Associations between higher temperature and daily mortality in Nouna HDSS, Burkina Faso; short-term direct heat effect particularly strong for <5 yrs mortality rate
Wichmann 2017	2006-2010	Cape Town, Durban and Johannesburg, South Africa	non-accidental (A00-R99)	heat	T_{app}	lag 0-1	IQR increase in T_{app}	sex, age groups (<15, 15-64, ≥65 yrs)	Elderly were more at risk in Cape Town and Johannesburg; No difference in risk observed between males and females in the 3 cities; an overall significant increase of 0.9% in mortality observed for all age groups combined in the 3 cities; For those ≥65 yrs a significant increase of 2.1% in mortality was observed.
**Multi-country studies**									
Analitis et al. 2008	Oct.–March, 1990-2000	15 European cities	natural (1–799); CVD (390–459); respiratory (460–519)	heat	min T_{app}	lags 0-15	1°C increase	age	1.35% (95% CI 1.16-1.53), 1.72% (95% CI 1.44-2.01), 3.30% (2.61-3.99), and 1.25% (0.77-1.73) increase in total, CVD, respiratory, and cerebrovascular deaths, respectively.
Baccini et al. 2011	April-Sep., 1990-2001	15 European cities	natural (1-799)	heat	max T_{app}	lag 0-3	1°C increase in max T_{app} above the threshold	age	Mean AF of deaths 2%. Highest impact in 3 Mediterranean cities (Barcelona, Rome and Valencia) and 2 continental cities (Paris and Budapest).
Baccini et al. 2008	April-Sep., 1990-2000	15 European cities	all-cause (1–799); CVD (390–459); respiratory (460–519)	heat	max T_{app}	lag 0 to lag 40	1°C increase in max T_{app} above city-specific threshold	region, age	all-cause: 3.12% (95% credibility interval 0.60-5.72%) in Mediterranean region, 1.84% (0.06-3.64%) in north-continental region

Bell et al. 2008	1998-2002	Mexico City, Mexico; São Paulo, Brazil; Santiago, Chile	non-accidental (excluding ICD-10 codes S and above); respiratory (J 100–118, 120–189, 209–499 and 690–700); CVD (I<800)	heat	mean T_{app}	lag 0 to lag 3; lag 0-1, 0-2, 0-3, 0-4, 0-5, 0-6	95th percentile relative to 75th percentile	confounding by air pollution, cause of death and susceptibilities by educational attainment, age and sex	For those ≥65 yrs: 2.69% (95% CI 2.06-7.88%) for Santiago, 6.51% (3.57–9.52%) for São Paulo, and 3.22% (0.93–5.57%) for Mexico City
Chung et al. 2017	1972-2009	15 cities in Northeast Asia	all-cause; CVD (390-458, 390-459, I00-I99); respiratory (460-519, 460-519, J00-J99)	heat, cold	mean temperature	heat: lag 0-2; cold: lag 0-7	cold: RR at the 1st and 10th percentiles of temperature; heat: RR at the 99th vs 90th percentiles	age, location	Cold-related mortality remained roughly constant over decades and slightly increased in the late 2000s, with a larger increase for cardiorespiratory deaths than for deaths from other causes. Heat-related mortality rates have decreased continuously over time, with more substantial decrease in earlier decades, for older populations and cardiorespiratory deaths.
De' Donato et al. 2015	April-Sep., 1996-2010	9 European cities	all-cause (1–799); CVD (390–459); respiratory (460–519)	heat	mean temperature	up to 40 days	increases in mean temperature from the 75th to 99th percentile of the summer distribution; two 7-year periods were compared: 1996–2002, 2004–2010	age	In the recent period (2004-2010), reduction in mortality risk associated with heat only in Athens, Rome and Paris, especially among the elderly. In Helsinki and Stockholm, suggestion of increased heat effect. An effect of heat was still present in the recent years in all cities, ranging from +11% to +35%.

Guo et al. 2014	Australia (3 cities 1988-08), Brazil (18 cities 1997–11), Thailand (62 provinces 1999-08), China (6 cities 2002-11), Taiwan (3 cities 1994–07), South Korea (7 cities 1992–10), Japan (7 cities 1972–09), Italy (10 cities 1987-10), Spain (51 cities 1990-10), UK (10 regions 1993-06), US (108 cities 1987-00), Canada (21 cities 1986-09)	306 communities from 12 countries/regions	total	heat, cold	mean temperature	max lag: up to lag 21	cold: RRs (1st percentile vs. MMT); heat: RRs (99th percentile vs. MMT)	avg temperature, avg latitude	Temperatures associated with lowest mortality around 75th percentile of temperature in all countries/regions, ranging from 66th (Taiwan) to 80th (UK) percentiles. Estimated effects of cold and heat varied by community and country. Meta-analysis found that cold and hot temperatures increased risk in all the countries/regions.

Guo et al. 2011	summer: Brisbane (1996–2004), Los Angeles (1987–2000)	Brisbane, Australia; Los Angeles, US	non-external (001-799, A00-R99); CVD (390–459, I00–I79); respiratory (460–519, J00–J99)	heat	temperature change as lag 0 mean temperature minus lag 1 mean	lag 0	1°C increase in temperature change	sex, age	In Brisbane, a decrease of >3°C in temperature between days associated with RRs of 1.157 (95% CI 1.024-1.307) for non-external mortality (NEM), 1.186 (1.002-1.405) for NEM in females, and 1.442 (1.099-1.892) for those 65–74 yrs. An increase >3°C associated with RRs of 1.353 (1.033-1.772) for CVD mortality and 1.667 (1.146-2.425) for <65 yrs. In Los Angeles, only a decrease of >3°C was significantly associated with RRs of 1.133 (1.053-1.219) for total NEM, 1.252 (1.131-1.386) for CVD mortality, and 1.254 (1.135-1.385) for ≥75 yrs.
Hajat et al. 2005	January 1991 - Dec. 1994	Delhi, India; São Paulo, Brazil; London, England	all-cause excluding violent deaths; CVD (390–459); respiratory (460–519)	heat	daily avg temperature as mean of daily max and min	lag 0, 0 to 1 week, 0 to 4 weeks	1ºC increase in temperature >20°C	age	2.4% (95% CI 0.1-4.7%) per degree higher than heat threshold in Delhi, 0.8% (0.4-2.1%) in São Paulo, and 1.6% (3.4-0.3%) in London
Ishigami et al. 2008	Budapest 1993–2001; London 1993–2003; Milan 1999–2004	3 European cities	all-cause; CVD (390.0–459.9, I); respiratory (460.0–519.9, J)	heat	avg temperature	lag 0-1	1°C increase in temperature above threshold	socioeconomic status, census data	RRs: Budapest (≥24°C): (i) Male 1.10 (95% CI 1.07–1.12) and female 1.07 (1.05–1.10) for 75–84 yrs, (ii) Male 1.10 (1.06–1.14) and female 1.08 (1.06–1.11) for ≥85 yrs; London (≥20°C): (i) Male 1.03 (1.01–1.04) and female 1.07 (1.05–1.09) for 75-84 yrs, (ii) male 1.05 (1.03–1.07) and female 1.08 (1.07–1.10) for ≥85 yrs; and Milan (≥26°C): (i) male 1.08 (1.03–1.14) and female 1.20 (1.15–1.26) for 75-84 yrs, (ii) male 1.18 (1.11–1.26) and female 1.19 (1.15–1.24) for ≥85 yrs.

Kim et al. 2016	1979-2010	30 cities in China, Japan, Korea, and Taiwan	all-cause (A00-R99); circulatory (I00-I99); respiratory (J00-J99)	heat	DTR	lag 0, 1-4, 0-4	per 1°C increase in DTR	age	Adverse effects of DTR more pronounced for those ≥65yrs and varied by geographic, longitudinal (0.07%; 95% CI: 0.05, 0.10), and climatic characteristics and scale of DTR (0.33%; 95% CI: 0.12, 0.55) for overall all-cause mortality.
Lim et al. 2015	Taiwan: 3 cities (1994-07); Korea: 7 cities (1992-10, with 1997–10 for Ulsan); Japan: 6 cities (1979-09)	3 cities in Taiwan; 7 cities in Korea; 6 cities in Japan	non-accidental (A00-S99)	heat	mean temperature	lag 0-1	% changes in mortality risk per 1°C increase in mean temperature during extremely-high-temperature (≥95% of daily mean temperature) days	city-level GDP per capita as a proxy of city income level	In cities with a low GDP per capita (<20,000 $USD), effects of temperature detrimental when long-term avg summer temperature was high. In cities with high GDP per capita, temperature-related mortality risk not significantly related to avg summer temperature.
Scovronick and Armstrong 2012	1996–2015	South Africa	all-cause	heat, cold	mean temperature	cold: lag 3–13; heat: lag 0–2	% increase per degree above/below threshold	housing type	Different housing types protected differently against effects of high and low temperatures.
The Eurowinter Group 1997	1988-1992	north Finland, south Finland, Baden-Württemburg, the Netherlands, London, and north Italy	all-cause (0–999); CVD (430.0–438.9); respiratory (460.0–519.9)	cold	mean temperature	lagged by 5 days for CVD; 12 days for respiratory; and 3 days for all-cause	per 1°C decrease below 18°C	sex, age, population characteristics	% increases in all-cause mortality higher in warmer regions than in colder regions (e.g., Athens 2.15% [95% CI 1.20–3.10] vs south Finland 0.27% [0.15–0.40])

Note: ICD codes includes ICD 9 and/or ICD 10; Lag means days unless otherwise specified; We reported main findings regardless of statistical significance, presented by study authors, as originally reported

Table ~~S2~~S3. Description of studies of heat waves and cold spells and mortality included in the review

Studies	Period	Location	Mortality (ICD codes)	Exposure Type	Exposure Metric	Lag	Exposure Increment	Effect Modification Studied	Main Findings
**Asia**									
Ahmadnezhad et al. 2013	May-Sep., 2001-2011	Tehran, Iran	non-external (A00-R99); CVD; respiratory	heat waves	heat waves (≥3 consecutive days with Tmax >90th percentile (37.8 °C); or ≥3 consecutive days with Tmin >90th percentile (25.7 °C) and Tmax > monthly mean temperature)	lag 0, 0-1, 0-2 days	1°C increase during heat waves	age, sex	All non-external causes during heat waves compared to non-heat wave days: RR 1.03 (95% CI 1.01-1.05) and 1.09 (1.07-1.09) after adjusting for ozone and PM_{10}, respectively.
Chen et al. 2015	2007-2013	Nanjing, China	total (A00–R99); CVD (I00–I99); respiratory (J00–J99)	heat waves	heat waves (≥4 consecutive days with daily average (avg) temperature >98th percentile); This definition had best model fit.	lag 2	heat wave days compared with non-heat wave days	sex, age, education level, location of death	24.6% (95% CI 15.6-34.3%), 46.9% (33.0-62.3%), and 32.0% (8.5-60.5%) for total, CVD, and respiratory, respectively
Han et al. 2017	Nov.–March and May–Aug., 2011-2014	Jinan, China	non-accidental (A00-R99); CVD (I00–I99); respiratory (J00–J99)	heat waves, cold spells	as ≥3 consecutive days with mean temperature ≤5th percentile or ≥95th percentile	not specified	risk during heat wave days compared to non-heat wave days	gender, age	Both cold spells and heat waves increase risk of death in Jinan, China; The elderly were more vulnerable during heat waves; vulnerability to cold spell did not vary by age or gender.
Huang et al. 2010	2003 heat waves	Shanghai, China	non-accidental (A00-R99); CVD (I00-I99); respiratory (J00-J98)	heat waves	heat waves (>3 consecutive days with Tmax >35 °C)	not specified	heat wave days vs. non-heat wave days	sex, age	total mortality RR 1.13 (95% CI 1.06–1.20); CVD (1.19, 1.08–1.32); respiratory (1.23, 1.02–1.48)
Lan et al. 2012	2010 heat waves	Harbin, China	total (A00-R99)	heat waves	heat waves (≥3 consecutive days with daily max temperature > 98th percentile)	not specified	heat waves period (June 7–11, 2010) vs. reference period (June 8–12, 2009)	sex, age groups, place of death	RR of total mortality 1.41 (95% CI 1.22–1.63)

Lin et al. 2011	1994-2007	4 major cities, Taiwan	all-cause; circulatory (390-459); respiratory (460-519)	heat extremes, cold extremes	heat extremes: city-specific avg temperature ≥95th, 97th percentiles for 3-5, 6-8 days and >8 days, 99th percentiles for 2-3 days and >3 days; cold extremes: city-specific avg temperature ≥10th, 5th percentiles for 3-5, 6-8 days and >8 days, 1st percentiles for 2-3 days and >3 days	lag 0 to lag 30	risks during heat or cold extremes compared with the non-extreme event days	different definitions for heat, cold extremes	mortality risk slightly increased with strengthened and prolonged heat extremes (≥99th and >3 days; ≥97th and >8 days; and ≥95th and >8 days) with RRs from 1.04–1.05, 1.01–1.05, and 1.05–1.13 for all-causes, circulatory, and respiratory mortality, respectively
Ma et al. 2015	May-Sep., 2006-2011	66 Chinese communities	all-cause (A00-R99); CVD (I00-I99); respiratory (J00-J99)	heat waves	heat waves (≥2 consecutive days with mean temperature ≥95th percentile of the year-round community-specific distribution)	lag 0-1	% increase in daily mortality during heat wave days vs. non-heat wave days for IQR increase in community level variables	age, sex, cause of death, education level or place of death, community characteristics (marital status, % unemployed, per capita GDP, latitude, population size, ownership of AC per 100 households and urbanization)	5.0% (95% CI 2.9–7.2%) excess deaths, with highest excess deaths in north China at 6.0% (1–11.3%), followed by east China at 5.2% (0.4–10.2%) and south China at 4.5% (1.4–7.6%).
Ma et al. 2013	2001-2009	Shanghai, China	non-accidental (<800, A00-R99); CVD (390–459, I00-I99); respiratory (460–519, J00-J98)	cold spells	cold spells (≥7 consecutive days with daily temperature <3rd percentile)	not specified	RRs during cold spells and compared with winter reference period (Jan. 6–9, and Feb. 28 to Mar. 2)	sex, age	13% (95% CI 7–19%), 1.21 (1.12–1.31), 1.14 (0.98–1.32) for non-accidental, CVD, and respiratory, respectively
Son et al. 2012	May-Sep., 2000-2007	7 major cities, South Korea	all-cause (A00–R99); CVD (I00–I99); respiratory (J00–J99)	heat waves	heat waves (≥ 2 consecutive days with daily mean temperature ≥98th percentile for warm season in each city)	lag 0	heat wave days compared to non-heat wave days	individual characteristics (sex, age, education level, place of death); heat wave characteristics (intensity, duration, and timing in season)	all-cause mortality: 4.1% (95% CI –6.1, 15.4%); for Seoul 8.4% (0.1-17.3)

Sun et al. 2014	Jan. 1, 2008 to June 15, 2013	Pudong New Area, China	all-cause; CVD (I00–I99); respiratory (J00–J98)	heat waves	heat waves (≥3 consecutive days with Tmax >35 °C)	lag 0 to lag 6; avg of lag 1 and lag 2; avg of lags 1, 2 and 3; avg of lag 1–6	during 2013 heat waves	sex, age	Females (male: 10.43%, female: 11.79%) and people ≥ 80 yrs (excess deaths were 129 (95% CI: 47-203) and excess mortality was 16.64%) were strongly affected by heat waves. Excess CVD and respiratory mortalities were 22.34% and 20.68% respectively, higher than that of all-cause deaths.
Wang et al. 2016	2006-2011	66 communities, China	non-accidental (A00-R99); respiratory (J00-J99); CVD (I00-I99); cerebrovascular (I60–I69)	cold spells	cold spells: mean daily temperature <5th percentile of study period (cold season 2006–2011) in a specific community for ≥ 2 consecutive days	lag 0-27	excess risk during cold spell days compared with non-cold spell days	cold spell characteristics (intensity, duration, and timing), age, gender, education	Significant increase in mortality when cold spell duration and intensity increased or occurred earlier in the season. Cold spell effects and effect modification by cold spell characteristics were more pronounced in south China. The elderly, people with low education level and those with respiratory diseases were generally more vulnerable to cold spells.
Xie et al. 2013	2006-2009	Guangdong Province, China	non-accidental (A00-R99); CVD (I00-I99); respiratory (J00-J99)	cold spells	cold spells (min daily temperature <5th percentile of temperatures recorded at that location for ≥5 consecutive days)	lag 0, 0-6, 0–13, 0–20, 0–27	during the 2008 cold spells	age, sex	cumulative risk of non-accidental mortality increased significantly in Guangzhou (RR 1.60; 95% CI 1.19-2.14) and Taishan (1.60, 1.06-2.40) when lagged up to 4 weeks after cold spell ended.
Yang et al. 2013	2003-2006	Guangzhou, China	non-accidental (A00-R99); CVD (I00-I99); respiratory (J00-J98)	heat waves	heat waves as ≥7 consecutive days with daily max temperature >35.0°C and daily mean temperature >97th percentile	3 lag days	risk in the case period vs. reference period in same summer	sex, age, educational level, occupation class	non-accidental mortality: RR 1.23 (95% CI 1.11-1.37); CVD: 1.34 (1.13-1.59); respiratory: 1.31 (1.02-1.69)

Zeng et al. 2014	May-Sep., 2006-2010	4 communities in Guangdong Province, China	all-cause (A00-R99)	heat waves	heat waves as ≥2 consecutive days with daily mean temperature ≥95th percentile of year-round temperature	lag 0, 0-2, 0-4, 0-8, 0-10	main effect: % increase in mortality predicted at median temperatures among heat wave days vs. 65th percentile of annual temperature distribution for each community; added effect: % increase in mortality during heat wave days vs. non-heat wave days	individual level (cause of death, age and gender) and heat wave characteristics (intensity, duration and timing), community characteristics (educational levels, % elderly, Gross Regional Domestic Product (GDP))	Main effects of excess risk 8.2% (95% CI 3.4-13.2%) higher than added effects of excess risk 0.0% (−3.8, 4.0%) at lag 0. Modifying effects of heat wave characteristics and community characteristics on mortality not statistically significant.
Zhang et al. 2016	June to Aug., 2012-2013	Jinan, China	non-accidental (A00-R99); circulatory (I00-I99); respiratory (J00-J99)	heat waves	heat waves as ≥3 consecutive days with daily mean daily Tmax > 35 °C.	up to lag 5	risks during heat wave compared to control period	gender, age (<65, 65-74, 75+ yrs), marital status, education attainment, place of death	Older people more likely to die during heat waves (OR1.233, 95 % CI 1.076–1.413) and more deaths occurred outside a hospital during heat waves (1.142, 1.006– 1.296).
Zhou et al. 2014	2006-2010	15 provinces, subtropical China	non-accidental (A00-R99); respiratory (J00–J99); CVD (I00-I99); cerebrovascular (I60-I69)	cold spells	cold spell as min daily temperature < 5th percentile of temperature for ≥5 consecutive days	up to 27 days	risk of 2008 cold spells compared to non-cold spell days	sex, age, urban/rural	Effects more pronounced for respiratory mortality than CVD or cerebrovascular mortality, for females than males, and those ≥75 yrs than for younger people.
**North America**									

Anderson and Bell 2011	May-Sep., 1987–2005	108 U.S. urban communities	non-accidental	heat waves	heat waves (≥ 2 consecutive days with daily mean temperature > community's 95th percentile warm season mean temperature)	lag 0	heat wave days vs. non-heat wave days; 1°F increase in avg mean temperature during heat waves (intensity); 1-day increase in heat waves duration; first vs. later heat waves	heat wave characteristics (intensity, duration, timing in season)	Mortality increased 3.74% (95% PI 2.29–5.22%) comparing heat wave and non-heat wave days; Heat wave mortality risk increased 2.49% for every 1°F increase in heat wave intensity; 0.38% for every 1-day increase in heat wave duration; Heat wave risk 5.04% (3.06–7.06%) for the 1st heat wave of summer vs. 2.65% (1.14–4.18%) for later heat waves.
Barnett et al. 2012	1987–2000	99 US cities	all-cause; CVD respiratory	heat waves, cold waves	cold waves (temperature < cold threshold for ≥2 consecutive days (1st to 5th percentiles); heat waves (temperature > heat threshold for ≥2 consecutive days (95–99percentiles))	max lag: lag 21	risk during heat/cold waves	age, cold/heat waves characteristics	Cold waves associated with generally small and not statistically significant increases in mortality. Heat waves generally associated with increased mortality risk, particularly for hottest heat threshold. Cold waves of colder intensity or longer duration did not have higher effect estimates; cold waves earlier in the cool season had higher risk estimates, as did heat waves earlier in the warm season.
Bobb et al. 2011	1987–2005	105 U.S. cities	all-cause excluding known accidental causes	heat waves	heat waves (2 temperature thresholds: 97.5th and 81st percentile of daily max temperature)	lag 0, 1, 2	% increase in mortality on heat wave days compared to non-heat waves days	age	No single model best predicted risk across the majority of cities; for some cities heat wave risk estimation was sensitive to model choice. While model averaging led to posterior distributions with increased variance as compared to statistical inference conditional on a model obtained through model selection. Posterior mean of heat wave mortality risk is robust to accounting for model uncertainty over a broad class of models.

Chen et al. 2017	1990-2011	12 Texas Metropolitan Areas, US	all-cause; CVD (390-429, I01-I52); respiratory (460-519, J00-J99)	cold waves	cold waves as daily mean temperature < 1st, 5th, or 10th percentiles with periods of ≥2 consecutive days	lag 0-25	risk comparing cold wave days to non-cold wave days	age (0-64, 65-74, 75+ yrs)	Several metropolitan areas along the Texas Gulf Coast showed statistically significant cold wave-mortality associations.
Jian et al. 2017	May-Sep., 1997-2010	Alabama, US	non-accidental deaths (< 800, A–R) + heat-related death (E900, X30)	heat waves	3 heat wave indices: (1) used 1.645×SD + mean of daily mean temperature (95th percentile of a normal distribution); (2) used 1.282×SD + mean of daily mean temperature, as threshold (90th percentile of a normal distribution); (3) defined by 2 thresholds: 97.5th (T1) and 81st (T2) percentiles of daily Tmax. Heat wave period defined as (1) ≥ 3 days with a daily Tmax > T1, (2) daily Tmax > T2 for each day of the period, and (3) average of daily Tmax over the entire period > T1	not specified	percent differences in the odds between heat wave days and non-heat wave days	different cumulative environmental qualities (based on 5 domain indices (air, water, land, built, and sociodemographic))	Found significant associations between heat waves and non-accidental deaths and a significant effect modification of this relationship by environmental quality index (EQI). Higher ORs in counties with the worst cumulative environmental qualities compared to counties with the best cumulative environmental qualities. For example, the % change in OR (mean and 95% CI) between heat wave days and non-heat wave days was −10.3% (−26.6, 9.6) in counties with an overall EQI of 1 (best overall environment) and 13.2% (4.9, 22.2) in counties with an overall EQI of 3 (worst overall environment). Among the five domains, air quality had the strongest effect modification on the association.

Joe et al. 2016	June-Aug., 2006	California, US	internal (A00-R94); external (V01-Y89.9)	heat waves	HW: 18-day period (July 15-Aug. 1, 2006); reference period: from the same summer (June 1 to 30, July 6 to 14, and Aug. 8 to 31, 2006)	not specified	RRs during heat wave in 2006 comparing reference period	place of death, gender, race, age, climate zones	Total mortality risk higher among those 35–44 yrs than ≥65, and among Hispanics than whites; Deaths from external causes increased more sharply (RR 1.18, CI 1.10–1.27) than from internal causes (1.04, 1.02–1.07). Risk varied by building climate zone; highest risks of at-home death occurred in northernmost coastal zone (1.58, 1.01–2.48) and the southernmost zone of California's Central Valley (1.43, 1.21–1.68).
Kaiser et al. 2007	1993-1997	Cook County (containing Chicago), IL, US	non-accidental (excluding >800, except 900); CVD (390–429)	heat waves	the 1995 Chicago heat waves	lag 0 to lag 3	risk during Chicago heat waves	age, sex, race, education, sudden death	RR for all-cause mortality on the day with peak mortality was 1.74 (95% CI 1.67-1.81).
Kent et al. 2014	1990-2010	Alabama, US	non-accidental (< 800, A–R)	heat waves	different heat waves index definitions (15 versions) (Table 1)	lag 0 to lag 6	heat wave days relative to non–heat wave days	rurality	Associations varied by heat wave definition. Heat waves defined as ≥2 consecutive days with mean daily temperatures >90th percentile: 3.7% (95% CI 1.1-6.3%).
Madrigano et al. 2015a	2000-2011, warm season (May-Sep.)	New York, NY, US	non external (A00-R99, 001–799); CVD (I00–I99)	heat waves	heat waves days: days with max temperature or max heat index >95°F for ≥ 2 consecutive days	lag 1-2	heat wave days compared to other warm-season days	race/ethnicity, neighborhood characteristics	Heat wave deaths more likely in black non-Hispanic persons than others (OR 1.08; 95% CI 1.03-1.12), deaths at home than in institutions and hospital settings (1.11, 1.06-1.16), and among those living in census tracts that received more public assistance (1.05, 1.01-1.09). Heat wave deaths more likely among residents in areas with higher relative daytime summer surface temperature and less likely among residents living in areas with more green space.

Sheridan and Lin 2014	1991–2004	New York City, US	respiratory (J00-99); CVD (I00-99)	heat waves	SSC categories based on weather conditions from temperature, dew point, wind speed and direction, pressure, and cloud cover; hot days (categorized either as Moist Tropical Plus (MT+) or Dry Tropical (DT) weather type); length of heat waves: threshold of 3 consecutive days of MT+ or DT weather type	lag 0, cumulative 15-day lag	RR for heat wave days compared to non-heat wave days	heat wave characteristics	The impacts of heat are higher during longer heat events and during middle of summer, when increased mortality is statistically significant after accounting for mortality displacement. Early-season heat waves have increases in mortality that appear to be largely short-term displacement.
Zhang et al. 2015	2007-2011	Houston, Texas, US	all-cause (below S)	heat waves	2011 heat wave (Aug. 2–30, 2011); for > 2 consecutive days in 2011 Aug. with daily mean temperature > 95th, 97th, or 99th percentiles	max lag: lag 7	risk of heat waves compared to warm months (May to Sep.) from 2007 through 2011	age	2011 heat waves in Houston associated with 0.6% (95% CI −5.5%, 7.1%) mortality increase.
**Europe**									
Basagaña et al. 2011	May 15-Oct. 15, 1983–2006	Spain	total; CVD (I00-I99, 390-459); respiratory (J00-J99, 460-519)	heat waves	extremely hot days (max temperature >95th percentile, 3 consecutive hot days)	lag 0-2, 3-6, 0–6	during extremely hot days vs. non-extremely hot days	age, sex	3 consecutive hot days associated with 19% increase in total mortality. In infants, heat effect observed only for conditions originating in the perinatal period (RR 1.53, 95% CI 1.16–2.02).
Borrell et al. 2006	June-Aug., 1999-2003	Barcelona, Spain	all deaths	heat waves	daily max temperature	not specified	2003 compared to 1998–2002	age, sex, educational level	RR during 2003 summer compared to summers of 5 previous yrs higher for women than men and among older women (≥65 yrs).
Hutter et al. 2007	May–Sep., 1998-2004	Vienna, Austria	total	heat waves	heat wave (>3 consecutive days with daily max temperature ≥30 °C)	not specified	during heat wave days compared to non-heat wave days	sex, age	Heat wave days between 1998 and 2004 associated with increased RR 1.13 [95% CI 1.09–1.17].

Huynen et al. 2001	1979–1997	Netherlands	respiratory (AM 33–35); CVD (AM 25–32)	heat waves, cold spells	heat waves (≥5 days each with max temperature ≥25°C, including ≥3 days with max temperature ≥30°C); cold spells (≥9 days with min temperature ≤–5°C, of which ≥6 days have min temperature ≤-10°C)	lag 0, 1-2, 3-6, 7-14, 15-30; lag 0-30	risk during heat waves/cold spells days compared to non-heat waves/cold spells days	age	All heat waves studied were associated with increased mortality. The elderly were most effected by EH. Heat waves were associated with all causes of mortality studied, especially respiratory mortality. Avg total excess mortality during the heat waves studied was 12.1%, or 39.8 deaths/day. The avg excess mortality during cold spells was 12.8% or 46.6 deaths/day, which was mostly attributable to increased CVD mortality and mortality among the elderly.
Kysely et al. 2009	1986-2006	Czech Republic	CVD (390–459, I00–I99)	cold spells	cold spells (≥3 consecutive days with daily temperature max < -3.5°C)	max lag: lag 20	excess mortality during cold spells relative to baseline	age, sex	Cold spells associated with positive mean excess CVD mortality in all age groups (25–59, 60–69, 70–79 and ≥80 yrs and in men and women. Relative mortality effects most pronounced and most direct for men 25–59 yrs, which contrasts most studies on cold-related mortality in other regions.
Monteiro et al. 2013	2002-2007	Porto, Portugal	all-cause; respiratory	heat waves	heat waves (≥2 consecutive days with a (41<heat index<54))	max lag: lag 7	1°C increase in heat index during the heat waves week, previous week and following week	age, sex	all-causes: 2.7 % (95% CI 1.7–3.6 %)
Oudin et al. 2015	May 15-Sep. 15, 2000-2008	Rome, Italy and Stockholm, Sweden	all-cause	heat waves	heat waves (≥2 days exceeding the city-specific 95th percentile of max T_{app})	not clearly specified	% increase in daily mortality during heat wave days compared to non-heat wave days	age, sex	heat waves compared to non-heat wave days for those ≥50 yrs: 22% (95% CI 18-26%) in Rome and 8% (3-12%) in Stockholm

Rabczenko et al. 2016	May - Sep., 2008-2013	Warsaw, Poland	all-cause	heat waves, hot periods	heat waves defined as at least 3 consecutive days with max temperature higher than 30°C; Hot periods were defined as at least 3 consecutive days with average max temperature ≥30°C, among them hot days (Tmax ≥ 30°C) constitute at least half of the days and possible series of warm days (25°C≤Tmax < 30°C) among hot days cannot exceed 3 days.	lag 0-7	risk during heat waves compared to non-heat wave period	sex, age	Heat waves have additional (to temperature effect itself) effect on male mortality however, only in males aged 70 yrs; the effect was statistically significant.
Revich and Shaposhnikov 2010	1999-2007	Yakutsk, East Siberia	non-accidental (A00–R99); CVD (I60-I69)	heat waves, cold spells	heat wave (daily mean temperature >97th percentile of historic distribution of daily mean temperatures for ≥9 consecutive days, of which ≥3 days had avg daily temperatures >99th percentile); cold spells (≥9 consecutive days with daily mean temperatures <3rd percentile, of which ≥3 days had daily mean temperatures <1st percentile).	lag 0 to lag 20	during heat waves (cold spells) days compared to non-heat waves (cold spells) days	age	CVD and non-accidental mortalities increased in Yakutsk during heat waves and cold spells. Magnitude of established health effects approximately the same for heat and cold.

Revich and Shaposhnikov 2008a	Jan. 2000-Feb. 2006	Moscow, Russia	non-accidental; CVD; respiratory	heat waves, cold spells	heat waves (avg temperature >97th percentile during ≥5 consecutive days, of which ≥3 days have avg temperatures >99th percentile); cold spells (≥9 consecutive days with avg temperatures <3% percentile, of which ≥6 days have avg temperatures <1st percentile)	max lag: lag 28	during heat waves and cold spells compared to non-heat waves/cold spells days	age	Cumulative excess non-accidental mortality during 2001 heat waves was 33% (95% CI 20-46%). The cumulative effects of the 2 cold spells in 2006 on mortality were significant only for those ≥75 yrs, for which avg daily mortality from all non-accidental causes increased by 9.9% (8.0-12%) and 8.9% (6.7-11%).
Rocklöv et al. 2014	1990-2002	Stockholm County, Sweden	total	heat waves, cold waves	max temperature	lag 0-1, lag 0-6	OR associated with per additional day of heat or cold waves duration	sex, age, pre-existing disease, country of origin, municipality level wealth	Higher heat wave effect for: lower ages, areas with lower wealth, hospitalized patients <65 yrs. Odds elevated among females <65 yrs, those with previous hospital admission for mental disorders, and persons with previous CVD disease. Gradual increases in summer temperatures associated with mortality in those >80 yrs, those with previous myocardial infarction, and those with COPD <65 yrs. During winter, decrease in temperature associated with mortality particularly in men and with duration of cold spells for those >80 yrs. History of hospitalization for myocardial infarction increased odds associated with cold temperatures among those >65 yrs. Previous mental disease or substance abuse associated with higher odds of death among those <65 yrs.

Schifano et al. 2009	2005-2007	Rome, Italy	non-injury causes (1-799)	heat waves	max T_{app}	during heat episode or in following 3 days	heat wave days compared to non-heat wave days	socio-demographic characteristics and pre-existing medical conditions	For those 65-74 yrs, risk was higher among unmarried persons and those with previous hospitalization for chronic pulmonary disease or psychiatric disorders. Those ≥75 yrs, women, and unmarried subjects were more susceptible to heat.
Urban et al. 2017	1994-2015	Czech Republic	natural-cause (A00-R99)	heat waves	heat waves: at least 3 consecutive days with mean daily temperature higher than the 95th percentile of annual distribution	not specified	risk during the summer of 2015 compared with the summer of 1994	gender, age (0-64, 65+ yrs)	Excess mortality was comparable among the younger age group (0–64 yrs) and the elderly (65+ yrs) in the 1994 major heat wave while it was significantly larger among the elderly in 2015.
Xu et al. 2013	May 15-Oct. 15, 1999–2006	Barcelona, Spain	all-cause	heat waves	3 consecutive hot days (defined as those >95th percentile of max temperature)	lag 0, 1, 2	heat wave days compared with non-heat wave days	sociodemographic and urban landscape characteristics (% manual workers; % unemployed; % of those 16–29 yrs who are illiterate or did not complete primary school education (low education level), % ≥65 yrs; % of old buildings (built before 1920); % of houses without AC, % of residents perceiving little surrounding greenness, % single dwellings)	Effect of 3 consecutive hot days: 30% increase in all-cause mortality (RR 1.30, 95% CI 1.24-1.38).
**Oceania**									
Nitschke et al. 2007	1993–2004	metropolitan Adelaide, Australia	total; CVD (390–4599, I00–I99); respiratory (460–5199, J00–J99)	heat waves	heat waves (daily max temperature >35°C for ≥3 consecutive days)	not specified	daily mean incidence of mortality during heat waves	age	Total mortality, disease- and age-specific mortality did not increase with heat waves; Significant decreases were observed in CVD related mortality.

Nitschke et al. 2011	July 1993 - Dec. 2007	Adelaide, South Australia	total; CVD (390-4599, I00-99); respiratory (460-5199, J00-J99)	heat waves	daily max temp; heat waves (≥35°C for ≥3 days)	heat waves days	mortality rates during heat waves occurring 2008-2009 and 1993-2008 were compared with rates during all non-heat waves days (Oct. to Mar.).	age	2009 heat wave was associated with considerable increases in total mortality that particularly affected those 15-64 yrs (1.37, 95% CI 1.09-1.71), without associations in older age groups.
Tong et al. 2015	1988-2009	3 largest Australian cities	non-accidental	heat waves	heat waves (95th and 99th percentile of mean temperature for ≥2 consecutive days)	lag 0 to lag 3, lag 0-3	risk during heat waves compared to non-heat waves	sex, age	Consistent and significant increase in mortality during heat waves was observed in all cities. RR began to increase around 95th percentile of temperature, increased sharply at 97th percentile and rose at 99th percentile.
Tong et al. 2014a	Jan. 1996 – Nov. 2004	Brisbane, Australia	non-external (< 800, A00-R99); CVD (390–459, I00-I99); respiratory (460–519, J00-J99)	heat waves	heat waves (≥2 consecutive days with mean temperature > specified percentile) in warm season (Nov. to Mar.)	lag 0, 1, 2 or 3, lag 0–1, 0–2 or 0–3	RR during heat waves compared to non-heat wave days	warm, early warm, late warm season, age, intensity, duration	Higher risk for mortality in the 2nd half of warm season than that in the 1st half
Tong et al. 2014b	1988-2009	3 largest Australian cities: Brisbane, Melbourne, and Sydney	non-external causes	heat waves	heat waves (mean temperature above a heat threshold (90th, 95th and 99th percentiles of mean temperature) for ≥2 consecutive days in summer season)	lag 0 to lag 7, lag 0-2	mortality during heat waves compared to non-heat waves	sex, age	Using the heat waves definition as 95th percentile of mean temperature for ≥2 days in summer season, RR for total mortality at lag1 in Brisbane, Melbourne and Sydney was 1.13 (95% CI 1.08-1.19), 1.10 (1.06-1.14), and 1.06 (1.01-1.10), respectively.

Wang et al. 2015	1988-2011	3 largest Australian cities (Brisbane, Melbourne and Sydney)	non-accidental; circulatory	heat waves	heat waves (mean temperature > specified percentile (e.g., 90th, 95th, 98th and 99th percentiles of mean temperature) for $\geq$2 consecutive days in the summer, the warm season and the whole year)	lag 0-3	RR during heat waves compared to non-heat wave days	sex, age	Non-accidental and circulatory mortality significantly increased across the 3 cities under different heat wave definitions and study periods. Using the summer data resulted in the largest increase in effect estimates compared use of the warm season or whole year data.
Wang et al. 2012	Jan. 1996 - Nov. 2004	Brisbane, Australia	non-external (<800, A00-R99); cardiovascuar (390-459, I00-I99); respiratory (460-519, J00-J99)	heat waves	heat waves (daily max ≥37°C for $\geq$2 consecutive days)	lag 1, 2, 0-2	ORs comparing heat waves to non-heat waves	age	non-external mortality (OR 1.46, 95% CI 1.21-1.77), CVD mortality (1.89, 1.44-2.48)
Williams et al. 2012	July 1993 - Mar. 2009	Adelaide, South Australia	all-cause	heat waves	hot days as max temperature >90th, 95th, or 99th percentile for warm season	not specified	risk for hot days compared to all other days during the warm season	age	Associations between temperature over thresholds and daily mortality not statistically significant when adjusted for ozone and PM_{10}; at extreme temperatures mortality increased significantly with increasing heat wave duration.
Wilson et al. 2013	July 1997 - Dec. 2007	Sydney Greater Metropolitan Region, Australia	all-cause (A00-R99); CVD (I00-I99); respiratory (J00-J99)	heat waves	heat waves: daily max T_{app}, daily max temperature >95th percentile	lag 0 to lag 3, lag 0-2	heat-event days compared to non-heat event days	age	All-cause mortality had similar magnitude associations with single day and 3 day extreme and severe events as did CVD mortality. Respiratory mortality associated with single day and 3 day severe events (95th percentile, lag 0 OR1.14 (95% CI 1.04-1.24).

Xu and Tong 2017	Nov.-Mar., 1988-2011	Sydney, Melbourne and Brisbane, Australia	non-accidental	heat waves	HW 4 types: 1) Type I: extremely-hot days followed by extremely-hot nights (HW_{both}); 2) Type II: extremely-hot days followed by not-extremely-hot nights (HW_{day}); 3) Type III: not extremely-hot days followed by extremely-hot nights (HW_{night}); and 4) Type IV:not-extremely-hot days followed by not-extremely-hot nights (HW_{warm}); extremely-hot temperature refers to temperature at least ≥96th percentile of the monthly temperature distribution and 3 percentiles (96th, 97th, and 98th) were used as the intensity, and 3 durations (2, 3 and 4 days) were used as the duration.	lag 0-7	risk during heat wave days compared to non-heat wave days	HW intensity, duration	Mortality in Brisbane increased significantly during HW_{both} and HW_{warm}, and mortality in Melbourne increased significantly during HW_{both} and HW_{day}. For Sydney, HW_{both}, HW_{warm}, and HW_{day} were associated with mortality increase, although no appreciable difference in the magnitudes of mortality increase among these 3 heatwave types was observed. HW_{night} was not associated with significant mortality increase in these cities. Mean temperature was the best temperature indicator for heatwaves in Brisbane and T_{max} in Melbourne.
**South America**									
Son et al. 2016a	May 1996-December 2010	São Paulo, Brazil	all-cause (A00-R99); CVD (I00-I99); respiratory (J00-J99)	heat waves	heat wave as ≥2 consecutive days with daily mean temperatures ≥96th percentile warm season temperature	lag 0	risk comparing heat wave and non-heat wave days	sex, age, education, marital status, and place of death, heat wave characteristics	Risks higher for females and those with no education for heat effect, and males for cold effect. Older persons, widows, and non-hospital deaths had higher mortality risks for heat and cold.
**Multi-country studies**									

Guo et al. 2017	limited to hot season (4 hottest adjacent months) for each community; Australia (3 cities 1988–2008), Brazil (18 cities 1997–2011), Canada (26 cities 1986–2009), China (6 cities 2002–07), Colombia (5 cities 1998–2013), Iran (1 city 2004– 13), Ireland (all Irish Island national data, including 6 regions: 2 in Northern Ireland and 4 in Republic of Ireland 1984–2007), Italy (10 cities 1987–2010), Japan (47 prefectures, 1985–2012), Moldova (4 cities 2001–2010), Philippines (4 cities during 2006–2010), South Korea (7	400 communities in 18 countries/regions	all causes/nonaccidental	heat waves	12 types of heat waves by combining community-specific daily mean temperature ≥90th, 92.5th, 95th, and 97.5th percentiles of temperature with duration ≥2, 3, and 4 d	lag 0-10	risk during heat wave days comapred to non-heat wave days	heat wave characteristics (intensity and duration); regional differences (4 groups (cold, moderate cold, moderate hot, and hot areas) by the quantiles of temperature based on the hot season mean temperature distribution of all 400 communities); community-specific hot season avg temperature, temperature range, temperature variability, latitude, longitude	Heat waves of all definitions had significant cumulative associations with mortality in all countries, but varied by community. Higher heat wave associations with higher temperature thresholds used to define heat waves. Heat wave duration did not modify the impacts. Association between heat waves and mortality appeared acutely and lasted for 3 and 4 d. Heat waves had higher associations with mortality in moderate cold and moderate hot areas than cold and hot areas. There were no added effects of heat waves on mortality in all countries/regions, except for Brazil, Moldova, and Taiwan. Heat waves defined by daily mean and Tmax produced similar heat wave-mortality

D'Ippoliti et al. 2010	June-Aug., 1990-2004	9 European cities	all natural causes (1-799, group A-R); CVD (390-459, group I); respiratory (460-519, group J)	heat waves	heat waves (considered both max T_{app} and min temperature, 1) $\geq$2 days with max T_{app} >90th percentile of monthly distribution or 2) >2 days with Tmin > 90th percentile and max T_{app} > median monthly value)	not specified	risk during heat wave days compared to non-heat wave days	sex, age	Large geographical heterogeneity of effects among cities; Considering all years, except 2003, mortality increase ranged from 7.6% in Munich to 33.6% in Milan; The increase was up to 3-times greater during heat waves of long duration and high intensity; higher pooled impact in Mediterranean (21.8% for total mortality) than in North Continental (12.4%) cities.

Note: ICD codes includes ICD 9 and/or ICD 10; Lag means days unless otherwise specified; We reported main findings regardless of statistical significance, presented by study authors, as originally reported

Table ~~S3~~S4. Disease categories and diagnosis codes in studies

Mortality	Diagnosis codes	% of studies
Total	ICD-10 A00-R99	24.7
	ICD-10 A00-R99 or ICD-9 1-799	14.3
	ICD-9 1-799	9.3
	ICD-10 A00-U99	4.9
	ICD-10 A00-U99 or ICD-9 1-799	2.2
	Else[a]	4.9
	No ICD codes provided	39.6
Cardiovascular	ICD-10 I00-I99	45.5
	ICD-10 I00-I99 or ICD-9 390-459	21.4
	ICD-9 390-459	8.9
	ICD-10 I00-I79 or ICD-9 390-459	3.6
	ICD-9 390-448	1.8
	ICD-9 390-429	1.8
	ICD-9 AM 25-32	1.8
	Else[b]	10.7
	No ICD codes provided	4.5
Respiratory	ICD-10 J00-J99	38.2
	ICD-10 J00-J99 or ICD-9 460-519	24.7
	ICD-9 460-519	15.7
	ICD-10 J00-J98	5.6
	ICD-10 J00-J98 or ICD-9 460-519	4.5
	ICD-9 AM 33-35	2.2
	Else[c]	4.5
	No ICD codes provided	4.5

Note: One study can be included in multiple disease categories.

[a] 000+, A00+; A00-S99; 1-799, 992, E900.0, A-R, T67, X30; A00-Z99; A00-R99, T67, X30, X32, X54; 1-799, 900; 0-999; A00-Y98, 000-999; A00-R99, V01-V99, W00-X59; A00-R94

[b] I60-I69; 390-429, I00-I52; I00-I79; 390-499, I00-I99; 390-448, I00-I79; 390-429, I01-I51; 390-429, I01-I52; 390-398, 402, 404, 410-419, 420-429, I00-I11.9, I13.0-I13.9, I20.0-I59.9; 402-429; 430-438; 380-459; 393-429, I05-I52

[c] J00-J98, U04; 460-466, 480-487, 490-492, 494-496, J9-J18, J40-J44, J47; 480-486, 490-497, 507; 460-496, 507

Table ~~S4~~S5. Summary of scientific evidence for effect modification on the temperature-mortality association

Effect modifier	Heat waves	Cold spells	Summary of evidence
Others (modification of exposure)			
Heat wave/cold spell characteristics			Limited or suggestive evidence of higher risk with heat wave characteristics
Intensity	4 studies ↑	1 study ↑	
Duration	3 studies ↑; 1 study -	1 study ↑	
Timing in season	3 studies ↑	1 study ↑	

Abbreviations for Table ~~S1~~ S2 and Table ~~S2~~S3

AC: air conditioning

AF: attributable fraction

AIC: Akaike Information Criterion

AR: attributable risk

avg: average

CI: confidence interval

COPD: chronic obstructive pulmonary disease

CRR: cumulative relative risk

CSD: circulatory system disease

CVD: cardiovascular disease

CWI: cold wave index

DM: diabetes mellitus

DT: dry tropical

DTR: diurnal temperature range

EH: extreme heat

EQI: environmental quality indices

GDP: Gross Regional Domestic Product

ICD: International Classification of Diseases

IQR: interquartile range

Min: minimum temperature

MMT: minimum mortality temperature

MT+: moist tropical plus

NDVI: Normalized Difference Vegetation Index

NEM: non-external mortality

NI: Northern Ireland

OR: odds ratio

PI: posterior interval

PT: perceived temperature

RH: relative humidity

ROI: Republic of Ireland

RR: relative risk

SD: standard deviation

SEP: socioeconomic position

SSC: spatial synoptic classification

T_{app}: apparent temperature

TCN: temperature change between neighboring days

TDI: temperature deviation index

Tmax: maximum temperature

TV: temperature variability (variation)

UHII: urban heat island index

UK: United Kingdom

US: United States

UTCI: Universal thermal climate index

yrs: years

References

Ahmadnezhad E, Holakouie Naieni K, Ardalan A, et al. Excess mortality during heat waves, Tehran Iran: An ecological time-series study. J Res Health Sci. 2013;13(1):24-31.

Almeida S, Casimiro E, Analitis A. Short-term effects of summer temperatures on mortality in Portugal: A time-series analysis. J Toxicol Environ Health A. 2013;76(7):422-428.

Almeida SP, Casimiro E, Calheiros J. Effects of apparent temperature on daily mortality in Lisbon and Oporto, Portugal. Environ Health. 2010;9:12-18.

Analitis A, Katsouyanni K, Biggeri A, et al. Effects of cold weather on mortality: Results from 15 European cities within the PHEWE Project. Am J Epidemiol. 2008;168:1397-1408.

Anderson GB, Bell ML. Heat waves in the United States: Mortality risk during heat waves and effect modification by heat wave characteristics in 43 U.S. communities. Environ Health Perspect. 2011;119:210-218.

Andersona BG, Bell ML. Weather-related mortality: How heat, cold, and heat waves affect mortality in the United States. Epidemiology. 2009;20(2):205-213.

Antunes L, Silva SP, Marques J, et al. The effect of extreme cold temperatures on the risk of death in the two major Portuguese cities. Int J Biometeorol. 2017;61(1):127-135.

Azongo DK, Awine T, Wak G, et al. A time series analysis of weather variability and all-cause mortality in the Kasena-Nankana Districts of Northern Ghana, 1995-2010. Glob Health Action. 2012;5:14-22.

Baccini M, Biggeri A, Accetta G, et al. Heat effects on mortality in 15 European cities. Epidemiology. 2008;19:711-719.

Baccini M, Kosatsky T, Analitis A, et al. Impact of heat on mortality in 15 European cities: attributable deaths under different weather scenarios. J Epidemiol Community Health. 2011;65(1):64-70.

Bai L, Cirendunzhu, Woodward A, et al. Temperature and mortality on the roof of the world: A time-series analysis in three Tibetan counties, China. Sci Total Environ. 2014;485-486:41-48.

Ballester F, Corella D, Pérez-Hoyos S, et al. Mortality as a function of temperature A Study in Valencia, Spain, 1991-1993. Int J Epidemiol. 1997;26:551-561.

Ban J, Xu D, He MZ, et al. The effect of high temperature on cause-specific mortality: A multi-county analysis in China. Environ Int. 2017;106:19-26.

Barnett AG, Hajat S, Gasparrini A, et al. Cold and heat waves in the United States. Environ Res. 2012;112:218-224.

Basagaña X, Sartini C, Barrera-Gómez J, et al. Heat waves and cause-specific mortality at all ages. Epidemiology. 2011;22:765-772.

Basu R, Malig B. High ambient temperature and mortality in California: Exploring the roles of age, disease, and mortality displacement. Environ Res. 2011;111:1286-1292.

Basu R, Pearson D, Sie L, et al. A case-crossover study of temperature and infant mortality in California. Paediatr Perinat Epidemiol. 2015;29:407-415.

Bell ML, O'Neill MS, Ranjit N, et al. Vulnerability to heat-related mortality in Latin America: a case-crossover study in São Paulo, Brazil, Santiago, Chile and Mexico City, Mexico. Int J Epidemiol. 2008;37:796-804.

Bobb JF, Dominici F, Peng RD. A Bayesian model averaging approach for estimating the relative risk of mortality associated with heat waves in 105 U.S. cities. Biometrics. 2011;67(4):1605-1616.

Borrell C, Marí-Dell'Olmo M, Rodríguez-Sanz M, et al. Socioeconomic position and excess mortality during the heat wave of 2003 in Barcelona. Eur J Epidemiol. 2006;21:633-640.

Braga AL, Zanobetti A, Schwartz J. The effect of weather on respiratory and cardiovascular deaths in 12 U.S. cities. Environ Health Perspect. 2002;110:859-863.

Breitner S, Wolf K, Devlin RB, et al. Short-term effects of air temperature on mortality and effect modification by air pollution in three cities of Bavaria, Germany: A time-series analysis. Sci Total Environ. 2014a;485-486:49-61.

Breitner S, Wolf K, Peters A, et al. Short-term effects of air temperature on cause-specific cardiovascular mortality in Bavaria, Germany. Heart. 2014b;100:1272-1280.

Burkart K, Breitner S, Schneider A, et al. An analysis of heat effects in different subpopulations of Bangladesh. Int J Biometeorol. 2014;58:227-237.

Burkart K, Meier F, Schneider A, et al. Modification of heat-related mortality in an elderly urban population by vegetation (urban green) and proximity to water (urban blue): evidence from Lisbon, Portugal. Environ Health Perspect. 2016;124:927-934.

Cagle A, Hubbard R. Cold-related cardiac mortality in King County, Washington, USA 1980-2001. Ann Hum Biol. 2005;32(4):525-537.

Carson C, Hajat S, Armstrong B, et al. Declining vulnerability to temperature-related mortality in London over the 20th Century. Am J Epidemiol. 2006;164:77-84.

Chen K, Bi J, Chen J, et al. Influence of heat wave definitions to the added effect of heat waves on daily mortality in Nanjing, China. Sci Total Environ. 2015;506-507:18-25.

Chen K, Zhou L, Chen X, et al. Urbanization level and vulnerability to heat-related mortality in Jiangsu Province, China. Environ Health Perspect. 2016;124(12):1863-1869.

Chen TH, Li X, Zhao J, et al. Impacts of cold weather on all-cause and cause-specific mortality in Texas, 1990-2011. Environ Pollut. 2017;225:244-251.

Cheng J, Xu Z, Bambrick H, et al. The mortality burden of hourly temperature variability in five capital cities, Australia: Time-series and meta-regression analysis. Environ Int. 2017;109:10-19.

Cheng J, Zhu R, Xu Z, et al. Temperature variation between neighboring days and mortality: a distributed lag non-linear analysis. Int J Public Health. 2014;59:923-931.

Chung Y, Noh H, Honda Y, et al. Temporal changes in mortality related to extreme temperatures for 15 Cities in Northeast Asia: Adaptation to heat and maladaptation to cold. Am J Epidemiol. 2017;185(10):907-913.

Cui Y, Yin F, Deng Y, et al. Heat or cold: Which one exerts greater deleterious effects on health in a Basin Climate City? Impact of ambient temperature on mortality in Chengdu, China. Int J Environ Res Public Health. 2016;13(12):1225-1236.

Curriero FC, Heiner KS, Samet JM, et al. Temperature and mortality in 11 cities of the eastern United States. Am J Epidemiol. 2002;155:80-87.

Dang TN, Seposo XT, Duc NH, et al. Characterizing the relationship between temperature and mortality in tropical and subtropical cities: a distributed lag non-linear model analysis in Hue, Viet Nam, 2009-2013. Glob Health Action. 2016;9:28738.

de' Donato FK, Leone M, Scortichini M, et al. Changes in the effect of heat on mortality in the last 20 years in nine European cities. Results from the PHASE Project. Int J Environ Res Public Health. 2015;12:15567-15583.

Díaz J, Linares C, Tobías A. Impact of extreme temperatures on daily mortality in Madrid (Spain) among the 45-64 age-group. Int J Biometeorol. 2006;50:342-348.

Diboulo E, Sié A, Rocklöv J, et al. Weather and mortality: a 10 year retrospective analysis of the Nouna Health and Demographic Surveillance System, Burkina Faso. Glob Health Action. 2012;5:6-13.

Ding Z, Li L, Wei R, et al. Association of cold temperature and mortality and effect modification in the subtropical plateau monsoon climate of Yuxi, China. Environ Res. 2016a;150:431-437.

Ding Z, Li L, Xin L, et al. High diurnal temperature range and mortality: Effect modification by individual characteristics and mortality causes in a case-only analysis. Sci Total Environ. 2016b;544:627-634.

D'Ippoliti D, Michelozzi P, Marino C, et al. The impact of heat waves on mortality in 9 European cities: results from the EuroHEAT project. Environ Health. 2010;9:37-45.

Donaldson GC, Keatinge WR. Cold related mortality in England and Wales; influence of social class in working and retired age groups. J Epidemiol Community Health. 2003;57:790-791.

El-Zeina A, Tewtel-Salemb M, Nehmec G. A time-series analysis of mortality and air temperature in Greater Beirut. Sci Total Environ. 2004;330:71-80.

Gao H, Lan L, Yang C, et al. The threshold temperature and lag effects on daily excess mortality in Harbin, China: A time series analysis. Int J Occup Environ Med. 2017;8(2):85-95.

Gasparrini A, Armstrong B, Kovats S, et al. The effect of high temperatures on cause-specific mortality in England and Wales. Occup Environ Med. 2012;69:56-61.

Goggins WB, Chan EYY, Ng E, et al. Effect modification of the association between short-term meteorological factors and mortality by urban heat islands in Hong Kong. PLoS ONE. 2012;7(6):e38551.

Goggins WB, Ren C, Ng E, et al. Effect modification of the association between meteorological variables and mortality by urban climatic conditions in the tropical city of Kaohsiung, Taiwan. Geospatial Health. 2013;8(1):37-44.

Goldberg MS, Gasparrini A, Armstrong B, et al. The short-term influence of temperature on daily mortality in the temperate climate of Montreal, Canada. Environ Res. 2011;111:853-860.

Gómez-Acebo I, Dierssen-Sotos T, Llorca J. Effect of cold temperatures on mortality in Cantabria (Northern Spain): A case-crossover study. Public Health. 2010;124(7):398-403.

Gómez-Acebo I, Llorca J, Rodríguez-Cundín P, et al. Extreme temperatures and mortality in the North of Spain. Int J Public Health. 2012;57:305-313.

Goodman PG, Dockery DW, Clancy L. Cause-specific mortality and the extended effects of particulate pollution and temperature exposure. Environ Health Perspect. 2004;112:179-185.

Gouveia N, Hajat S, Armstrong B. Socioeconomic differentials in the temperature-mortality relationship in São Paulo, Brazil. Int J Epidemiol. 2003;32:390-397.

Gronlund CJ, Berrocal VJ, White-Newsome JL, et al. Vulnerability to extreme heat by socio-demographic characteristics and area green space among the elderly in Michigan, 1990-2007. Environ Res. 2015;136:449-461.

Guo Y, Barnett AG, Yu W, et al. A large change in temperature between neighbouring days increases the risk of mortality. PLoS ONE. 2011;6(2):e16511.

Guo Y, Gasparrini A, Armstrong B, et al. Global variation in the effects of ambient temperature on mortality: a systematic evaluation. Epidemiology. 2014;25(6):781-789.

Guo Y, Gasparrini A, Armstrong BG, et al. Heat wave and mortality: A multicountry, multicommunity study. Environ Health Perspect. 2017;125(8):087006.

Guo Y, Punnasiri K, Tong S. Effects of temperature on mortality in Chiang Mai city, Thailand: a time series study. Environ Health. 2012;1:36-44.

Ha J, Kim H, Hajat S. Effect of previous-winter mortality on the association between summer temperature and mortality in South Korea. Environ Health Perspect. 2011a;119:542-546.

Ha J, Kim H. Changes in the association between summer temperature and mortality in Seoul, South Korea. Int J Biometeorol. 2013;57:535-544.

Ha J, Shin Y, Kim H. Distributed lag effects in the relationship between temperature and mortality in three major cities in South Korea. Sci Total Environ. 2011b;409:3274-3280.

Ha J, Yoon J, Kim H. Relationship between winter temperature and mortality in Seoul, South Korea, from 1994 to 2006. Sci Total Environ. 2009;407:2158-2164.

Hajat S, Armstrong BG, Gouveia N, et al. Mortality displacement of heat-related deaths A comparison of Delhi, São Paulo, and London. Epidemiology. 2005;16:613-620.

Hajat S, Chalabi Z, Wilkinson P, et al. Public health vulnerability to wintertime weather: time-series regression and episode analyses of national mortality and morbidity databases to inform the Cold Weather Plan for England. Public Health. 2016;137:26-34.

Hajat S, Kovats RS, Lachowycz K. Heat-related and cold-related deaths in England and Wales: who is at risk? Occup Environ Med. 2007;64:93-100.

Hales S, Salmond C, Town GI, et al. Daily mortality in relation to weather and air pollution in Christchurch, New Zealand. Aust N Z J Public Health. 1999;24:89-91.

Han J, Liu S, Zhang J, et al. The impact of temperature extremes on mortality: a time-series study in Jinan, China. BMJ Open. 2017;7(4):e014741.

Harlan SL, Chowell G, Yang S, et al. Heat-related deaths in hot cities: Estimates of human tolerance to high temperature thresholds. Int J Environ Res Public Health. 2014;11:3304-3326.

Hashizume M, Wagatsuma Y, Hayashi T, et al. The effect of temperature on mortality in rural Bangladesh-a population-based time-series study. Int J Epidemiol. 2009;38:1689-1697.

Heo S, Lee E, Kwon BY, et al. Long-term changes in the heat-mortality relationship according to heterogeneous regional climate: a time-series study in South Korea. BMJ Open. 2016;6(8):e011786.

Ho HC, Knudby A, Walker BB, et al. Delineation of spatial variability in the temperature-mortality relationship on extremely hot days in Greater Vancouver, Canada. Environ Health Perspect. 2017;125(1):66-75.

Huang J, Wang J, Yu W. The lag effects and vulnerabilities of temperature effects on cardiovascular disease mortality in a subtropical climate zone in China. Int J Environ Res Public Health. 2014;11:3982-3994.

Huang W, Kan H, Kovats S. The impact of the 2003 heat wave on mortality in Shanghai, China. Sci Total Environ. 2010;408:2418-2420.

Huang Z, Lin H, Liu Y, et al. Individual-level and community-level effect modifiers of the temperature–mortality relationship in 66 Chinese communities. BMJ Open. 2015;5:e009172. doi:10.1136/bmjopen-2015-009172

Hutter HP, Moshammer H, Wallner P, et al. Heatwaves in Vienna: effects on mortality. Wien Klin Wochenschr. 2007;119(7-8):223-227.

Huynen MM, Martens P, Schram D, et al. The impact of heat waves and cold spells on mortality rates in the Dutch population. Environ Health Perspect. 2001;109:463-470.

Ingole V, Juvekar S, Muralidharan V, et al. The short-term association of temperature and rainfall with mortality in Vadu Health and Demographic Surveillance System: a population level time series analysis. Glob Health Action. 2012;5(1):19118.

Ingole V, Kovats S, Schumann B, et al. Socioenvironmental factors associated with heat and cold-related mortality in Vadu HDSS, western India: a population-based case-crossover study. Int J Biometeorol. 2017;61(10):1797-1804.

Ingole V, Rocklöv J, Juvekar S, et al. Impact of heat and cold on total and cause-specific mortality in Vadu HDSS—A rural setting in western India. Int J Environ Res Public Health. 2015;12:15298-15308.

Iñiguez C, Ballester F, Ferrandiz J, et al. Relation between temperature and mortality in thirteen Spanish cities. Int J Environ Res Public Health. 2010;7:3196-3210.

Isaksen TB, Fenske RA, Hom EK, et al. Increased mortality associated with extreme-heat exposure in King County, Washington, 1980-2010. Int J Biometeorol. 2016;60(1):85-98.

Ishigami A, Hajat S, Kovats RS, et al. An ecological time-series study of heat-related mortality in three European cities. Environ Health. 2008;7:5.

Jian Y, Wu CYH, Gohlke JM. Effect modification by environmental quality on the association between heatwaves and mortality in Alabama, United States. Int J Environ Res Public Health. 2017;14(10).

Joe L, Hoshiko S, Dobraca D, et al. Mortality during a large-scale heat wave by place, demographic group, internal and external causes of death, and building climate zone. Int J Environ Res Public Health. 2016;13(3):299-314.

Kaiser R, Le Tertre A, Schwartz J, et al. The effect of the 1995 heat wave in Chicago on all-cause and cause-specific mortality. Am J Public Health. 2007;97:S158-S162.

Kan H, London SJ, Chen H, et al. Diurnal temperature range and daily mortality in Shanghai, China. Environ Res. 2007;103:424-431.

Kent ST, McClure LA, Zaitchik BF, et al. Heat waves and health outcomes in Alabama (USA): the importance of heat wave definition. Environ Health Perspect. 2014;122:151-158.

Kim H, Ha JS, Park J. High temperature, heat index, and mortality in 6 major cities in South Korea. Arch Environ Occup Health. 2006;61(6):265-270.

Kim H, Heo J, Kim H, et al. Has the impact of temperature on mortality really decreased over time? Sci Total Environ. 2015;512-513:74-81.

Kim J, Shin J, Lim YH, et al. Comprehensive approach to understand the association between diurnal temperature range and mortality in East Asia. Sci Total Environ. 2016;539:313-321.

Kim Y, Gasparrini A, Hashizume M, et al. Heat-related mortality in Japan after the 2011 Fukushima Disaster: An analysis of potential influence of reduced electricity consumption. Environ Health Perspect. 2017;125(7):077005.

Kim Y, Joh S. A vulnerability study of the low-income elderly in the context of high temperature and mortality in Seoul, Korea. Sci Total Environ. 2006;371:82-88.

Kim YM, Kim S, Cheong HK, et al. Comparison of temperature indexes for the impact assessment of heat stress on heat-related mortality. Environ Health Toxicol. 2011;26:e2011009.

Kunst AE, Looman CW, Mackenbach JP. Outdoor air temperature and mortality in the Netherlands: A time-series analysis. Am J Epidemiol. 1993;137:331-41.

Kysely J, Pokorna L, Kyncl J, et al. Excess cardiovascular mortality associated with cold spells in the Czech Republic. BMC Public Health. 2009;9:19-29.

Lan L, Cui G, Yang C, et al. Increased mortality during the 2010 heat wave in Harbin, China. EcoHealth. 2012;9:310-314.

Lee M, Shi L, Zanobetti A, et al. Study on the association between ambient temperature and mortality using spatially resolved exposure data. Environ Res. 2016;151:610-617.

Li J, Xu X, Yang J, et al. Ambient high temperature and mortality in Jinan, China: A study of heat thresholds and vulnerable populations. Environ Res. 2017;156:657-664.

Li L, Yang J, Guo C, et al. Particulate matter modifies the magnitude and time course of the non-linear temperature-mortality association. Enviro Pollut. 2015;196:423-430.

Li Y, Cheng Y, Cui G, et al. Association between high temperature and mortality in metropolitan areas of four cities in various climatic zones in China: a time-series study. Environ Health. 2014;13:65-74.

Lim YH, Bell ML, Kan H, et al. Economic status and temperature-related mortality in Asia. Int J Biometeorol. 2015;59:1405-1412.

Lin YK, Ho TJ, Wang YC. Mortality risk associated with temperature and prolonged temperature extremes in elderly populations in Taiwan. Environ Res. 2011;111:1156-1163.

Lindeboom W, Alam N, Begum D, et al. The association of meteorological factors and mortality in rural Bangladesh, 1983-2009. Glob Health Action. 2012;5:61-73.

Liu L, Breitner S, Pan X, et al. Associations between air temperature and cardiorespiratory mortality in the urban area of Beijing, China: a time-series analysis. Environ Health. 2011;10:51-61.

Luo K, Li R, Wang Z, et al. Effect modification of the association between temperature variability and daily cardiovascular mortality by air pollutants in three Chinese cities. Environ Pollut. 2017;230:989-999.

Ma W, Chen R, Kan H. Temperature-related mortality in 17 large Chinese cities: How heat and cold affect mortality in China. Environ Res. 2014;134:127-133.

Ma W, Yang C, Chu C, et al. The impact of the 2008 cold spell on mortality in Shanghai, China. Int J Biometeorol. 2013;57:179-184.

Ma W, Yang C, Tan J, et al. Modifiers of the temperature–mortality association in Shanghai, China. Int J Biometeorol. 2012;56:205-207.

Ma W, Zeng W, Zhou M, et al. The short-term effect of heat waves on mortality and its modifiers in China: An analysis from 66 communities. Environ Int. 2015;75:103-109.

Mackenbach JP, Looman CW, Kunst AE. Air pollution, lagged effects of temperature, and mortality: The Netherlands 1979-87. J Epidemiol Community Health. 1993;47:121-126.

Madrigano J, Ito K, Johnson S, et al. A case-only study of vulnerability to heat wave–related mortality in New York City (2000–2011). Environ Health Perspect. 2015a;123:672-678.

Madrigano J, Jack D, Anderson GB, et al. Temperature, ozone, and mortality in urban and non-urban counties in the northeastern United States. Environ Health. 2015b;14:3-13.

Medina-Ramón M, Schwartz J. Temperature, temperature extremes, and mortality: a study of acclimatisation and effect modification in 50 US cities. Occup Environ Med. 2007;64:827-833.

Medina-Ramón M, Zanobetti A, Cavanagh DP, et al. Extreme temperatures and mortality: Assessing effect modification by personal characteristics and specific cause of death in a multi-city case-only analysis. Environ Health Perspect. 2006;114:1331-1336.

Metzger KB, Ito K, Matte TD. Summer heat and mortality in New York City: How hot is too hot? Environ Health Perspect. 2010;118:80-86.

Milojevic A, Armstrong BG, Gasparrini A, et al. Methods to estimate acclimatization to urban heat island effects on heat- and cold-related mortality. Environ Health Perspect. 2016;124(7):1016-1022.

Monteiro A, Carvalho V, Oliveira T, et al. Excess mortality and morbidity during the July 2006 heat wave in Porto, Portugal. Int J Biometeorol. 2013;57:155-167.

Morabito M, Crisci A, Moriondo M, et al. Air temperature-related human health outcomes: Current impact and estimations of future risks in Central Italy. Sci Total Environ. 2012;441:28-40.

Nitschke M, Tucker GR, Bi P. Morbidity and mortality during heatwaves in metropolitan Adelaide. Med J Aust. 2007;187:662-665.

Nitschke M, Tucker GR, Hansen AL, et al. Impact of two recent extreme heat episodes on morbidity and mortality in Adelaide, South Australia: a case-series analysis. Environ Health. 2011;10:42-50.

Nordio F, Zanobetti A, Colicino E, et al. Changing patterns of the temperature-mortality association by time and location in the US, and implications for climate change. Environ Int. 2015;81:80-86.

O'Neill MS, Hajat S, Zanobetti A, et al. Impact of control for air pollution and respiratory epidemics on the estimated associations of temperature and daily mortality. Int J Biometeorol. 2005a;50:121-129.

O'Neill MS, Zanobetti A, Schwartz J. Disparities by race in heat-related mortality in four US Cities: The role of air conditioning prevalence. J Urban Health. 2005b;82(2):191-197.

Onozuka D, Hagihara A. Variation in vulnerability to extreme-temperature-related mortality in Japan: A 40-year time-series analysis. Environ Res. 2015;140:177-184.

Oudin Åström D, Åström C, Rekker K, et al. High summer temperatures and mortality in Estonia. PLoS One. 2016;11(5):e0155045.

Oudin Åström D, Schifano P, Asta F, et al. The effect of heat waves on mortality in susceptible groups: a cohort study of a mediterranean and a northern European City. Environ Health. 2015;14:30-37.

Pattenden S, Armstrong B, Milojevic A, et al. Ozone, heat and mortality: acute effects in 15 British conurbations. Occup Environ Med. 2010;67:699-707.

Petkova EP, Gasparrini A, Kinney PL. Heat and mortality in New York City since the beginning of the 20th century. Epidemiology. 2014;25(4):554-560.

Qiao Z, Guo Y, Yu W, et al. Assessment of short- and long-term mortality displacement in heat-related deaths in Brisbane, Australia, 1996-2004. Environ Health Perspect. 2015;123:766-772.

Qiu H, Tian L, Ho KF, et al. Who is more vulnerable to death from extremely cold temperatures? A case-only approach in Hong Kong with a temperate climate. Int J Biometeorol. 2016;60(5):711-717.

Rabczenko D, Wojtyniak B, Kuchcik M, et al. Association between high temperaturę and mortality of Warsaw inhabitants, 2008-2013. Przegl Epidemiol. 2016;70(4):629-640.

Ragettli MS, Vicedo-Cabrera AM, Schindler C, et al. Exploring the association between heat and mortality in Switzerland between 1995 and 2013. Environ Res. 2017;158:703-709.

Rainham DG, Smoyer-Tomic KE. The role of air pollution in the relationship between a heat stress index and human mortality in Toronto. Environ Res. 2003;93:9-19.

Ren C, Williams GM, Morawska L, et al. Ozone modifies associations between temperature and cardiovascular mortality: analysis of the NMMAPS data. Occup Environ Med. 2008;65:255-260.

Revich B, Shaposhnikov D. Excess mortality during heat waves and cold spells in Moscow, Russia. Occup Environ Med 2008a;65:691-696.

Revich B, Shaposhnikov D. Temperature-induced excess mortality in Moscow, Russia. Int J Biometeorol. 2008b;52:367-374.

Revich BA, Shaposhnikov DA. Extreme temperature episodes and mortality in Yakutsk, East Siberia. Rural Remote Health. 2010;10(2):1338.

Rocklöv J, Ebi K, Forsberg B. Mortality related to temperature and persistent extreme temperatures: a study of cause-specific and age-stratified mortality. Occup Environ Med. 2011;68:531-536.

Rocklöv J, Forsberg B, Ebi K, et al. Susceptibility to mortality related to temperature and heat and cold wave duration in the population of Stockholm County, Sweden. Glob Health Action. 2014;7:22737.

Rocklöv J, Forsberg B, Meister K. Winter mortality modifies the heat-mortality association the following summer. Eur Respir J. 2009;33:245-251.

Rocklöv J, Forsberg B. The effect of high ambient temperature on the elderly population in three regions of Sweden. Int J Environ Res Public Health. 2010;7:2607-2619.

Rocklöv J, Forsberg B. The effect of temperature on mortality in Stockholm 1998-2003: A study of lag structures and heatwave effects. Scand J Public Health. 2008;36:516-523.

Rosenthal JK, Kinney PL, Metzger KB. Intra-urban vulnerability to heat-related mortality in New York City, 1997-2006. Health Place. 2014;30:45-60.

Schaeffer L, de Crouy-Chanel P, Wagner V, et al. How to estimate exposure when studying the temperature-mortality relationship? A case study of the Paris area. Int J Biometeorol. 2016;60(1):73-83.

Schifano P, Cappai G, De Sario M, et al. Susceptibility to heat wave-related mortality: a follow-up study of a cohort of elderly in Rome. Environ Health. 2009;8:50-63.

Scovronick N, Armstrong B. The impact of housing type on temperature-related mortality in South Africa, 1996-2015. Environ Res. 2012;113:46-51.

Seposo XT, Dang TN, Honda Y. Evaluating the effects of temperature on mortality in Manila City (Philippines) from 2006-2010 Using a distributed lag nonlinear model. Int J Environ Res Public Health. 2015;12:6842-6857.

Sharafkhani R, Khanjani N, Bakhtiari B, et al. Diurnal temperature range and mortality in Urmia, the Northwest of Iran. J Therm Biol. 2017;69:281-287.

Sheridan SC, Lin S. Assessing variability in the impacts of heat on health outcomes in New York City over time, season, and heat-wave duration. EcoHealth. 2014;11:512-525.

Smargiassi A, Goldberg MS, Plante C, et al. Variation of daily warm season mortality as a function of micro-urban heat islands. J Epidemiol Community Health. 2009;63:659-664.

Son JY, Gouveia N, Bravo MA, et al. The impact of temperature on mortality in a subtropical city: Effects of cold, heat, and heat waves in São Paulo, Brazil. Int J Biometeorol. 2016a;60(1):113-121.

Son JY, Lane KJ, Lee JT, et al. Urban vegetation and heat-related mortality in Seoul, Korea. Environ Res. 2016b;151:728-733.

Son JY, Lee JT, Anderson GB, et al. Vulnerability to temperature-related mortality in Seoul, Korea. Environ Res Lett. 2011;6:034027.

Son JY, Lee JY, Anderson GB, et al. The impact of heat waves on mortality in seven major cities in Korea. Environ Health Perspect. 2012;120:566-571.

Stafoggia M, Forastiere F, Agostini D, et al. Factors affecting in-hospital heat-related mortality: a multi-city case-crossover analysis. J Epidemiol Community Health. 2008;62:209-215.

Stafoggia M, Forastiere F, Agostini D, et al. Vulnerability to heat-related mortality A Multicity, population-based, case-crossover analysis. Epidemiology. 2006;17:315-323.

Stafoggia M, Forastiere F, Michelozzi P, et al. Summer temperature-related mortality effect modification by previous winter mortality. Epidemiology. 2009;20:575-583.

Sun S, Tian L, Qiu H, et al. The influence of pre-existing health conditions on short-term mortality risks of temperature: Evidence from a prospective Chinese elderly cohort in Hong Kong. Environ Res. 2016;148:7-14.

Sun X, Sun Q, Zhou X, et al. Heat wave impact on mortality in Pudong New Area, China in 2013. Sci Total Environ. 2014;493:789-794.

Sung TI, Wu PC, Lung SC, et al. Relationship between heat index and mortality of 6 major cities in Taiwan. Sci Total Environ. 2013;442:275-281.

The Eurowinter Group. Cold exposure and winter mortality from ischaemic heart disease, cerebrovascular disease, respiratory disease, and all causes in warm and cold regions of Europe. Lancet. 1997;349:1341-1346.

Tobías A, Armstrong B, Gasparrini A, et al. Effects of high summer temperatures on mortality in 50 Spanish cities. Environ Health. 2014;13:48-53.

Tong S, FitzGerald G, Wang XY, et al. Exploration of the health risk-based definition for heatwave: A multi-city study. Environ Res. 2015;142:696-702.

Tong S, Wang XY, FitzGerald G, et al. Development of health risk-based metrics for defining a heatwave: a time series study in Brisbane, Australia. BMC Public Health. 2014a;14:435-444.

Tong S, Wang XY, Yu W, et al. The impact of heatwaves on mortality in Australia: a multicity study. BMJ Open. 2014b;4(2):e003579.

Urban A, Davídkovová H, Kyselý J. Heat- and cold-stress effects on cardiovascular mortality and morbidity among urban and rural populations in the Czech Republic. Int J Biometeorol. 2014;58:1057-1068.

Urban A, Hanzlíková H, Kyselý J, et al. Impacts of the 2015 heat waves on mortality in the Czech Republic-A comparison with previous heat waves. Int J Environ Res Public Health. 2017;14(12):1562-1580.

Vaneckova P, Beggs PJ, de Dear RJ, et al. Effect of temperature on mortality during the six warmer months in Sydney, Australia, between 1993 and 2004. Environ Res. 2008;108:361-369.

Vaneckova P, Beggs PJ, Jacobson CR. Spatial analysis of heat-related mortality among the elderly between 1993 and 2004 in Sydney, Australia. Soc Sci Med. 2010;70:293-304.

Vigotti MA, Muggeo VM, Cusimano R. The effect of birthplace on heat tolerance and mortality in Milan, Italy, 1980-1989. Int J Biometeorol. 2006;50:335-341.

Wang C, Chen R, Kuang X, et al. Temperature and daily mortality in Suzhou, China: A time series analysis. Sci Total Environ. 2014;466-467:985-990.

Wang C, Zhang Z, Zhou M, et al. Nonlinear relationship between extreme temperature and mortality in different temperature zones: A systematic study of 122 communities across the mainland of China. Sci Total Environ. 2017;586:96-106.

Wang L, Liu T, Hu M, et al. The impact of cold spells on mortality and effect modification by cold spell characteristics. Sci Rep. 2016;6:38380.

Wang X, Li G, Liu L, et al. Effects of extreme temperatures on cause-specific cardiovascular mortality in China. Int J Environ Res Public Health. 2015;12:16136-16156.

Wang XY, Barnett AG, Yu W, et al. The impact of heatwaves on mortality and emergency hospital admissions from non-external causes in Brisbane, Australia. Occup Environ Med. 2012;69:163-169.

Wang XY, Guo Y, FitzGerald G, et al. The impacts of heatwaves on mortality differ with different study periods: A multi-city time series investigation. PLoS ONE. 2015;10(7):e0134233.

Wichmann J, Andersen ZJ, Ketzel M, et al. Apparent temperature and cause-specific mortality in Copenhagen, Denmark: A case-crossover analysis. Int J Environ Res Public Health. 2011;8:3712-3727.

Wichmann J. Heat effects of ambient apparent temperature on all-cause mortality in Cape Town, Durban and Johannesburg, South Africa: 2006-2010. Sci Total Environ. 2017;587-588:266-272.

Williams S, Nitschke M, Sullivan T, et al. Heat and health in Adelaide, South Australia: Assessment of heat thresholds and temperature relationships. Sci Total Environ. 2012;414:126-133.

Wilson LA, Morgan GG, Hanigan IC, et al. The impact of heat on mortality and morbidity in the Greater Metropolitan Sydney Region: a case crossover analysis. Environ Health. 2013;12:98-111.

Wu W, Xiao Y, Li G, et al. Temperature–mortality relationship in four subtropical Chinese cities: A time-series study using a distributed lag non-linear model. Sci Total Environ. 2013;449:355-362.

Xiao J, Peng J, Zhang Y, et al. How much does latitude modify temperature–mortality relationship in 13 eastern US cities? Int J Biometeorol. 2015;59:365-372.

Xie H, Yao Z, Zhang Y, et al. Short-term effects of the 2008 cold spell on mortality in three subtropical cities in Guangdong Province, China. Environ Health Perspect. 2013;121:210-216.

Xu W, Thach TQ, Chau YK, et al. Thermal stress associated mortality risk and effect modification by sex and obesity in an elderly cohort of Chinese in Hong Kong. Environ Pollut. 2013;178:288-293.

Xu Y, Dadvand P, Barrera-Gómez J, et al. Differences on the effect of heat waves on mortality by sociodemographic and urban landscape characteristics. J Epidemiol Community Health. 2013;67:519-525.

Xu Z, Tong S. Decompose the association between heatwave and mortality: Which type of heatwave is more detrimental? Environ Res. 2017;156:770-774.

Yang J, Liu HZ, Ou CQ, et al. Impact of heat wave in 2005 on mortality in Guangzhou, China. Biomed Environ Sci. 2013;26(8):647-654.

Yang J, Ou CQ, Ding Y, et al. Daily temperature and mortality: a study of distributed lag non-linear effect and effect modification in Guangzhou. Environ Health. 2012;11:63-71.

Yang J, Yin P, Zhou M, et al. Cardiovascular mortality risk attributable to ambient temperature in China. Heart. 2015;101:1966-1972.

Yi W, Chan AP. Effects of temperature on mortality in Hong Kong: a time series analysis. Int J Biometeorol. 2015;59:927-936.

Yin Q, Wang J. The association between consecutive days' heat wave and cardiovascular disease mortality in Beijing, China. BMC Public Health. 2017;17(1):223-231.

Yu W, Guo Y, Ye X, et al. The effect of various temperature indicators on different mortality categories in a subtropical city of Brisbane, Australia. Sci Total Environ. 2011a;409:3431-3437.

Yu W, Hu W, Mengersen K, et al. Time course of temperature effects on cardiovascular mortality in Brisbane, Australia. Heart. 2011b;97:1089-1093.

Yu W, Mengersen K, Hu W, et al. Assessing the relationship between global warming and mortality: Lag effects of temperature fluctuations by age and mortality categories. Environ Pollut. 2011c;159:1789-1793.

Yu W, Vaneckova P, Mengersen K, et al. Is the association between temperature and mortality modified by age, gender and socio-economic status? Sci Total Environ. 2010;408:3513-3518.

Zanobetti A, Schwartz J. Temperature and mortality in nine US cities. Epidemiology. 2008;19(4):563-570.

Zeka A, Browne S, McAvoy H, et al. The association of cold weather and all-cause and cause-specific mortality in the island of Ireland between 1984 and 2007. Environ Health. 2014;13:104-112.

Zeng J, Zhang X, Yang J, et al. Humidity may modify the relationship between temperature and cardiovascular mortality in Zhejiang Province, China. Int J Environ Res Public Health. 2017;14(11):1383-1393.

Zeng W, Lao X, Rutherford S, et al. The effect of heat waves on mortality and effect modifiers in four communities of Guangdong Province, China. Sci Total Environ. 2014;482-483:214-221.

Zhan Z, Zhao Y, Pang S, et al. Temperature change between neighboring days and mortality in United States: A nationwide study. Sci Total Environ. 2017;584-585:1152-1161.

Zhang J, Li TT, Tan JG, et al. Impact of temperature on mortality in three major Chinese cities. Biomed Environ Sci. 2014;27(7):485-494.

Zhang J, Liu S, Han J, et al. Impact of heat waves on nonaccidental deaths in Jinan, China, and associated risk factors. Int J Biometeorol. 2016;60(9):1367-1375.

Zhang K, Chen TH, Begley CE. Impact of the 2011 heat wave on mortality and emergency department visits in Houston, Texas. Environ Health. 2015;14:11-17.

Zhang Y, Yu C, Bao J, et al. Impact of temperature variation on mortality: An observational study from 12 counties across Hubei Province in China. Sci Total Environ. 2017a;587-588:196-203.

Zhang Y, Yu C, Yang J, et al. Diurnal temperature range in relation to daily mortality and years of life lost in Wuhan, China. Int J Environ Res Public Health. 2017b;14(8):891-901.

Zhou MG, Wang LJ, Liu T, et al. Health impact of the 2008 cold spell on mortality in subtropical China: the climate and health impact national assessment study (CHINAs). Environ Health. 2014;13:60-72.
